# Snapshots of the Reaction Coordinate of a Thermophilic 2′-Deoxyribonucleoside/ribonucleoside Transferase

**DOI:** 10.1021/acscatal.3c06260

**Published:** 2024-02-13

**Authors:** Peijun Tang, Christopher J. Harding, Alison L. Dickson, Rafael G. da Silva, David J. Harrison, Clarissa Melo Czekster

**Affiliations:** † School of Biology, Biomedical Sciences Research Complex, University of St Andrews, St Andrews, Fife KY16 9ST, United Kingdom; ‡ School of Medicine, University of St Andrews, North Haugh, St Andrews KY16 9TF, United Kingdom

**Keywords:** deoxyribonucleoside transferase, nucleosides, biocatalysis, protein engineering, thermophilic

## Abstract

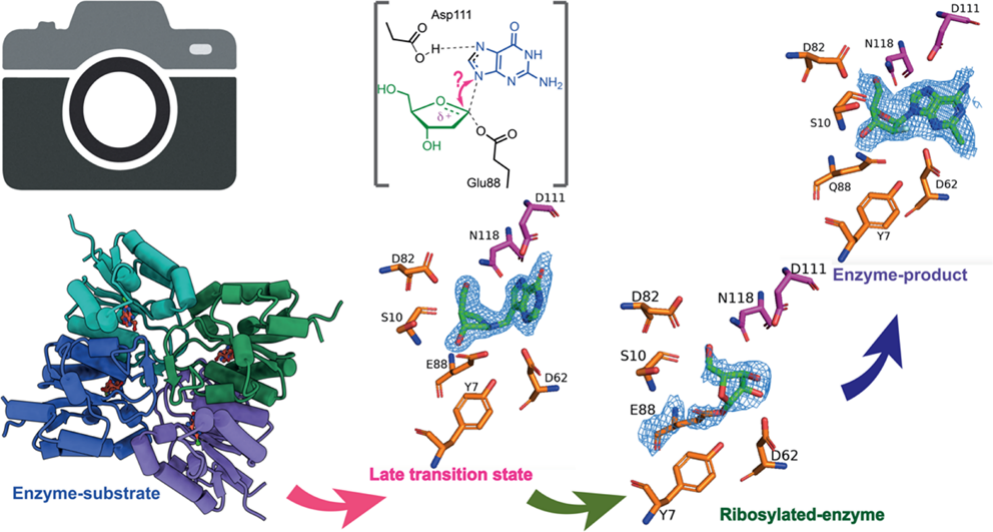

Nucleosides are ubiquitous to life and are required for the synthesis of DNA, RNA, and other molecules crucial for cell survival. Despite the notoriously difficult organic synthesis of nucleosides, 2′-deoxynucleoside analogues can interfere with natural DNA replication and repair and are successfully employed as anticancer, antiviral, and antimicrobial compounds. Nucleoside 2′-deoxyribosyltransferase (dNDT) enzymes catalyze transglycosylation via a covalent 2′-deoxyribosylated enzyme intermediate with retention of configuration, having applications in the biocatalytic synthesis of 2′-deoxynucleoside analogues in a single step. Here, we characterize the structure and function of a thermophilic dNDT, the protein from *Chroococcidiopsis thermalis* (*Ct*NDT). We combined enzyme kinetics with structural and biophysical studies to dissect mechanistic features in the reaction coordinate, leading to product formation. Bell-shaped pH-rate profiles demonstrate activity in a broad pH range of 5.5–9.5, with two very distinct p*K*_a_ values. A pronounced viscosity effect on the turnover rate indicates a diffusional step, likely product (nucleobase1) release, to be rate-limiting. Temperature studies revealed an extremely curved profile, suggesting a large negative activation heat capacity. We trapped a 2′-fluoro-2′-deoxyarabinosyl-enzyme intermediate by mass spectrometry and determined high-resolution structures of the protein in its unliganded, substrate-bound, ribosylated, 2′-difluoro-2′-deoxyribosylated, and in complex with probable transition-state analogues. We reveal key features underlying (2′-deoxy)ribonucleoside selection, as *Ct*NDT can also use ribonucleosides as substrates, albeit with a lower efficiency. Ribonucleosides are the building blocks of RNA and other key intracellular metabolites participating in energy and metabolism, expanding the scope of use of *Ct*NDT in biocatalysis.

## Introduction

Nucleosides are the building blocks of DNA and RNA and are key molecules for metabolism and energetics. Nucleoside analogues have been extensively used to interfere with biological processes governing nucleotide incorporation into DNA and RNA, successfully targeting viral and cellular replication^1^ and yielding a plethora of drugs against cancer, herpes simplex, HIV, and hepatitis C.^2^ However, overcoming synthetic challenges, specifically creating concise and adaptable synthetic pathways, remains a challenge.^3^ Enzymes present a promising strategy to generate nucleosides in a stereospecific manner, with fewer steps and the potential to utilize a single catalyst for the production of multiple products.^4−11^ Recent work employing solely biocatalysis to produce the nucleoside drugs Islatravir^4^ and Molnupiravir,^5^ as well as the key modified RNA precursor pseudouridine,^6^ has further highlighted the power of enzymes in the production of a series of therapeutically relevant nucleosides.

**Scheme 1:**
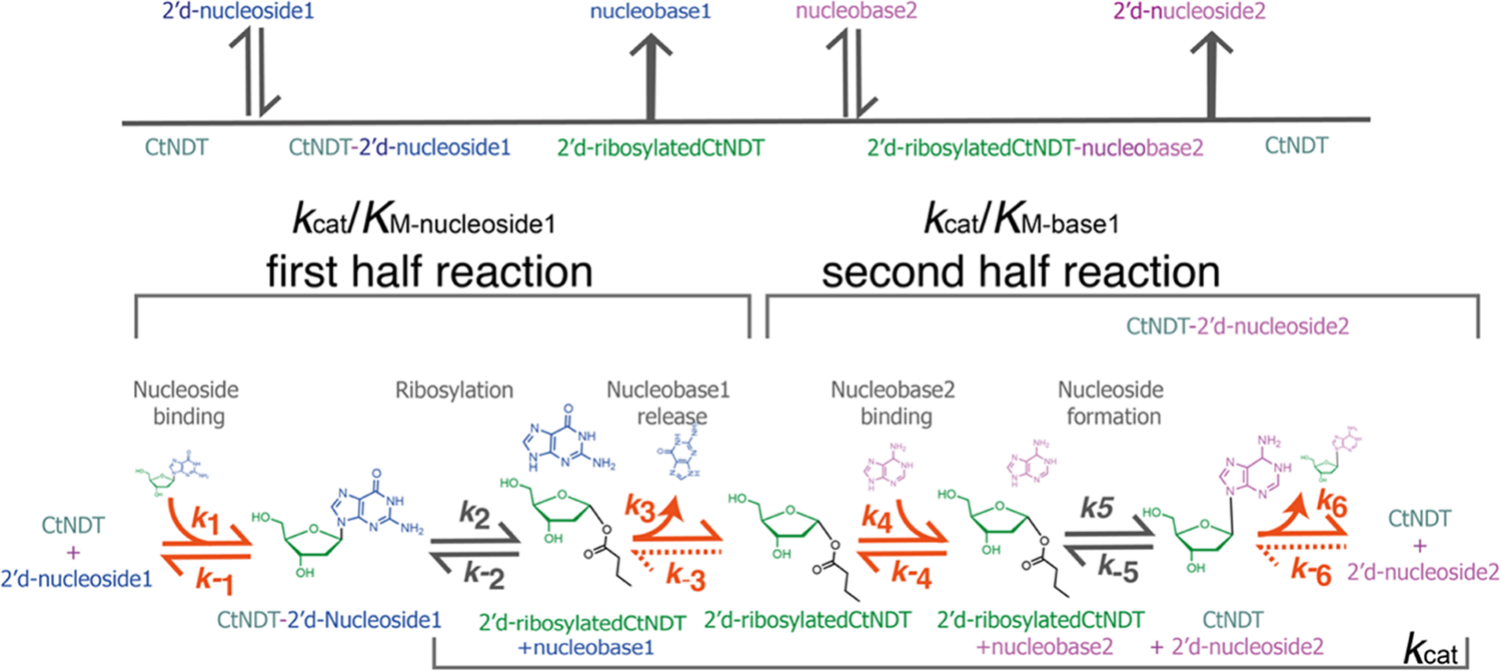
Top: Ping-Pong Reaction Catalyzed by dNDT Enzymes. Bottom: Steps Included in Kinetic Parameters under Evaluation for C*t*NDT A full turnover cycle includes nucleobase transfer to generate a novel 2′-deoxyribonucleoside product. Diffusional steps are shown with orange arrows. Under steady-state initial rate conditions, dashed arrows depicting *k*_–3_ and *k*_–6_ are ≅0.

Nucleoside 2′-deoxyribosyltransferases (EC 2.4.2.6, dNDTs) participate in nucleoside recycling in organisms that lack purine and pyrimidine nucleoside phosphorylases.^12^ In these organisms, dNDTs play a crucial role in scavenging deoxyribonucleosides for DNA synthesis. The reaction catalyzed by dNDT proceeds via a ping-pong mechanism, with the formation of a 2′-deoxyribosyl-enzyme intermediate covalently linked to an active-site glutamate (Scheme 1).^13^ Transglycosylation occurs in a stereospecific manner, preserving the anomeric carbon in the β configuration in the newly formed 2′-deoxyribonucleoside. Enzymes from this class have strict specificity for 2′-deoxyribonucleosides^14,15^ and have been employed as biocatalysts to synthesize a large number of 2′-deoxyribonucleoside analogues with potential biomedical applications.^7−11^ dNDTs have also been investigated as model systems to understand glycosyl transfer and principles of catalysis in sugar-transfer reactions.^16−18^ Prior quantum mechanics–molecular mechanics (QM/MM) free-energy landscape calculations with a dNDT enzyme supported an oxocarbenium ion intermediate in the reaction,^19^ but no empirical evidence was available shedding light on catalysis and the transition state for this reaction.

The thermal adaptation and plasticity present in thermostable enzymes are key to molecular adaptations to heat and other environmental stressors.^20^ Despite a preliminary characterization of the first thermostable dNDT enzyme, details on the kinetics, structure, and substrate selection by this thermostable enzyme are lacking. Preliminary work proposed the dNDT from *Chroococcidiopsis thermalis* PCC 7203 (*Ct*NDT) as a promising tool in biocatalysis to produce novel 2′-deoxynucleosides.^21^*Chroococcidiopsis* spp. occur in a wide range of extreme temperature and salinity environments, being highly desiccation resistant.^22^ Thermostability is a desirable feature in biocatalysis,^23^ given that many chemical transformations will require high temperatures, contamination is less widespread, and substrate solubility is increased at high temperatures.^24^

Here, we dissected the mechanism, rate-limiting steps, and kinetic and structural determinants of *Ct*NDT’s catalyzed reaction. We obtained snapshots of different steps in the reaction coordinate using X-ray crystallography, including a ribosyl–C*t*NDT covalent intermediate and the tight-binding interaction to a potential transition-state analogue, further characterized by isothermal titration calorimetry. Steady-state and pre-steady-state kinetics of *Ct*NDT and mutants uncovered specific interactions and rate-limiting steps in nucleoside transfer, highlighting a significant contribution of negative activation heat capacity for turnover and key amino-acid residues mediating substrate selection, transition-state stabilization, and catalysis. This work sets the stage for the engineering of *Ct*NDT toward the synthesis of novel (2′-deoxy)nucleosides of pharmaceutical interest.

## Materials and General Methods

The codon-optimized synthetic gene was ordered from Integrated DNA Technologies (IDT). General chemicals and reagents were from Fluorochem, Merck, and Fisher Scientific. Data were fitted using GraphPad Prism. Pre-steady-state data were fitted using KinTek Global Explorer.^25^ Additional methods for protein production, enzymatic assays, mass spectrometry, high-performance liquid chromatography (HPLC) conditions, and differential scanning fluorimetry (DSF) experiments are available in the Supporting Information.

### Cloning, Expression, and Purification of *Ct*NDT and Its Mutants

The synthetic gene encoding the*C. thermalis* NDT (Uniprot code: K9TVX3) was ordered as a Gblock (Integrated DNA Technologies) and cloned into a pJ411 expression plasmid with a cleavable 6-histidine tag at the N-terminus by Gibson assembly. Construct design was carried out using NEBuilder (New England Biolabs), following the cloning protocol suggested to amplify the plasmid pJ411 backbone as well as the GBlock to generate an overlap of 20bp between sequences. Following cloning, the gene sequence was confirmed by sequencing (Eurofins). pJ411::*Ct*NDT was transformed into *Escherichia coli* BL21 (DE3) cells for overexpression in LB medium with 50 μg mL^–1^ kanamycin at 37 °C with shaking at 180 rpm until cells reached OD_600_ = 0.8. Protein expression was induced by addition of 0.5 mM IPTG overnight at 16 °C while being shaken at 180 rpm. The cells were harvested by centrifugation at 12,000*g* for 20 min, resuspended in wash buffer (50 mM MES, 250 mM NaCl, 30 mM imidazole, pH 6.5), and lysed using a cell homogenizer (Constant Systems). Following centrifugation for 30 min at 51,000*g* at 4 °C, the supernatant was filtered with a 0.8 mm filter to remove particulates and loaded onto a 5 mL HisTrap column pre-equilibrated with wash buffer. The column was washed with 10 column volumes of wash buffer, and *Ct*NDT was eluted with 50 mM MES, 250 mM NaCl, and 500 mM imidazole, pH 6.5. Fractions containing *Ct*NDT were pooled and dialyzed with 2 mg mL^–1^ tobacco etch virus (TEV) protease (prepared in-house)^26^ in buffer (50 mM MES, 250 mM NaCl, pH 6.5) overnight at 4 °C. The dialyzed mixture was loaded onto a 5 mL HisTrap column, and the flow-through fractions were collected and analyzed by sodium dodecyl-sulfate polyacrylamide gel electrophoresis (SDS-PAGE). Fractions containing purified *Ct*NDT were pulled, flash-frozen, and stored at −80 °C (Figure S1). The mutants *Ct*NDT_D62N_, *Ct*NDT_E88Q_, *Ct*NDT_E88A_, and *Ct*NDT_M120C_ were expressed and purified using the same method. Protein concentration was determined by the extinction coefficient at 280 nm calculated using the ExPASy ProtParam tool.^27^ Intact protein mass spectra for all proteins produced are shown in Figure S2.

### Standard Activity Assay for *Ct*NDT

#### (2′-Deoxy)nucleoside Substrates

A standard assay contained *Ct*NDT (between 1 nM and 1 μM), a 2′-deoxynucleoside (10–5000 μM), and a nucleobase (10–2.5 mM) in a mixed buffer solution (a final volume of 50 μL and the final concentration of the mixed buffer was 30 mM for each individual buffer component (CHES, MES, and HEPES, pH 8.5) incubated at 45 °C.

#### Ribonucleoside Substrates

A standard assay contained *Ct*NDT (1 μM), 10 μM–1000 mM ribonucleoside (Ado, Gua, or Ino), and a nucleobase (10 mM Hyp or Ade) in a mixed buffer solution (50 μL and the final concentration of the mixed buffer was 30 mM for each individual buffer component (CHES, MES, and HEPES), pH 7) incubated at 65 °C.

For all substrates tested, 50 μL reaction mixture was quenched with 200 μL of 10 M urea at 5, 10, and 15 min, followed by centrifugation at 24,000*g* for 10 min. Half of this volume (100 μL) was placed into a 96-well round-bottom microplate (Agilent Technologies), and 10 μL was injected onto the HPLC column (HSS T3, Waters employing a Shimadzu Prominence HPLC and the method described in “HPLC Conditions for Reaction Monitoring” in the Supporting Information). All experiments were carried out in duplicate. Product formation was quantified using calibration curves with reference standard compounds (Figure S5) for each 2′-deoxyribonucleoside or ribonucleoside product and conversion of the integrated area values into concentration. Data were fitted using the Michaelis–Menten equation in GraphPad Prism, and the kinetic parameters were plotted for substrates or enzyme variants. Individual concentrations of enzymes and substrates used for different experiments are described in the Supporting Information. Retention times for reference standard compounds were obtained by running each standard (see the table in Figure S5).

### Enzyme Assays of *Ct*NDT with Varying pH and Temperature Values

Assays contained *Ct*NDT (5 nM), 10–1000 μM dGuo, and 10 mM Ade in a mixed buffer solution (50 μL total; 30 mM CHES, MES, and HEPES each) in a range of pH values from 5 to 9.5 at 0.5 pH unit intervals. Prior to varying temperature and/or pH, protein stability in the conditions required was verified. For that, the protein was preincubated for 15 min in test assay buffer under the conditions to be tested (varying pH or varying temperature), after which the protein was diluted in the activity assay (at 45 °C pH 8.5), and activity was monitored. This activity test was compared to the protein preincubated under the standard conditions, and only pH and temperature values in which activity was unchanged from control were used. For temperature studies, reactions were carried out using CHES, MES, and HEPES (30 mM each; pH 8.5) and incubated from 35 to 65 °C at 5 °C intervals. To obtain reaction rates, 50 μL reaction mixture was quenched with 200 μL of 10 M urea at 5, 10, and 15 min, followed by centrifugation at 24,000*g* for 10 min. Analysis proceeded by HPLC as described above.

### Fast Kinetics

Assays of *Ct*NDT under pre-steady-state conditions were carried out with 2′-dAdo or immucillin-H (ImmH), monitoring tryptophan fluorescence emission with an excitation wavelength of 280 nm and an emission wavelength above 320 nm (using a cutoff filter) in an Applied Photophysics SX-20 stopped-flow spectrophotometer containing a 5 μL mixing cell (0.5 cm path length, and 0.9 ms dead time) at 25 °C. The assay buffer contained 50 mM MES and 250 mM NaCl, pH 6.5. One syringe contained 1.5 μM *Ct*NDT (or mutants), and the other contained 0–200 μM 2′-dAdo. The reaction was triggered by mixing 55 μL from each syringe by the stopped-flow instrument with an applied pressure hold. The fluorescence decrease was measured for 1 s with 4000 data points collected per trace. At least 3 traces were averaged per condition and used for data fitting. Data were analyzed using KinTek Global Explorer 10.^25,28,29^ As controls, readings with the substrate or enzyme in buffer were performed in the concentration range utilized for each experiment. The maximum concentration used of ligands was 100 μM to avoid inner filter effects, in agreement with prior experiments.^30^

### Crystallization of *Ct*NDT

Recombinant *Ct*NDT was purified and concentrated to ∼10 mg mL^–1^ for use in crystallization experiments. Proteins were crystallized using the sitting-drop vapor diffusion technique (using a 1:1 ratio of protein to reservoir). Crystals formed after 1 week of incubation at 20 °C. For ligand cocrystallization experiments, the enzyme was incubated with 1 mM ligand (Ado for the ribosylated structure, Clofarabine, DADmeH, or ImmH) at 4 °C for 20 min prior to screening. Crystals were cryoprotected in mother liquor supplemented with 20% ethylene glycol and flash-cooled prior to data collection at Diamond Light Source (beamline I03 or I04). Crystallization conditions are given in Table S6.

### Data Collection, Structure Determination, Model Building, Refinement, and Validation

Following diffraction data collection, data reduction and processing were carried out with the automated processing pipeline at Diamond with XIA2 or DIALS. The structure of wild-type *Ct*NDT was solved by molecular replacement with PHASER.^31^ Search components comprised an ensemble of other NDTs (6EVS, 6QAI, 2F62 and 3EHD) and further modified using sculptor. Wild-type *Ct*NDT was used as a search model for complex structures. Buccaneer^32^ was used to trace chains and build the initial models. Refinement was carried out with Phenix.refine,^31^ and manual model building with Coot.^33^ The model was validated using Coot and Molprobity^34^ online. Figures were made using ChimeraX^35^ and Pymol (Schrodinger, version 2.5.0). Crystallographic data are shown in Table S7. All coordinates and structure factor files have been deposited in the Protein Data Bank (PDB accession codes 8PQP, 8PQQ, 8PQR, 8PQS, 8PQT, 8QC0, 8RH3).

### Equations for Data Fitting

pH data were plotted with pH and the log kinetic parameter. Data for pH-rate profiles were fitted to eq 1, where *C* is the pH-independent value of *k*_cat_; p*K*_a1_ and p*K*_a2_ are the dissociation constants for ionizable groups.
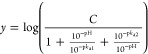
1The entire range of temperatures showing a nonlinear temperature dependence was fitted to an Eyring equation accounting for heat capacity (*C*_p_^‡^) (eq 2),^36−38^ where *R* is the gas constant; *T* is the temperature (K); *T*_0_ is a reference temperature value (45 °C or 318 K was used); *k* is the kinetic rate constant or parameter; Δ*H*^‡^ is the enthalpy of activation; Δ*C*_p_^‡^ is the activation heat capacity; *k*_B_ is the Boltzmann constant; *h* is Planck’s constant; and Δ*S*^‡^ is the entropy of activation.
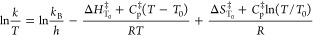
2Fitted values for kinetic rate constants were converted into energy barriers using eq 3
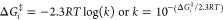
3where Δ*G*_*i*_^‡^ is the Gibbs free energy of activation for step *i*, *R* is the gas constant, *T* is the temperature (K), and *k* is the rate constant for step *i*.

For all experiments performed in duplicate, values are reported as the mean and standard error of the mean. As a note of caution, these error values are not strictly speaking a true estimate of error (which would require data at least in triplicate) but an indication of experimental variability. Whenever possible, individual replicate values are shown.

## Results and Discussion

### pH Dependence of the Reaction

For *Ct*NDT, *k*_cat_/*K*_M-nucleoside1_ encompasses all steps from free enzyme and free substrate up to and including the first irreversible step (Scheme 1). For the first half-reaction, this is the release of the first nucleobase product (nucleobase1), while for the second half-reaction, this is the release of the newly formed nucleoside (nucleoside2). *k*_cat_ includes all steps after nucleoside1 binding, up to and including regeneration of the free enzyme. In *Ct*NDT’s case, our HPLC assays monitored the formation of a new nucleoside product (nucleoside2), and therefore, *k*_cat_ includes the release of nucleobase1 and nucleoside2 from the enzyme (Scheme 1). Protein production, purity, and molecular weight determination are available in Figure S1.

**Figure 1:**
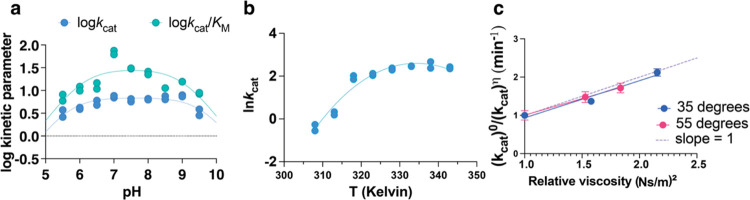
Effect of pH, temperature, and viscosity on the *Ct*NDT-catalyzed reaction. (a) pH dependence of the *Ct*NDT-catalyzed reaction. The line is a fit to eq 1, yielding acid dissociation constants for *k*_cat_ (p*K*_a1_ = 5.8 ± 0.1, p*K*_a2_ = 9.7 ± 0.2, *R*^2^ = 0.8) and for *k*_cat_/*K*_M-2′dGuo_ (p*K*_a1_ = 6.1 ± 0.3, p*K*_a2_ = 9.0 ± 0.2; *R*^2^ = 0.6). Figure S4D depicts residual plots for fits. (b) Temperature dependence of *k*_cat_; data were fitted to eq 2, yielding a Δ*C*_p_^‡^ of −1.8 ± 0.3 kcal mol^–1^ K^–1^, and at 45 °C, a Δ*H*^‡^ of 28.9 ± 2.2 kcal mol^–1^ and a Δ*S*^‡^ of 0.032 ± 0.006 kcal mol^–1^. *R*^2^ = 0.95. Figure S4E depicts residual plots for fit. (c) Solvent viscosity effect on *k*_cat_ using glycerol as a microviscogen at two different temperatures. The dotted line is shown for reference as a reaction completely limited by a diffusional step, with slope = 1. Here, the slope at 35 °C equals 1.0 ± 0.2, and the slope at 55 °C equals 0.9 ± 0.1. A control experiment was performed with PEG 8000, and no effect was observed (Figure S4C). Data for (a–c) were obtained using 2′dGuo and Ade as substrates. For parts (a, b), duplicates are shown; for part (c), data were collected in triplicate and are shown as mean plus standard error of the mean.

pH-rate profiles for *Ct*NDT were bell-shaped for *k*_cat_ (p*K*_a1_ = 5.8 ± 0.1, p*K*_a2_ = 9.7 ± 0.2) and for *k*_cat_/*K*_M-2′dGuo_ (p*K*_a1_ = 6.1 ± 0.3, p*K*_a2_ = 9.0 ± 0.2) with a single group in each limb giving rise to the pH dependence in both cases (Figure 1a).

p*K*_a1_: The p*K*_a1_ for *k*_cat_/*K*_M-2′dGuo_ and *k*_cat_ profiles are likely reporting on the protonation state of E88, which must be deprotonated for catalysis to occur,^39^ in agreement with *Lactobacillus leishmanii* (*Ll*NDT).^40^

p*K*_a2_: The p*K*_a2_ of ∼9.0 could be reporting on the p*K*_a_ of D111 (discussed below), which could be donating a proton to the nucleobase leaving group, facilitating departure, a step included in both *k*_cat_/*K*_M-2′dGuo_ and *k*_cat_. The environment surrounding D111 could determine its p*K*_a2_; however, no other negatively charged residues in the vicinity explain this large shift from the typical p*K*_a_ of an aspartate residue.^41^

Alternatively, neighboring groups have been proposed to facilitate substrate binding and/or catalysis.^40,42^ Schramm proposes that a drastic increase in the p*K*_a_ from around 2 to 9 in the case of protonation of N7 of inosine and purine nucleobases more generally occurs upon C1′ bond cleavage during the reaction catalyzed by purine nucleoside phosphorylase (PNP).^27^ Since there are similarities between PNP- and *Ct*NDT-catalyzed reactions, an analogous p*K*_a_ shift may occur here, enabling nucleobase leaving group stabilization by D111. The p*K*_a_ for the N7 of guanosine in solution is between 3 and 4.^43^ Y7 is in close proximity to catalytic E88, and its deprotonation would decrease its nucleophilicity, impacting turnover.^26^

A comparison of the residues in the vicinity of the active site does not provide any obvious rationale for the shifted p*K*_a_ values when comparing *Ll*NDT and *Ct*NDT (Figure S14), but the stickiness of substrates^44^ and rate-limiting product release^45^ can lead to outward shifted p*K*_a_ values when comparing for *k*_cat_ in comparison to *k*_cat_/*K*_M-2′dGuo_.

### Temperature-Rate Profiles and Negative Heat Capacity

Figure 1b depicts the temperature dependence of *k*_cat_. The clearly curved temperature dependence profile was the best fit of the data to eq 2, yielding a Δ*C*_p_^‡^ of −1.8 ± 0.3 kcal mol^–1^ K^–1^, and at 45 °C, a Δ*H*^‡^ of 28.9 ± 2.2 kcal mol^–1^ and a Δ*S*^‡^ of 0.032 ± 0.006 kcal mol^–1^.

Careful consideration was taken to exclude factors other than activation heat capacity, which could lead to nonlinear dependence. *Ct*NDT was not denatured in the range evaluated here (between 35 and 65 °C), as the melting temperature for *Ct*NDT was determined to be 72 °C under the conditions employed (pH 7.0) using differential scanning fluorimetry (Figure S3).

Changes in the rate-limiting steps across the temperature range under evaluation can lead to curved profiles. We carried out viscosity studies to determine whether the rate-limiting step was changing in the temperature range in which the curvature is observed.^37^ Using different concentrations of glycerol and determining *k*_cat_ at 35 and 55 °C revealed a large and equal viscosity effect on the two temperatures investigated, with a slope of 1 within the experimental error (Figures 1c and S4). Furthermore, in the viscosity studies, we ensured experimentally, by varying substrates near the saturation range, that the enzyme was saturated with both substrates, and the lack of response in rate with increasing temperature was not due to sub-*V*_max_ rates.

Such a high viscosity effect points toward a diffusional step, likely nucleobase1 release (further discussed below under “transient kinetics”), as a limiting step for turnover at both temperatures,^37,46,47^ excluding changes in the rate-limiting step between 35 and 55 °C as a reason for deviations from classic Arrhenius behavior. We performed a viscosity experiment with 5% PEG 8000,^48^ to probe whether a “medium” or crowding effect was taking place. No viscosity effect was observed with PEG (Figure S4C), further corroborating the importance of diffusion steps for *k*_cat_.

Prior work on *Ct*NDT indicated a nonlinear dependence of rate as a function of temperature.^21^ These studies did not evaluate enzyme stability, change of rate-limiting steps, and kinetic parameters in the temperature range under investigation, and therefore, conclusions on the origin of nonlinear curves could not be drawn. Enzymes that display classic Arrhenius behavior have rates of reaction that increase exponentially as a function of temperature until denaturation starts to occur.^49^ The change in activation heat capacity ( Δ*C*_p_^‡^) associated with enzyme catalysis^50^ has been invoked to explain deviations from classic Arrhenius behavior.

A negative Δ*C*_p_^‡^ suggests a decrease in the number of vibrational modes as the enzyme–substrate complex approaches the transition state.^36,50^ As illustrated in Scheme 1, *k*_cat_ contains physical steps (substrate binding and product release, as well as potential conformational changes not shown in the figure for simplicity) and chemical steps,^38^ all of which can contribute to the effect observed on Δ*C*_p_^‡^. Because the viscosity effect is close to 1 in the temperature range under study, the transition state in this case is not the chemical TS but that for the diffusional release of the product.

Another possibility has been put forward to account for nonclassic Arrhenius behavior in the reaction catalyzed by an α-amylase from a psychrophilic organism, invoking the presence of different conformational states in the ES complex, giving rise to changes of opposite magnitudes to Δ*H*^‡^ and −*T*Δ*S*^‡^.^51,52^ In this particular system, Δ*G*^‡^ had sizable contributions from Δ*H*^‡^ and −*T*Δ*S*^‡^ at different temperatures, whereas in the *Ct*NDT-catalyzed reaction, at 45 °C, −*T*Δ*S*^‡^ is negligible, while Δ*H*^‡^ is 28.9 kcal mol^–1^. and therefore, it is unlikely that changes in the magnitude of thermodynamic parameters could lead to the curvature observed. Therefore, after ruling out denaturation, subsaturation, and changes in the rate-limiting step, a negative Δ*C*_p_^‡^ remains the most likely model to account for nonclassic Arrhenius behavior in the reaction catalyzed by *Ct*NDT.

The microorganism producing *Ct*NDT, *C. thermalis*, is adapted for growth in desertic arid environments with high optimal temperatures.^22^ The optimal temperature of enzymes often correlates with environmental temperatures surrounding their organisms of origin,^53^ and heat capacity has been suggested to influence transition-state stabilization at high temperatures, as well as protein adaptability^54^ and evolvability.^55^ Negative Δ*C*_p_^‡^ can reflect a higher preorganization state of *Ct*NDT as it approaches the reaction transition state, providing advantages to catalysis at high temperatures.

### Substrate Specificity of *Ct*NDT and Mutants

**Figure 2:**
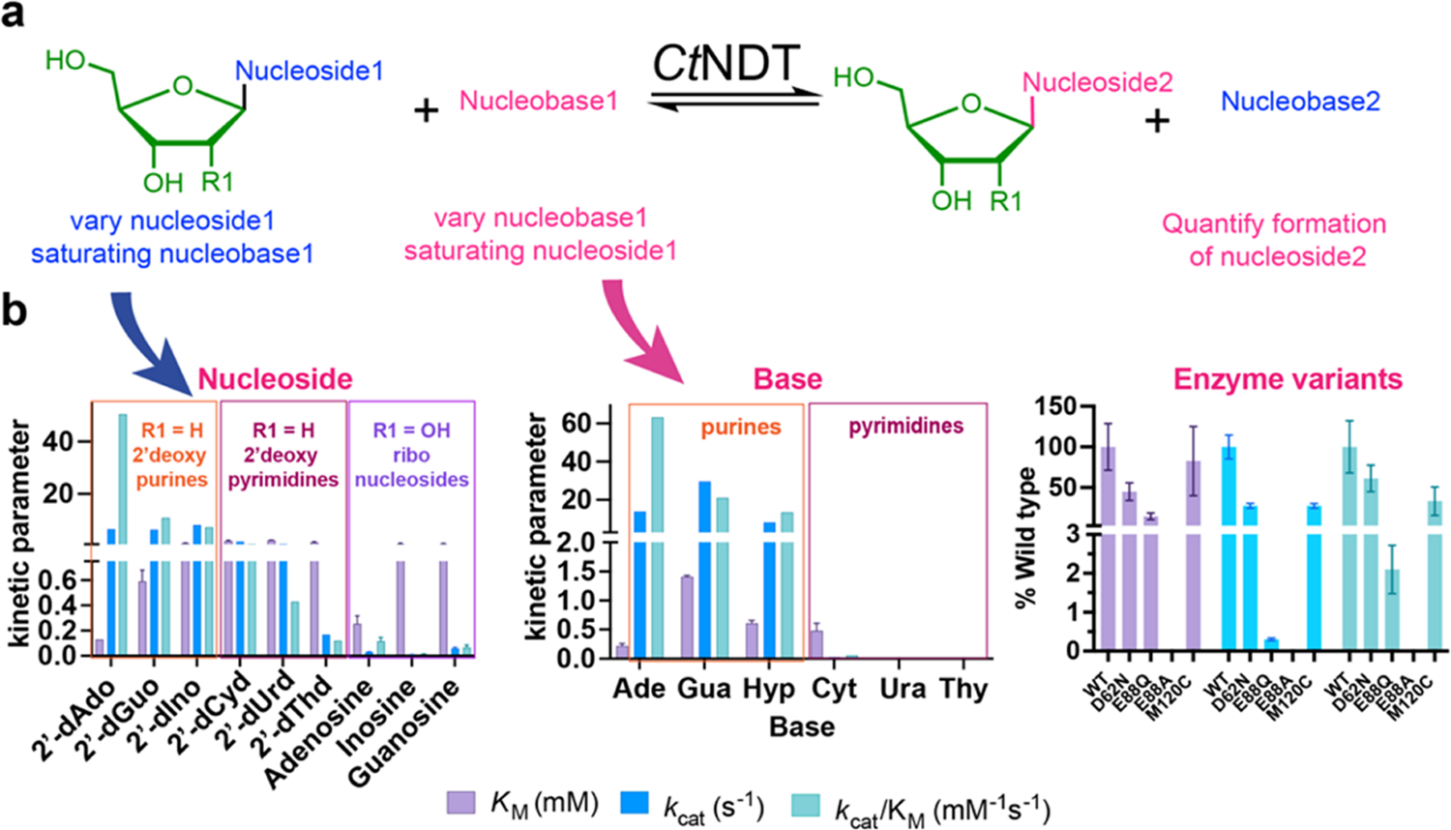
Substrate preference by C*t*NDT. (a) General scheme for the reaction catalyzed by C*t*NDT. To obtain *k*_cat_/K_M-nucleoside_, nucleoside substrates were varied and nucleobase was present at a saturating concentration, and to obtain *k*_cat_/K_M-nucleobase_, a 2′-deoxyribonucleoside substrate was present at a saturating concentration. Transglycosylation was monitored, and formation of nucleoside2 was quantified as a function of time. (b) Initial velocity studies were performed, and data were fitted to a Michaelis–Menten equation to yield kinetic parameters *K*_M_ (purple), *k*_cat_ (blue), and *k*_cat_/*K*_M_ (green) when varying (2′-deoxy)ribonucleoside (left panel) or nucleobase (middle panel). The right panel shows kinetic parameters for 2′-dAdo (varying) and Gua (fixed) for different enzyme variants as a fraction of kinetic parameters for the wild-type enzyme. Colored boxes depict 2′-deoxypurine (orange box), 2′-deoxypyrimidine (light purple box), and ribonucleoside (dark purple box) substrates. All experiments were carried out in triplicate, and data are reported as the mean and standard error of the mean. All data are shown in Figure S5. Here, the “kinetic parameter” stands for the apparent steady-state constant obtained after the Michaelis–Menten fitting (Figure S5) when using different substrates and enzyme variants.

dNDT enzymes have been previously determined to catalyze sugar-transfer reactions in a ping-pong mechanism, forming a covalent 2′-deoxyribosylated intermediate, followed by nucleobase transfer preserving stereochemistry on the anomeric carbon.^39,40^ To determine substrate specificity, we compared a series of purine and pyrimidine substrates, obtaining kinetic parameters for both nucleoside donor and nucleobase acceptor substrates (Figure 2a). Our results show that *Ct*NDT prefers purines to pyrimidines, both in terms of nucleoside sugar donors and nucleobase acceptors (Figure 2b and Table S2). For the first half-reaction, 2′-deoxyadenosine (2′-dAdo) is the preferred sugar donor (*k*_cat_/*K*_M_ = 50.5 ± 12.2 mM^–1^ s^–1^), and adenine (Ade) is the preferred sugar acceptor (*k*_cat_/*K*_M_ = 63.3 ± 12.1 mM^–1^ s^–1^) for the second half-reaction. This substrate preference pattern is in agreement with a prior study on *Ct*NDT.^21^ For purines, the higher substrate specificity is mostly *K*_M_-driven, while *k*_cat_ values for different purine substrates are similar within the experimental error (Table S2).

We tested the ribonucleosides Ado, Guo, and Ino as substrates for transglycosylation and obtained *K*_M_ values of comparable magnitude to the ones obtained with 2′-deoxyribonucleosides (*K*_M-Ado_ = 0.25 ± 0.05 mM, *K*_M-Guo_ = 1.1 ± 0.3 mM, *K*_M-Ino_ = 0.9 ± 0.2 mM). However, an over 100-fold difference in *k*_cat_ between 2′-dGuo and Guo (6.9 ± 0.7 s^–1^ at 45 °C for 2′-dGuo and 0.06 ± 0.01 s^–1^ at 65 °C for Guo) indicates that nucleobase transfer is unfavorable with ribonucleoside substrates. Prior work on dNDT from *Trypanosoma brucei*(56) demonstrated that it could use ribonucleosides as substrates for transglycosylation, but kinetic parameters for ribonucleoside substrates were not determined for this wild-type enzyme. We further discuss the acceptance of ribonucleoside substrates below, as we solved the structure of a ribosylated *Ct*NDT intermediate, providing crucial insights into substrate selection.

Based on prior reports and on our own structural data for *Ct*NDT (see below), we produced mutants *Ct*NDT_D62N_, *Ct*NDT_E88Q_, *Ct*NDT_E88A_, and *Ct*NDT_M120C_ to determine the importance of each in the catalytic mechanism. The identity of each mutant was further verified by intact protein mass spectrometry (Figure S2). By homology, E88 acts as a nucleophile, forming a glycosidic bond with the donor substrate. Our data show that *Ct*NDT_E88A_ is catalytic inactive, as expected, but *Ct*NDT_E88Q_ retains some catalytic activity, albeit the catalytic efficiency in comparison to the wild type is reduced by almost 50-fold. Any residual activity is unexpected given the crucial role proposed for E88 and might indicate that another residue in the active site vicinity can play a compensatory role in lieu of E88. This residue could be D62, as it is within hydrogen-bonding distance from the C1 of the sugar and E88 (kinetic parameters for mutants are given in Table S4and a discussion on the structure is given below). Alternatively, glutamine deamidation could occur, leading to the reversion of part of the protein into the active form with E88. To investigate this possibility, we conducted experiments incubating *Ct*NDT_E88Q_ with clofarabine, as detailed below. No covalent modifications were observed. If glutamine deamidation had taken place, it would have resulted in modifications in the enzyme fraction undergoing deamidation. Additionally, we performed a multiple turnover experiment to quantify the product formed after one turnover with *Ct*NDT_E88Q_. After a 60 s reaction (the expected time for one turnover with *Ct*NDT_E88Q_), 10 μM of the product was formed, in excellent alignment with the concentration of active sites for *Ct*NDT_E88Q_. We calculated that 0.25% deamidation would be below our limit of detection by mass spectrometry, and the same amount of the product would be observed in a single turnover experiment. Therefore, while we cannot completely rule out deamidation, we have no evidence to support it.

Computational studies proposed the formation of an oxocarbenium ion in the transition state for the reaction, and D62 is equivalent to a residue proposed to interact with the anomeric carbon in this putative oxocarbenium ion transition state.^16^*Ct*NDT_D62N_, however, retained ∼50% catalytic efficiency in comparison to WT, and it is unlikely to play such a crucial role in transition-state stabilization.

Prior work in a pyrimidine-preferring dNDT observed some large effects in the substitution of a methionine residue (M125A) by an alanine, also in proximity to the catalytic glutamate.^40^ This mutation increased *K*_M_ for substrates, so we carried out substitution on the equivalent position of *Ct*NDT_M120C_, aimed instead at decreasing *K*_M_ for substrates. This mutation had no effect on *K*_M_ but led to a mild ∼30% reduction in catalytic efficiency, demonstrating that M120 might play an indirect role in substrate positioning, as it does not interact with any substrate/analogues according to structural data discussed below.

### Transient Kinetics

**Figure 3:**
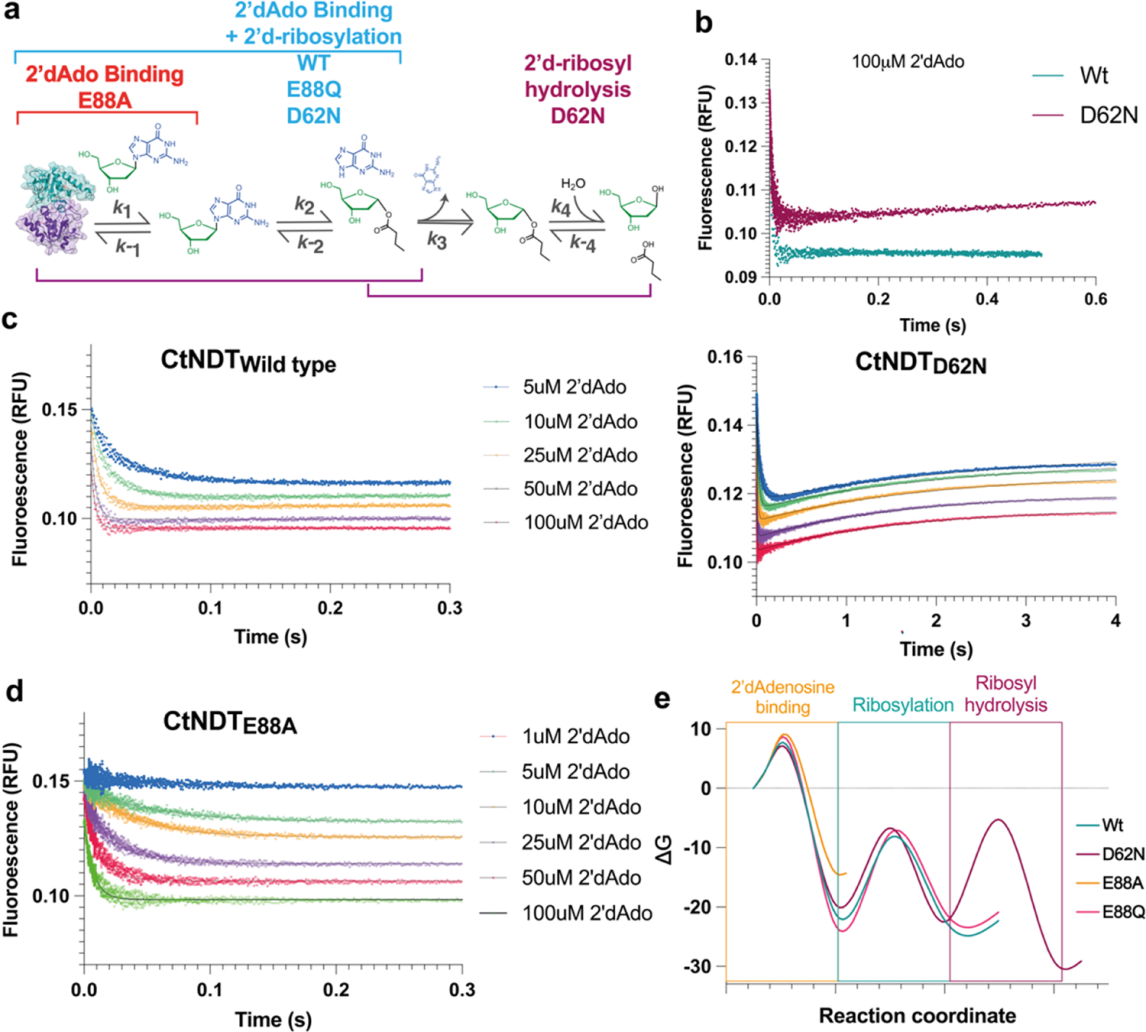
Pre-steady-state kinetics of the first half-reaction. (a) Steps and rate constants obtained with different C*t*NDT mutants. Brackets indicate the number of transients observed with each enzyme variant, using 2′-dAdo for binding or 2′-deoxyribosylation. (b–d) Raw data after mixing 0.75 mM C*t*NDT. (c) Left for wild type and right for C*t*NDT_D62N_. (d) C*t*NDT_E88A_ with increasing concentrations of 2′-dAdo as indicated. With C*t*NDT_D62N_, two transients are observed, where the initial decay is followed by a slow fluorescence increase. (b) Comparison between wild-type C*t*NDT and C*t*NDT_D62N_ showed no increase in fluorescence in the wild-type protein. (e) Rate constants obtained after fitted values (Table S5) were converted into Δ*G* (kcal mol^–1^) using eq 3, and relative barriers for each step comparing mutants are shown.

To probe individual rate constants for the mechanism depicted in Scheme 1, we performed fast kinetic experiments monitoring substrate binding and chemistry in the first half-reaction with 2′-dAdo as a substrate. We used the inactive mutant *Ct*NDT_E88A_ to isolate the substrate binding step, the less active mutants *Ct*NDT_E88Q_ and *Ct*NDT_D62N_ to determine the involvement of each of these residues in binding and/or 2′-deoxyribosylation, and a wild-type enzyme to obtain boundaries for the magnitude of the rate constant for the 2′-deoxyribosylation reaction (Figure 3a). As previously reported for another dNDT enzyme,^30^ there is a decrease in protein fluorescence upon substrate binding and/or 2′-deoxyribosylation (Figure 3b–d). Experiments were performed at 25 °C due to the presence of transients for binding and ribosylation that were too fast to capture at higher temperatures. After numerical integration of the data using KinTek Global Explorer^25,28,29^ as described in Supporting Information, values for individual rate constants were obtained (Table S5 and Figures S6–S9).

The association rate constant for 2′-deoxyadenosine binding (obtained with *Ct*NDT_E88A_) was 0.76 ± 0.01 μM^–1^ s^–1^ with a dissociation rate constant of 39.3 ± 0.7 s^–1^. *k*_cat_/*K*_M-2′Ado-25°C_ = 0.016 μM^–1^ s^–1^ (determined with the saturating concentration of nucleobase) sets a lower boundary for this bimolecular association step. Rates for 2′-deoxyribosylation, on the other hand, were very fast (532 s^–1^ for *Ct*NDT WT) and therefore did not limit *k*_cat-25°C_ (3.2 ± 0.2 s^–1^).

In our pre-steady-state experiments using mutants *Ct*NDT_D62N_, two transients with opposite amplitudes were observed, while in experiments performed with the wild-type enzyme using identical conditions, only a single transient showing fluorescence decay was observed. The second transient showed a hyperbolic dependence on ligand concentration and therefore can be attributed to an unimolecular transformation. Although this unimolecular transformation could be a conformational change in the enzyme, independent of chemistry, it could also be due to 2′-deoxyribosyl-enzyme hydrolysis (energy barrier depicted in Figure 3e). Figure S10 depicts the extent of nucleoside substrate hydrolysis by the wild type, *Ct*NDT_D62N_, and *Ct*NDT_E88Q_. More futile cycles of nucleoside hydrolysis that do not result in transglycosylation take place with *Ct*NDT_D62N_ and *Ct*NDT_E88Q_ than with the wild-type enzyme. Stopped-flow data were used with enzyme variants *Ct*NDT_E88A_ to estimate the rates of 2′-deoxyadenosine binding and release, wild-type *Ct*NDT to obtain the rate of 2′-deoxyribosylation, and *Ct*NDT_D62N_ to obtain the rate of 2′-deoxyribosyl hydrolysis. The rate of 2′-deoxyribosylation was not significantly altered in E88Q-C*t*NDT compared with the WT enzyme, in agreement with covalent intermediate formation not being rate-limiting for *k*_cat_.

The reaction catalyzed by C*t*NDT is fully reversible, and both substrates and products are equivalent, as depicted in Scheme S1. Therefore, our pre-steady-state data make clear that 2′-deoxynucleoside release is not rate-limiting, as binding experiments showed association and dissociation rate constants for 2′-deoxyadenosine 100 times faster than *k*_cat_. Because identical *k*_cat_ values were determined for 2′-deoxyadenosine and 2′-deoxyguanosine when Gua and Ade were employed as nucleobases, respectively (Table S2, steady-state kinetics of *Ct*NDT), steady-state turnover is likely to be limited by the same step when these different 2′-deoxynucleosides are used as substrates. Taking together the large viscosity effect on *k*_cat_ as expected for a completely diffusion-controlled reaction and the fast dissociation rate constant for 2′-deoxyadenosine (approximately 10x faster than *k*_cat_), we propose that a diffusional step, i.e., nucleobase1 departure, limits turnover in the *Ct*NDT-catalyzed reaction.

### Binding of Potential Transition-State Analogues

**Figure 4:**
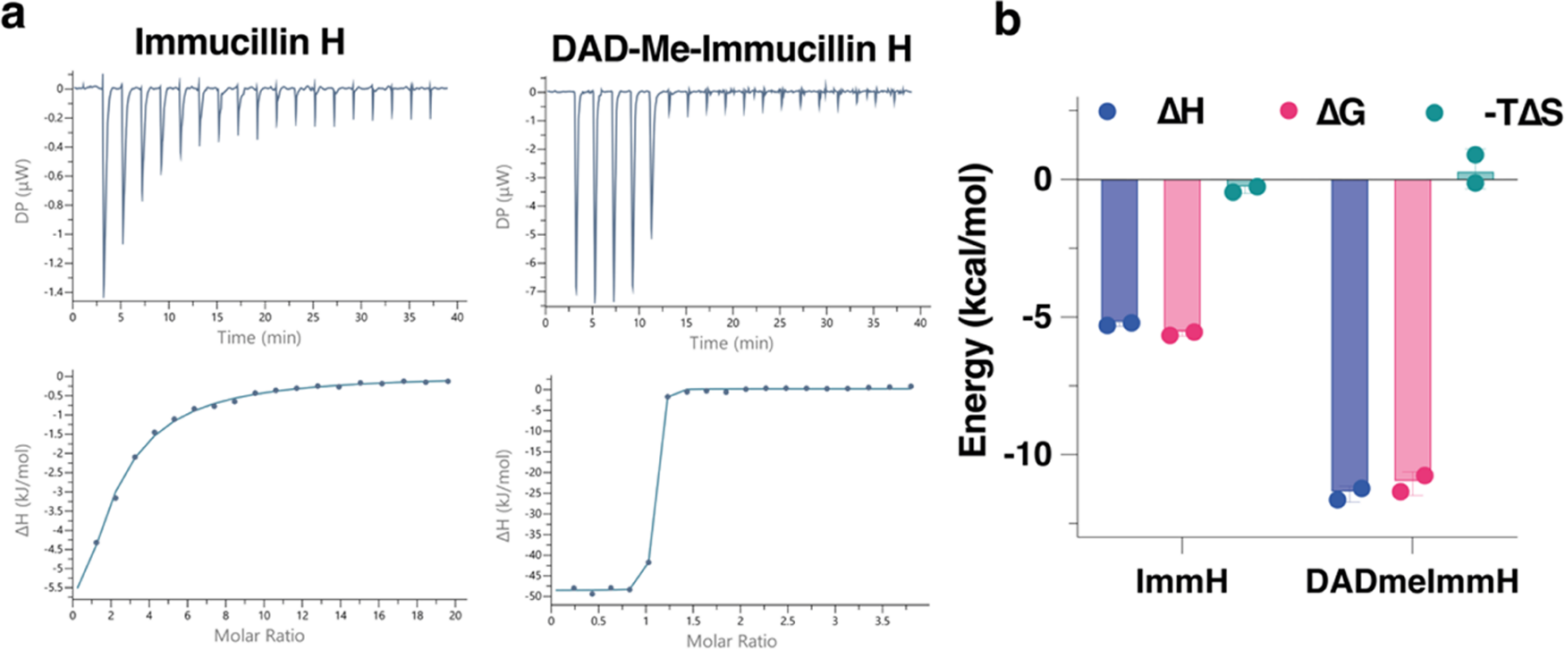
Equilibrium binding of potential transition-state analogues. Data for the equilibrium binding of analogues ImmH and DADmeH measured using isothermal titration calorimetry (ITC). (a) Wild-type C*t*NDT was present in the cell while each analogue was titrated. Raw data and fitted traces to a one-binding-site model are shown. Representative traces are shown here, and all data are available in Figures S11 and S12. (b) Fitted results for thermodynamic parameters Δ*H*, Δ*G*, and −*T*Δ*S*. The average for duplicate experiments and error spread are shown.

Schramm and co-workers developed tight-binding transition-state analogues for the reaction catalyzed by human purine nucleoside phosphorylase (PNP) and revolutionized the design of specific and potent inhibitors by studying enzymatic transition states.^57^ Such analogues have exquisite potency as drugs and also serve as important nonhydrolyzable probes to study principles of catalysis in enzymes that catalyze nucleoside transfer reactions. We compared two potential transition-state analogues for transglycosylation, immucillin-H (ImmH) and DADme–immucillin-H (DADmeH), as the distance between the bond being broken/formed in the nucleobase and sugar varies (Figure 4a).^58^ For ImmH, Δ*H* was −5.25 ± 0.07 kcal mol^–1^, −*T*Δ*S* was −0.36 ± 0.14 kcal mol^–1^, and *K*_D_ was 78.2 ± 6.6 μM, while for DADmeH, Δ*H* was −11.43 ± 0.29 kcal mol^–1^, −*T*Δ*S* was 0.38 ± 0.73 kcal mol^–1^, and *K*_D_ was 9 ± 5 nM (Table S6). Δ*G* was −5.60 ± 0.08 for ImmH and −11.05 ± 0.42 kcal mol^–1^ for DADmeH; therefore, binding is determined by an enthalpic contribution (Figure 4b).

Enzymes evolved to bind tightly to their cognate transition state and therefore to stable compounds that most closely mimic the charge distribution and geometry of that transition state.^59^ Based on Scheme 1 and considering 2′-deoxyribonucleside1 as a substrate, releasing nucleobase1 and generating a 2′-deoxyribosylated enzyme, ImmH would mimic an early transition state in which the distance between ribose C1′ and the nucleobase is ∼1.5 Å, while DADmeH would mimic a late transition state, since the presence of a methylene bridge between ribitol N1′ (which replaces C1′ in DADmeH) keeps the nucleobase at a distance of ∼3.0 Å from these groups. In the second half-reaction, the “early” and “late” definitions would have the opposite meaning, as the final product would be 2′-deoxyribonucleside2 following C–N bond formation.

Prior detailed work on the thermodynamic analysis of a series of transition-state analogues of human PNP revealed that tighter-binding TS analogues had an enthalpically driven binding process with little entropic contribution.^60^ This is in agreement with our results, as most of the Δ*G* for binding is derived from Δ*H* in both analogues studied, more pronounced for the tighter-binding late-transition-state analogue DADmeH (Figure 4b).

Furthermore, we performed kinetic binding experiments to probe whether ImmH and DADmeH binding was taking place as a single step process. Although no signal change was observed with DADmeH, ImmH binding led to a small fluorescence decrease in a single transient (Figure S13).

### Overall Structure of *Ct*NDT

The overall structure of *Ct*NDT is similar to that of *T. brucei* nucleoside 2′deoxyribosyltransferase (*Tb*NDT),^61^ as structural superposition reveals an RMSD of 0.859 Å. Size exclusion data reveal *Ct*NDT to be a tetramer in solution formed by a dimer of dimers (Figure S1), which is also corroborated by the analysis of protein interfaces with PISA.^62^ The best studied dNDT in terms of kinetics, the protein from *L. leishmanii* (*Ll*NDT),^63^ is a hexamer (a trimer of dimers), and the structural alignment of each dimer of *Ll*NDT shows an RMSD of 1.093 Å when compared to *Ct*NDT. As with other NDT enzymes, the active site of *Ct*NDT is formed by residues from two monomers, and therefore, it is only fully formed in the dimer/tetramer. Figure S14 shows a comparison between the structures of *Ct*NDT and *Ll*NDT, depicting conserved residues that play similar roles in both proteins.

### Snapshots of the Reaction Coordinate of *Ct*NDT

**Figure 5:**
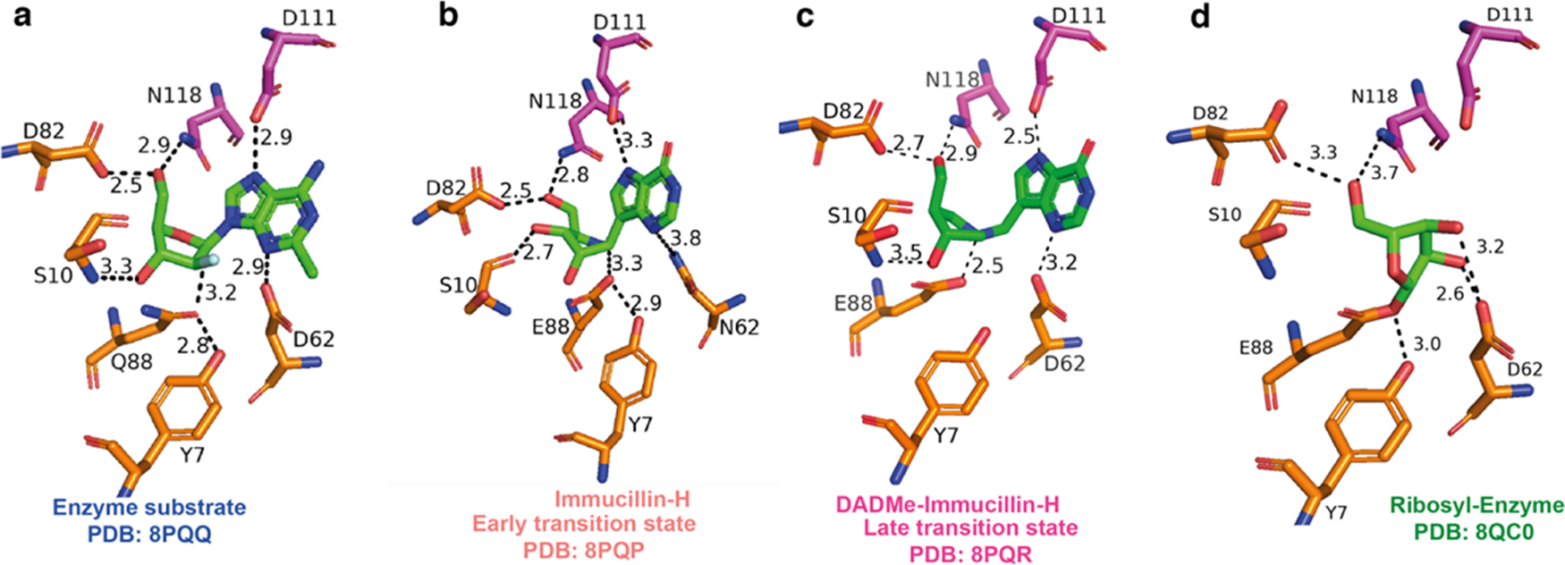
Steps in the reaction coordinate of *Ct*NDT. (a) *Ct*NDT_E88Q_ bound to clofarabine, depicting interactions of an enzyme–substrate or enzyme–product complex depending on the substrates employed. (b) *Ct*NDT_D62N_ bound to immucillin-H and (c) *Ct*NDT bound to DADme–immucillin-H, respectively, designed as “early” or “late” nucleoside-like transition-state analogues for nucleoside phosphorolysis catalyzed by PNP. (d) Ribosylated *Ct*NDT, depicting 5′-OH in close proximity to D82. An alternate conformation depicting 5′-OH facing S10 is depicted in Figure S15. Omit maps (2Fo-Fc) are depicted in Figures S16 and 6.

To further understand how *Ct*NDT binds 2′-deoxyribonucleosides and ribonucleosides and catalyzes sugar transfer, we cocrystallized *Ct*NDT in complex with adenosine (to yield a ribosylated enzyme), clofarabine (2-chloro-9-(2-deoxy-2-fluoro-β-d-arabinofuranosyl)-9*H*-purin-6-amine, a substrate/product analogue), and the transition-state analogues ImmH and DADmeH (Figure 5). Specific interactions with each ligand shed light on ligand binding and transition-state stabilization.

Enzyme–substrate complex (or enzyme–product complex, depending on the reaction direction): the complex *Ct*NDT_E88Q-clofarabine_ shows the 3′-OH of the arabinofuranosyl sugar within the hydrogen-bonding distance (3.3 Å) of S10. E88Q is 3.2 Å from C1′ in the sugar, 2.8 Å from Y7, and 3.3 Å from D62. E88Q and D62 are 3.3 Å apart, and therefore, Y7 can interact with either or both residues depending on the state in the reaction coordinate to ensure optimal positioning. Similar interaction patterns between the catalytic glutamate and the conserved tyrosine residue were observed in the dNDT from *Lactobacillus helveticus* (pdb 1S2G).^13^ Interactions with the nucleobase are mediated by hydrogen-bonding interactions with D111 and D62, and the 5′-OH is within the hydrogen-bonding distance of N118 (2.9 Å).

#### Possible Transition States for the First Half-Reaction

Structures were obtained with the two potential transition-state analogues ImmH and DADmeH. The structure of bound ImmH largely resembles that of the clofarabine-bound complex, in agreement with the relatively weak binding of ImmH to C*t*NDT, and it is therefore substrate-like. In the structure of bound DADmeH, however, E88 is 2.5 Å from N1′ in the sugar (analogous to C1′ in the substrate), 2.9 Å from Y7, and 3.5 Å from D62. D62 and Y7 are 3.4 Å apart. Stabilizing the nascent positive charge in the leaving group is D111, 2.5 Å away from Nitrogen 7 on DADmeH and 3.2 Å away from D62. The distance between D111 and N7 from DADmeH is shorter than the sum of the van der Waals radii for O and N, indicative of a very strong hydrogen bond between the two groups.^64,65^ Therefore, in the complex with DADmeH, E88 is closer to the bond that needs to be broken, D111 is closer to the nucleobase leaving group, and D62 is farther from interactions with the nucleobase when compared to an enzyme–substrate complex and also farther from the 3′-OH in comparison to the 2′-deoxyribosyl intermediate. This, coupled with the nM binding affinity of DADmeH for *Ct*NDT, points toward a late transition state for the first half-reaction in which the bond order between sugar and nucleobase is negligible. The proximity of E88 to N1′ might suggest significant nucleophile participation at the TS. Both ImmH and DADmeH were developed as mimics of the transition state of the reaction catalyzed by purine nucleoside phosphorylase (PNP).^66^ Human and bovine enzymes share 87% sequence identity, but the reactions catalyzed proceed via different transition states. While the reaction catalyzed by bovine PNP (bPNP) proceeds via an early transition state with a relatively close 1.8 Å distance between the leaving-group nitrogen and the anomeric carbon, human PNP (hPNP) ribosidic bond cleavage occurs via a fully dissociated purine leaving group with a fully developed oxocarbenium ion, with a distance of 3.0 Å between the two groups.^67^

#### Covalent Enzyme–Ribosyl Intermediate

After nucleobase1 departure, the enzyme is 2′-deoxyribosylated until hydrolysis or the second half-reaction occurs. Although dNDT enzymes are selective for 2′-deoxyribonucleosides, molecular detail underlying this preference is lacking. We therefore determined the structure of a ribosylated enzyme intermediate by cocrystallization of wild-type *Ct*NDT in the presence of Ado. Residues D82 and N118 are in close proximity to the 5′-OH of the intermediate (3.3 and 3.5 Å, respectively). D62 is in very close proximity (2.6 Å) from 2′-OH and to 3′-OH (3.2 Å), and the proximity to 2′-OH (absent in 2′-deoxyribonucleosides) can lead to less optimal positioning of this intermediate for nucleophilic attack by the incoming nucleobase 2.

An important observation is the positioning of Y7, likely polarizing the –COO^–^ side chain of E88. Y7 does not directly interact with ligands, nor is it at a close enough distance to clash with a potential 2′-OH in the ribonucleoside ligand. One possibility is that Y7 restricts the movement of catalytic E88, but this optimal positioning for 2′-deoxyribonucleosides results in clashes between D62 and the 2′-OH of ribonucleoside substrates, leading to a reduced catalytic efficiency with these substrates.

The electron density surrounding the ribosylated intermediate is poorly defined, which could indicate that the intermediate is sampling multiple conformations (Figure S14 depicts one of such conformations in which the 5′-OH is turned away from D82) or that there is partial density for adenosine, which is not defined well enough to be accurately modeled. We therefore confirmed the presence of covalent adduct formation via an orthogonal method. Prior work with the *Lh*NDT^13^ trapped a 2′-deoxy-2′-fluoro-ribosyl intermediate upon incubation with a 2′-deoxy-2′-fluoro-β-d-arabinofuranosyladenine substrate, and it was hypothesized that 2′-fluoro-containing substrates undergo slower hydrolysis in comparison to compounds lacking fluorine on position 2′. We report a structure of a 2-difluoro-2-deoxy-ribosylated C*t*NDT after cocrystallization with Gemcitabine for comparison with the ribosylated enzyme. Figure S17 shows a comparison between ribosylated and 2-difluoro-2-deoxy-ribosylated C*t*NDT. The main difference is in the conformation sampled by the 3′-OH of the 2-difluoro-2-deoxy-ribosylated intermediate, positioned closer to S10 than to D62. After the ligand was added to the protein, refinement (with Phenix Refine) produced three monomers with a covalently modified E88 and one with a covalent modification on D62. We do not have additional biochemical evidence for the formation of a covalent intermediate with D62, and because this could be an artifact, we have not discussed this in detail.

To further confirm that the ribosylated intermediate was trapped in solution as well as in crystallo, wild-type *Ct*NDT was incubated with the substrate clofarabine. After incubation, the intact mass of wild-type *Ct*NDT increased by 135 Da, in agreement with what would be expected if a 2′-deoxy-2′-fluoro-ribosyl intermediate was present (Figure S18a–c).

**Figure 6:**
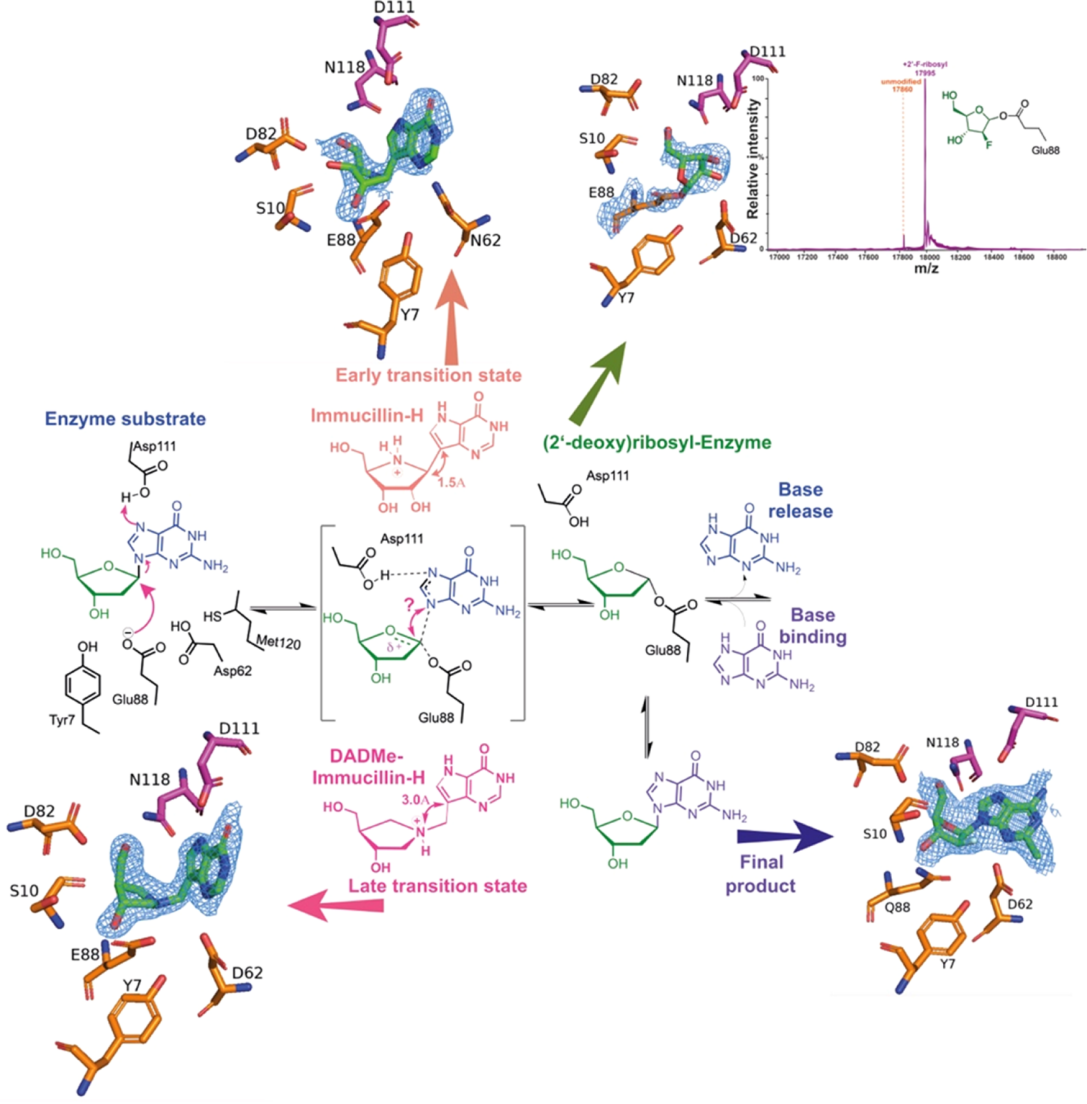
Snapshots of the reaction coordinate for the first half-reaction catalyzed by *Ct*NDT. Proposed mechanism for the *Ct*NDT-catalyzed reaction, combining data from protein mass spectrometry and X-ray crystallography. Electron density for ligands is depicted as −2Fo-Fc at 2σ contour (maps prepared using likelihood-weighted laps on Phenix, and image prepared using Pymol). Distances shown for ImmH and DADmeH are from ref (58).

This is the first structural characterization of a dNDT enzyme covalently modified by a ribonucleoside, enabling further engineering efforts to improve catalytic efficiency toward ribonucleoside analogues (Figure 6).

## Conclusions and Implications for dNDT Enzymes

Our work provides a detailed mechanistic understanding of a thermophilic nucleoside transferase enzyme. Combining enzymology and biophysical techniques, we present a unique insight into the reaction coordinate for (2′-deoxy)nucleoside transfer, including interactions important for substrate selection and catalysis. Our data support a late transition state for the first half-reaction and determine that nucleobase1 release is rate-limiting during steady-state turnover.

In recent years, several have hypothesized that nonzero activation heat capacity might explain the presence of curved Arrhenius plots in enzyme catalysis.^50^ This model has been proposed to account for temperature optima in enzymes that are not associated with the initiation of protein unfolding.^38^ In the *Ct*NDT-catalyzed reaction, the significant contribution of negative heat capacity to a reaction is limited by a diffusional step, highlighting the importance of determining rate-limiting steps when evaluating the contribution of heat capacity to enzyme-catalyzed reactions, as well as considering changes in rate-limiting steps in the temperature range under investigation.^37^

*Ct*NDT demonstrated promiscuity in terms of substrate scope and reactions catalyzed—(2′-deoxy)ribosylated hydrolysis or (2′-deoxy)ribosyl transfer. Enzyme promiscuity has been considered an ancestral trait, often lost in modern enzymes under prolonged selection for efficient substrate turnover.^68,69^ This knowledge has been applied to generate enzymes with expanded function,^70^ as well as in ancestral enzyme reconstruction efforts. Our work is a crucial step to understand dNDT enzymes and informs future enzyme evolution^55^ and engineering^71^ efforts on this enzyme class aimed at producing novel nucleosides.

## Supporting Information Available

Additional methods for protein production, enzymatic assays, mass spectrometry, HPLC conditions, and DSF experiments; raw data for assays; melting temperature determination using differential scanning fluorimetry; hydrolysis of nucleoside substrates uncoupled from base transfer; primers for cloning and mutagenesis; pre-steady-state parameters globally fitted using KinTek Global Explorer; and crystallization conditions and crystallization table for pdb entries 8PQP, 8PQQ, 8PQR, 8PQS, 8PQT, 8QC0, and 8RH3 (PDF)
