# Supplementary material for: Snapshots of the Reaction Coordinate of a Thermophilic 2′-Deoxyribonucleoside/ribonucleoside Transferase
Source: ACS Catal. 2024 Feb 13;14(5):3090–102. doi: 10.1021/acscatal.3c06260 (PMC10913048; doi:10.1021/acscatal.3c06260)
Supplement: Supplementary file 1 — cs3c06260_si_001.pdf [file cs3c06260_si_001.pdf]

Supporting information

Snapshots of the reaction coordinate of a thermophilic 2′-deoxyribonucleoside/ribonucleoside transferase

Peijun Tang[a], Christopher J. Harding[a], Alison L. Dickson[b], Rafael G. da Silva[a], David J. Harrison[b], Clarissa Melo Czekster[a]

[a]School of Biology, Biomedical Sciences Research Complex, University of St Andrews, St Andrews, Fife KY16 9ST, United Kingdom; [b]School of Medicine, University of St Andrews, North Haugh, St Andrews, KY16 9TF, UK

*To whom correspondence may be addressed: cmc27@st-andrews.ac.uk, phone: +44 (0)1334 463792

Content:

Supporting methods 4
Size exclusion chromatography 4
Protein mass spectrometry 4
Standard enzyme kinetics of CtNDT with (2´deoxy)nucleoside substrates 5
HPLC conditions for reaction monitoring 5
Differential Scanning Fluorimetry for protein melting temperature determination 6
Viscosity effect on k_{cat} 6
Derivation of rate equations for CtNDT and discussion of rate limiting steps for kcat 7
Input Data 7
Rate Equation 7
Scheme S1: First and second half reactions for CtNDT 9
Supporting Figures 10
Figure S1 CtNDT WT purification and size exclusion chromatography 10
Figure S2 Intact mass spectrum of CtNDT and its mutants. 11
Figure S3: Melting temperature determination using differential scanning fluorimetry. 13
Figure S4: Data supporting Figure 2. 15
Figure S5: Representative HPLC chromatograms of time courses and Michaelis-Menten kinetics on substrates utilized by CtNDT WT and its mutants. 16
Stopped flow data analysis 23
.... 23
Figure S6: 2´-deoxyadenosine binding to CtNDTE88A. 24
Figure S8: 2´-deoxyadenosine binding to $CtNDT_{E88Q}$ 25
Figure S9: 2´deoxyadenosine binding to CtNDTD62N 26
Figure S10: Hydrolysis of nucleoside substrates uncoupled from base transfer: 28
Figure S11 CtNDT (Wt) binding to Immucillin-H 29
Figure S12 CtNDT (Wt) binding to DAD-Me-Immucillin-H 29
Figure S13: Immucillin-H binding to CtNDT and $CtNDT_{E88Q}$. 30
Figure S14: Comparison between substrate binding pocket and active site of CtNDT 31
Figure S15: Comparison between alternate conformations of the ribosylated-enzyme intermediate 32

Figure S16: 2Fo-Fc maps at 2s for complex structures reported here. 34
Figure S17: Structure of 2-difluoro-2'-deoxy-ribosylated CtNDT. 34
Figure S18: Intact protein mass spectra of CtNDT and its mutants in the absence and presence of Clofarabine 35
Table S1 Primers for cloning and mutagenesis 36
Table S2 Steady-state kinetics of CtNDT 36
Table S3 Steady-state kinetics of CtNDT WT with 2´-deoxyribonucloesides and ribonucleosides 37
Table S4 Steady-state kinetics of CtNDT and mutants with 2´-dGuo and Adenine 37
Table S5 Pre-steady state parameters globally fitted using Kintek Global Explorer 38
Table S6 Summary of fitted ITC parameters 40
Table S7 Crystallization conditions 41
Table S8 Crystallographic Data Table 42

Supporting methods

Abbreviations: 2´-deoxyadenosine (2´-dAdo), 2´-deoxyguanosine (2´-dGuo), 2´-deoxyinosine (2´-dIno), 2´-dUridine (2´-dUrd), 2´-dCytidine (2´-dCyd), 2´-dThymidine (2´-dThd), Adenine (Ade), Guanine (Gua), Hypoxanthine (Hyp), Cytidine (Cyt), Adenosine (Ado), Guanosine (Guo), Inosine (Ino), Uracil (Ura), liquid-chromatography-mass spectrometry (LC-MS), polyethylene glycol (PEG), Immucillin H (ImmH), DADme-Immucillin-H (DADmeH).

Protein sequence

The coding sequence for protein expression (derived from the sequence available at Uniprot, accession K9TVX3) was as below, where the region in bold is removed after cleavage with TEV protease:

MHHHHHHDYDIPTTENLYFQGMKRKIIYLASPYGFSQQQKTLLLPPIVRALEALGIEV
WEPFARNNQIDFSQADWAYRVAQADLQDVKNCDGIFAVVNGTPPDEGVMVELGM
AIALNKAIFLFRDDFRRCSDNERYPLNLMLFAGLPEIGWENYYYTSVDEIQSHDKAL
YKWLTGM

Size exclusion chromatography

100 ml of CtNDT (1 mg/mL) was injected onto a Superdex™ 75 Increase 10/300 GL to estimate the protein quaternary structure by relative mass. Buffer was 50 mM MES, 250 mM NaCl, pH 6.5 with a flow rate of 0.2 mL/min. The elution peak from the SEC column was around 10.03 mL, corresponding to a mass of ~ 62 kDa calculated with a calibration curve (Figure. S2). For the calibration curve, a Gel Filtration Calibration Kit LMW (Biorad) was used, including aprotinin (bovine lung), ribonuclease A (bovine pancreas), Carbonic anhydrase (bovine erythrocytes), Ovalbumin (hen egg), Conalbumin (chicken egg white) and blue dextran 2000.

Protein mass spectrometry

20 μM enzyme alone or after overnight incubation with 1mM Clofarabine by end-over-end rotation were used. Protein samples were analysed at the University of St Andrews mass spectrometry and proteomics facility. For analysis, 20ul of sample at 1:20 dilution were injected onto a Waters MassPrep micro column 2mmx5mm on a Waters Xevo LC-MS system optimised for protein analysis. A short gradient elution was used to desalt and then elute the protein as follows:

**Time (mins)**	**%A (98% water with 0.1% Formic acid and 2% acetonitrile)**	**%B (100% acetonitrile with 0.1% Formic acid)**
**0.200**	98.0	2.0
**0.50**	98.0	2.0
**3.80**	2.0	98.0
**4.50**	2.0	98.0
**4.60**	100.0	0
**5.00**	100.0	0

The MS was operated in ESI+ and scanned from 500 – 2500m/z with lock mass of LeuEnk. The protein spectrum elution at 3 minutes was combined and the raw data processes to mass using MaxEnt algorithm at 0.1 resolution using peak width of half height of 0.4Da.

Standard enzyme kinetics of CtNDT with (2´deoxy)nucleoside substrates

A standard assay contained CtNDT, a 2´deoxynucleoside and a base (50 µl reaction volume) in a mixed buffer solution (final 30 mM CHES, MES and HEPES, pH 8.5) incubated at 45°C for 5, 10 and 15 minutes. At these times, an aliquot from the reaction mixture was removed and quenched with 200 µl of 10 M Urea, centrifuged at 24,000g for 10 minutes. 100 µl of quenched mixture were taken and placed into 96-well round bottom microplate (Agilent Technologies) and 10 µl of each sample were injected into the HPLC column (using a Shimadzu Prominence HPLC and the method described below). All the experiments were done in duplicate. Data were fitted using a Michaelis-Menten equation in Graphpad Prism and the kinetic parameters were plotted with substrates or mutants. Concentrations used for assays are as follows:

**Enzyme and concentration**	**Nucleoside1**	**Base1**	**Product used for quantification:**
**D62N (5 nM)**	10 µM - 2500 µM 2´- dGuo	10 mM Ade	2´- dAdo
**E88Q (0.1 µM)**	10 µM - 2500 µM 2´- dGuo	10 mM Ade	2´- dAdo
**M120C (0.4 µM)**	10 µM - 2500 µM 2´- dGuo	10 mM Ade	2´- dAdo
**WT (5 nM)**	10 µM - 2500 µM 2´- dAdo	10 mM Hyp	2´- dIno
**WT (5 nM)**	10 µM - 1000 µM 2´- dGuo	10 mM Ade	2´- dAdo
**WT (5 nM)**	10 µM - 2500 µM 2´- dIno	10 mM Ade	2´- dAdo
**WT (40 nM)**	10 µM - 2500 µM 2´- dCytosine	10 mM Ade	2´- dAdo
**WT (25 nM)**	10 µM - 2500 µM 2´- dUrd	10 mM Ade	2´- dAdo
**WT (50 nM)**	10 µM - 5000 µM 2´- dThy	10 mM Ade	2´- dAdo
**WT (1 µM)**	10 µM 2´- dAdo	10 µM – 1 mM Cytidine	2´- dCytosine
**WT (1 nM)**	10 mM 2´- dAdo	10 µM – 2.5 mM Gua/Hyp	2´- dGuo/2´- dIno respectively
**WT (1 nM)**	10 mM 2´- dGuo	10 µM – 1 mM Gua	2´- dAdo

Product formation was quantified using calibration curves for each (2´-deoxy)ribonucleoside product and converting the integrated area values into concentration.

HPLC conditions for reaction monitoring

A 2.5 mm, 50 mm X 4.6 mm HSS T3 column (Waters™) was used with buffer A (10 mM trimethylammonium acetate, pH 7) and buffer B (ACN + 0.1% TFA). A gradient elution was used to separate the compounds: from 0-10 mins, 99% to 85% buffer A and 1% to 15% buffer B, from 10-15 mins, 85% to 0% buffer A and 15% to 100% buffer B. The oven temperature was set at 40°C and the absorbance was set at 260 nm. The column was equilibrated with buffer A: buffer B (1:99) for at least 15 mins before each injection. Retention times for reference standard compounds were obtained by running each standard (Figure S3, top).

Differential Scanning Fluorimetry for protein melting temperature determination

Enzyme was prepared the day before the assays and the concentration of the sample was calculated as $1000/MW_{protein\ monomer}$ mM. The concentration of sypro orange (5000X stock in DMSO) was prepared as 10X in the protein purification buffer. Enzyme (5 ml), the test buffer (20 ml) and the sypro orange working dye solution (25 ml) were mixed in 0.2 ml non-skirted 96-well PCR plates. Data were acquired using a Stratagene Mx3005p instrument, under a temperature gradient from 25°C - 95°C in 1°C increments for a total of 1.5h. For each condition, triplicate experiments were carried out. Data were analysed by fitting to a Boltzmann equation in Prism 9.0 to obtain melting temperatures in different conditions.

Viscosity effect on k_{cat}

To obtain information about rate limiting steps in the temperature range in which deviation from linear Arrhenius behaviour was observed, we determined k_{cat} at increasing concentrations of glycerol. Reactions were carried out with 20nM wild type CtNDT and 0, 15 and 24% glycerol at 35 degrees or 0, 18 and 27% glycerol at 55 degrees and saturating concentration of substrates (1mM 2´- dGuo and 10 mM Ade). Formation of 2´- dAdo was quantified. Test assays with 2mM 2´- dGuo showed both 1mM and 2mM 2´- dGuo were saturating at the maximum % glycerol used (24% at 35 degrees and 27% at 55 degrees). Data are shown on Figure S3.

Derivation of rate equations for CtNDT and discussion of rate limiting steps for kcat

According to Scheme 1, rate equations describing kinetic parameters k_{cat}, k_{cat}/K_M-nucleoside and k_{cat}/K_M-nucleobase:

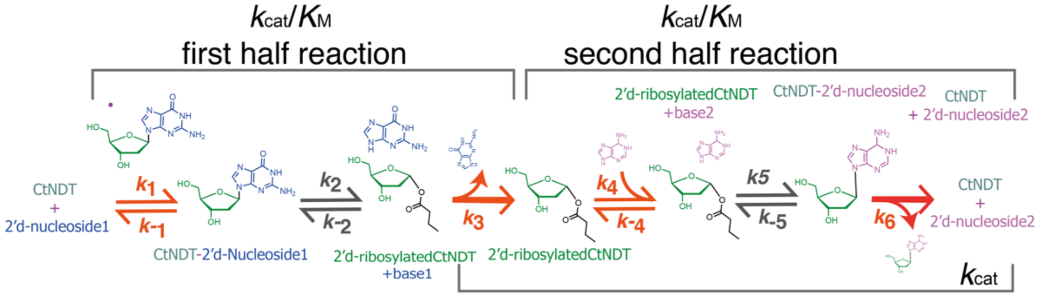

We used the BioKine rate equation generator (http://www.biokin.com/tools/king-altman/submit.html) with the following script, where N1 is nucleoside1, B2 is nucleobase2 (reactants), N2 is nucleoside2 and B1 is nucleobase1 (products):

Input Data

```
; 'Ordered Ping Pong' mechanism
;;
; Segel, I.H. (1975) "Enzyme Kinetics"
; John Wiley, New York, p. 649:

[reaction]
N1 + B2 <==> N2 + B1

[modifiers]
  ; no inhibitors or activators:
  ; this section can be absent

[mechanism]
E + N1 <==> EN1
EN1 <==> ERB1
ERB1 <==> ER + B1
ER + B2 <==> ERB2
ERB2 <==> EN2
EN2 <==> E + N2

[end]
```

v = -dS/dt = dP/dt

Using the King-Altman method[1] and the output provided by BioKine, we used the following expressions for numerator and denominator:

Rate Equation

$v = \boldsymbol{N}/\boldsymbol{D} = d[N2]/dt = + k_6\,[EN2]' - k_{-6}\,[N2]\,[E]'$

$N = \{(- k_{-1}k_{-2}k_{-3}k_{-4}k_{-5}k_{-6})[N_2][B_1] + (k_1k_2k_3k_4k_5k_6)[N_1][B_2]\}$

D = d_1 [N2][B1] + d_2 [B2][N2] + d_3 [N1][B1] + d_4 [N1][B2] + d_5 [B1] + d_6 [N2] + d_7 [B2] + d_8 [N1]

v= N/D = $\{(-k_{-1}k_{-2}k_{-3}k_{-4}k_{-5}k_{-6})[N_2][B_1] + (k_1k_2k_3k_4k_5k_6)[N_1][B_2]\}/\{(k_{-2}k_{-3}k_{-4}k_{-5}k_{-6} + k_{-1}k_{-3}k_{-4}k_{-5}k_{-6} + k_2k_{-3}k_{-4}k_{-5}k_{-6} + k_{-1}k_{-2}k_{-3}k_{-5}k_{-6} + k_{-1}k_{-2}k_{-3}k_{-4}k_{-6} + k_{-1}k_{-2}k_{-3}k_5k_{-6})[N_2][B_1] + (k_{-1}k_{-2}k_4k_{-5}k_{-6} + k_{-1}k_3k_4k_{-5}k_{-6} + k_2k_3k_4k_{-5}k_{-6} + k_{-1}k_{-2}k_4k_5k_{-6} + k_{-1}k_3k_4k_5k_{-6} + k_2k_3k_4k_5k_{-6})[B_2][N_2] + (k_1k_{-2}k_{-3}k_{-4}k_6 + k_1k_{-2}k_{-3}k_{-4}k_{-5} + k_1k_{-2}k_{-3}k_5k_6 + k_1k_2k_{-3}k_{-4}k_6 + k_1k_2k_{-3}k_{-4}k_{-5} + k_1k_2k_{-3}k_5k_6)[N_1][B_1] + (k_1k_{-2}k_4k_5k_6 + k_1k_3k_4k_5k_6 + k_1k_2k_4k_5k_6 + k_1k_2k_3k_4k_6 + k_1k_2k_3k_4k_{-5} + k_1k_2k_3k_4k_5)[N_1][B_2] + (k_{-1}k_{-2}k_{-3}k_{-4}k_6 + k_{-1}k_{-2}k_{-3}k_{-4}k_{-5} + k_{-1}k_{-2}k_{-3}k_5k_6)[B_1] + (k_{-1}k_{-2}k_{-4}k_{-5}k_{-6} + k_{-1}k_3k_{-4}k_{-5}k_{-6} + k_2k_3k_{-4}k_{-5}k_{-6})[N_2] + (k_{-1}k_{-2}k_4k_5k_6 + k_{-1}k_3k_4k_5k_6 + k_2k_3\,k_4k_5k_6)[B_2] + (k_1k_2k_3k_{-4}k_6 + k_1k_2k_3k_{-4}k_{-5} + k_1k_2k_3k_5k_6)[N_1]\}$

Under initial rate, [B1] and [N2]~0 so all terms with those also disappear:
D = d_4 [N1][B2] + d_7 [B2] + d_8 [N1]

v= N/D = $\{(k_1k_2k_3k_4k_5k_6)[N_1][B_2]\}/\{(k_1k_{-2}k_4k_5k_6 + k_1k_3k_4k_5k_6 + k_1k_2k_4k_5k_6 + k_1k_2k_3k_4k_6 + k_1k_2k_3k_4k_{-5} + k_1k_2k_3k_4k_5)[N_1][B_2] + (k_{-1}k_{-2}k_4k_5k_6 + k_{-1}k_3k_4k_5k_6 + k_2k_3k_4k_5k_6)[B_2] + (k_1k_2k_3k_{-4}k_6 + k_1k_2k_3k_{-4}k_{-5} + k_1k_2k_3k_5k_6)[N_1]\}$

divide all by $[N_1][B_2]$
v= N/D = $\{(k_1k_2k_3k_4k_5k_6)\}/\{(k_1k_{-2}k_4k_5k_6 + k_1k_3k_4k_5k_6 + k_1k_2k_4k_5k_6 + k_1k_2k_3k_4k_6 + k_1k_2k_3k_4k_{-5} + k_1k_2k_3k_4k_5) + (k_{-1}k_{-2}k_4k_5k_6 + k_{-1}k_3k_4k_5k_6 + k_2k_3k_4k_5k_6)/[N_1] + (k_1k_2k_3k_{-4}k_6 + k_1k_2k_3k_{-4}k_{-5} + k_1k_2k_3k_5k_6)/[B_2]\}$

both [N1] and [B2] are very large so terms can be removed:
v= $\{(k_1k_2k_3k_4k_5k_6)\}/\{(k_1k_{-2}k_4k_5k_6 + k_1k_3k_4k_5k_6 + k_1k_2k_4k_5k_6 + k_1k_2k_3k_4k_6 + k_1k_2k_3k_4k_{-5} + k_1k_2k_3k_4k_5)\}$

v= $\{(k_1k_4k_2k_3k_5k_6)\}/\{(k_1k_4k_5k_6)(k_{-2} + k_3 + k_2) + (k_1k_4k_2k_3)(k_6 + k_{-5} + k_5)\}$

v/Et = kcat = $\{(k_2k_3k_5k_6)\}/\{(k_5k_6)(k_{-2} + k_3 + k_2) + (k_2k_3)(k_6 + k_{-5} + k_5)\}$ => this is the rate equation for a full turnover resulting in nucleobase transfer

---

for kcat/KM-N1, [N1]<<<KM, [B2]>>>Km
v/Et = kcat/KM[N1]
Under initial rate, [B1] and [N2]~0 so all terms with those also disappear. [N1] is small so this term also disappears:
v = $\{(k_1k_2k_3k_4k_5k_6)[N_1][B_2]\}/\{(k_1k_{-2}k_4k_5k_6 + k_1k_3k_4k_5k_6 + k_1k_2k_4k_5k_6 + k_1k_2k_3k_4k_6 + k_1k_2k_3k_4k_{-5} + k_1k_2k_3k_4k_5)[N_1][B_2] + (k_{-1}k_{-2}k_4k_5k_6 + k_{-1}k_3k_4k_5k_6 + k_2k_3k_4k_5k_6)[B_2]\}$
divide all by B2:
v/Et= kcat/KM[N1]= $\{(k_1k_2k_3k_4k_5k_6)[N_1]\}/\{(k_{-1}k_{-2}k_4k_5k_6 + k_{-1}k_3k_4k_5k_6 + k_2k_3k_4k_5k_6)\}$,
simplify to remove $k_4k_5k_6$
v/Et = kcat/KM[N1] = $\{(k_1k_2k_3)[N_1]\}/\{(k_{-1}k_{-2} + k_{-1}k_3 + k_2k_3)\}$
v/Et = kcat/KM-N1 = $\{(k_1k_2k_3)\}/\{(k_{-1}k_{-2} + k_{-1}k_3 + k_2k_3)\}$ => this is the kcat/KM for Nucleoside1

Under initial rate, [B1] and [N2]~0 so all terms with those also disappear.

$v = N/D = \{(k_1k_2k_3k_4k_5k_6)[N_1][B_2]\}/\{(k_1k_{-2}k_4k_5k_6 + k_1k_3k_4k_5k_6 + k_1k_2k_4k_5k_6 + k_1k_2k_3k_4k_6 + k_1k_2k_3k_4k_{-5} + k_1k_2k_3k_4k_5)[N_1][B_2] + (k_{-1}k_{-2}k_4k_5k_6 + k_{-1}k_3k_4k_5k_6 + k_2k_3k_4k_5k_6)[B_2] + (k_1k_2k_3k_{-4}k_6 + k_1k_2k_3k_{-4}k_{-5} + k_1k_2k_3k_5k_6)[N_1]\}$

for kcat/KM B2, [B2]<<<KM, [N1]>>>Km

$v/Et = kcat/KM[B2]$

Under initial rate, [B1] and [N2]~0 so all terms with those also disappear. [B2] is small so this term also disappears:

$v/Et = kcat/KM[B2] = \{(k_1k_2k_3k_4k_5k_6)[B_2]\}/\{(k_1k_2k_3k_{-4}k_6 + k_1k_2k_3k_{-4}k_{-5} + k_1k_2k_3k_5k_6)\}$, simplify

$v/Et = kcat/KM = \{(k_4k_5k_6)\}/\{k_{-4}k_6 + k_{-4}k_{-5} + k_5k_6)\}$, => this is the kcat/KM for nucleobase2

Rate limiting steps:

The reaction catalysed by *Ct*NDT is fully reversible, and both substrates and products are (or can be) identical, as depicted below where forward rate constants lead to nucleoside bond breaking and ribosylated protein while reverse rate constants lead to nucleoside formation. Therefore, our pre-steady-state data makes clear that 2′-deoxynucleoside release is not rate limiting, as binding experiments showed association and dissociation rate constants for 2′-deoxyadenosine 100 times faster than k_{cat}. Because identical k_{cat} values were determined for 2′-deoxyadenosine and 2′-deoxyguanosine when Gua or Ade were employed as nucleobases, respectively (Table S2 Steady-state kinetics of *Ct*NDT), steady-state turnover is likely to be limited by the same step when these different 2′-deoxynucleosides are used as substrates, which argues against different values for k_{on} and k_{off} for different purine nucleosides. Therefore, one can consider the *Ct*NDT reaction coordinate (and more generally for dNDTs) is a "mirror image", meaning the binding of the first substrate is akin kinetically to the release of the last product as they are both nucleosides. Scheme S1 exemplifies this point:

Scheme S1: First and second half reactions for *Ct*NDT

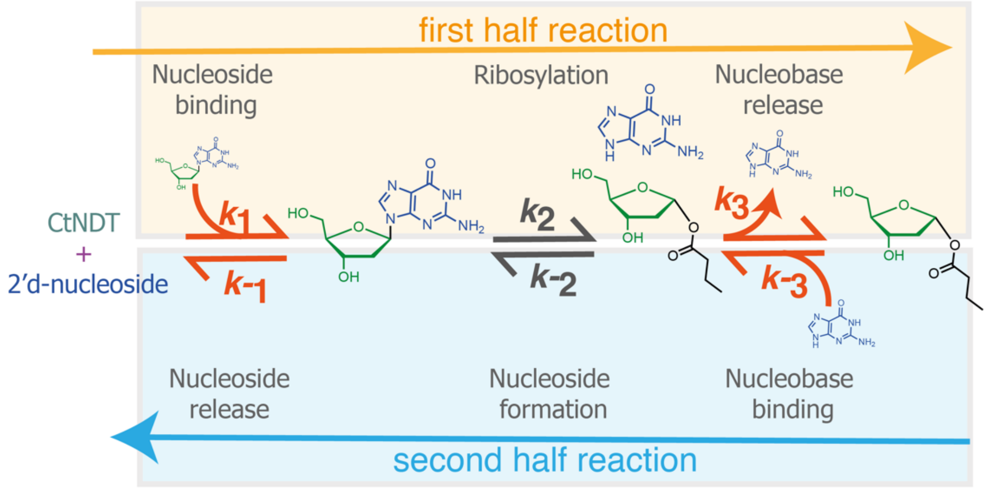

Therefore, given 1) the maximum viscosity effect of 1.0 observed which points towards a diffusional step limiting the reaction; 2) the fast rate constants for $k1$ and k_{-1} 100x faster than k_{cat}; and 3) viscosity experiment performed with 10 mM nucleobase, more than 10x over K_M, resulting in a fast observed rate of nucleobase association; the rate imiting step for CtNDT is likely nucleobase1 release (k_3 in the scheme above or k_6 in Scheme 1 shown in the main paper).

Supporting Figures

Standards	Elution Vol (Ve)	Vol 0 (Dextran Blue)	Vol t *	Ve/Vo	Mr (kDa)	Mr (Dalton)	Lg (Dalton)	Kav
Conalbumin	9.598	8.545	23.56	1.123229959	75	75000	4.875061263	0.07012987
Ovalbumin	10.59	8.545	23.56	1.23932124	43	43000	4.633468456	0.136197136
Carbonic anhydrase	13.77	8.545	23.56	1.611468695	29	29000	4.462397998	0.347985348
Ribonuclease A	15.74	8.545	23.56	1.842012873	13.7	13700	4.136720567	0.479187479
Aprotinin	19.14	8.545	23.56	2.239906378	6.5	6500	3.812913357	0.705627706

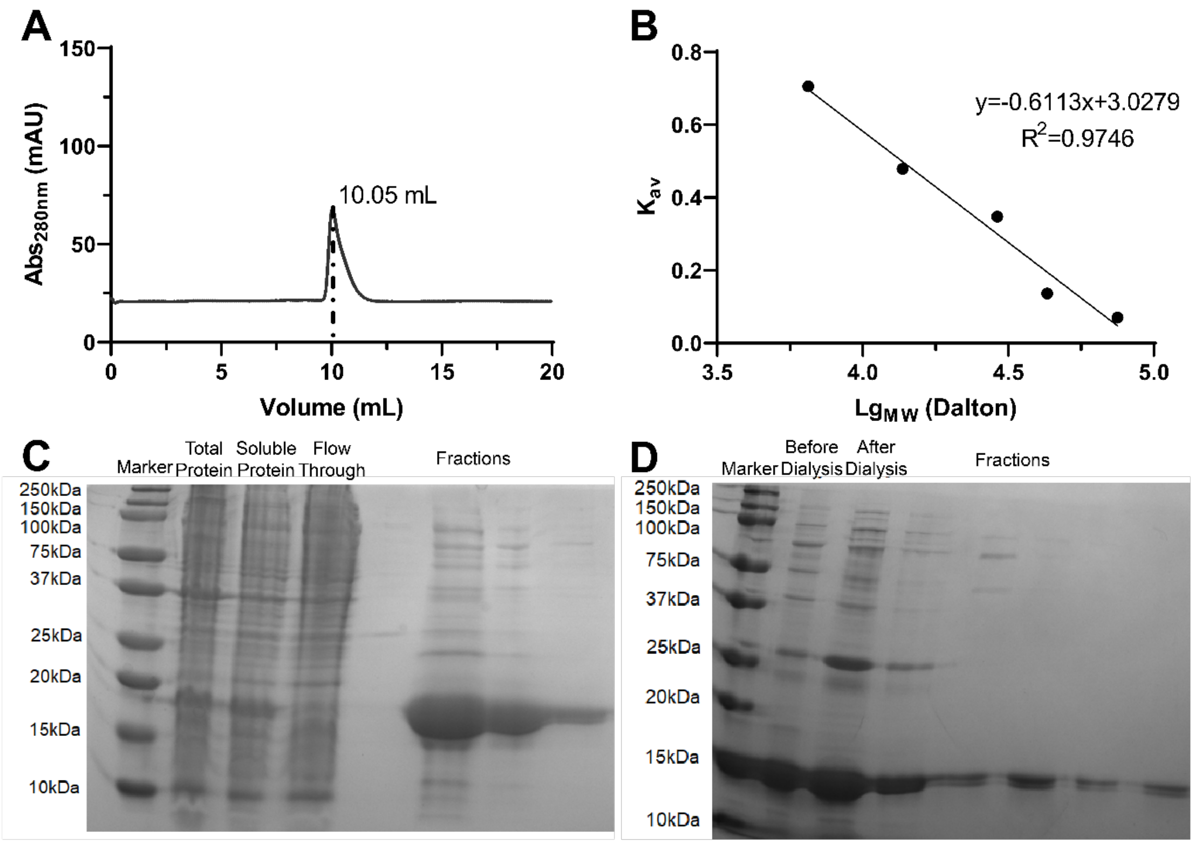

Figure S1 CtNDT WT purification and size exclusion chromatography.
(a) Chromatogram from gel filtration column. (b) Calibration curve of the Superdex™ 75 Increase 10/300 GL. 4-15% Mini-Protean™ TGX Stain-Free™ protein gels analysis after 1st nickel (c) and TEV protease cleavage 2nd nickel (d) fractions eluted from size exclusion chromatography.

The MW of the protein is estimated as follows:

$$MW=e^{\frac{K_{av}-3.0279}{-2.655}}$$

$$K_{av} = \frac{V_e - V_o}{V_t - V_o}$$

Where: MW in Da; V_e = Elution volume; V_o = Void volume; V_t = Total bed volume

The elution peak from the SEC column was around 10.03 mL, corresponding to a mass of ~ 62 kDa calculated with the calibration curve on panel B.

Figure S2 Intact mass spectrum of CtNDT and its mutants.

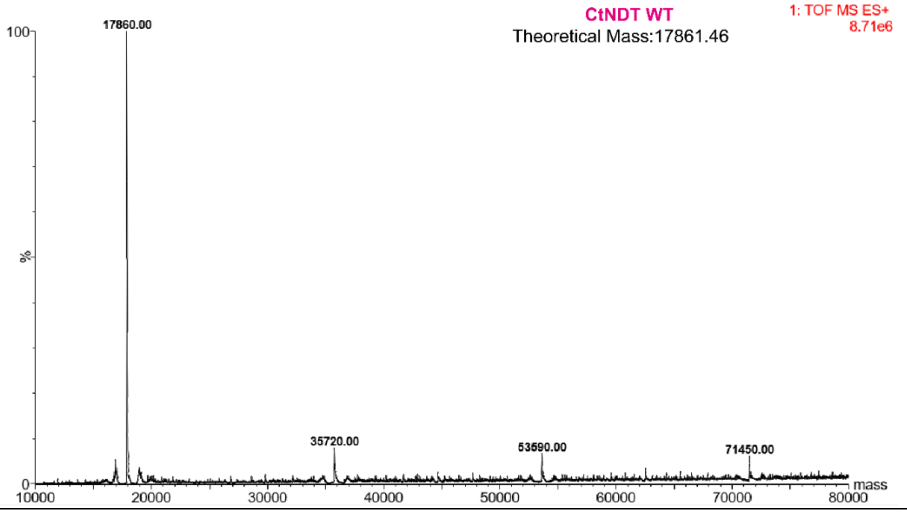

Figure S2a: Intact protein mass spectrum of wild type CtNDT. Expected MW= 17861.4, observed 17860.0.

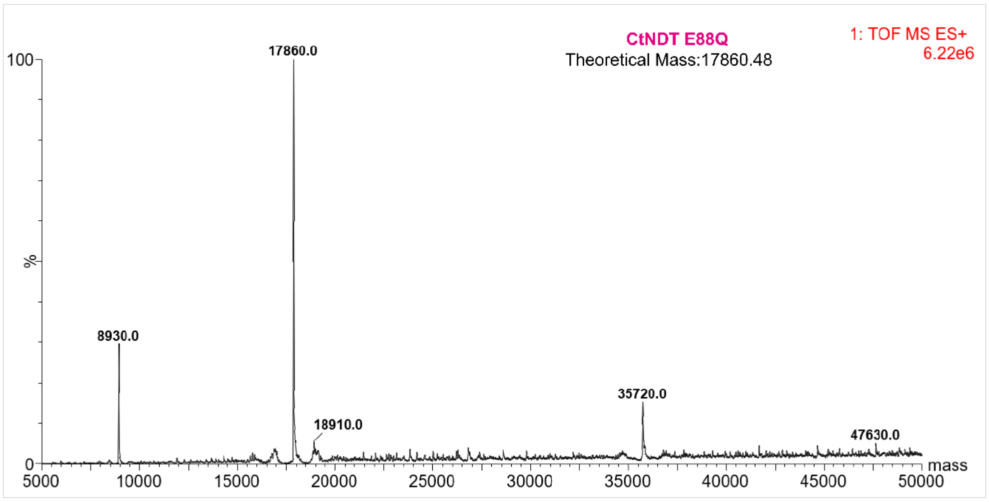

Figure S2b: Intact protein mass spectrum of CtNDT$_{E88Q}$. Expected MW= 17860.5, observed 17860.0.

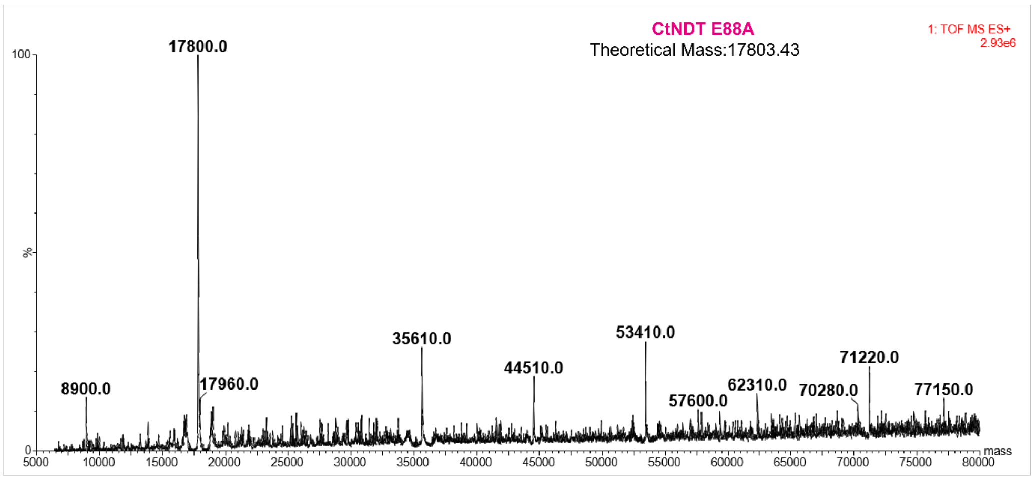

Figure S2c: Intact protein mass spectrum of CtNDT$_{E88A}$. Expected MW= 17803.4, observed 17800.0.

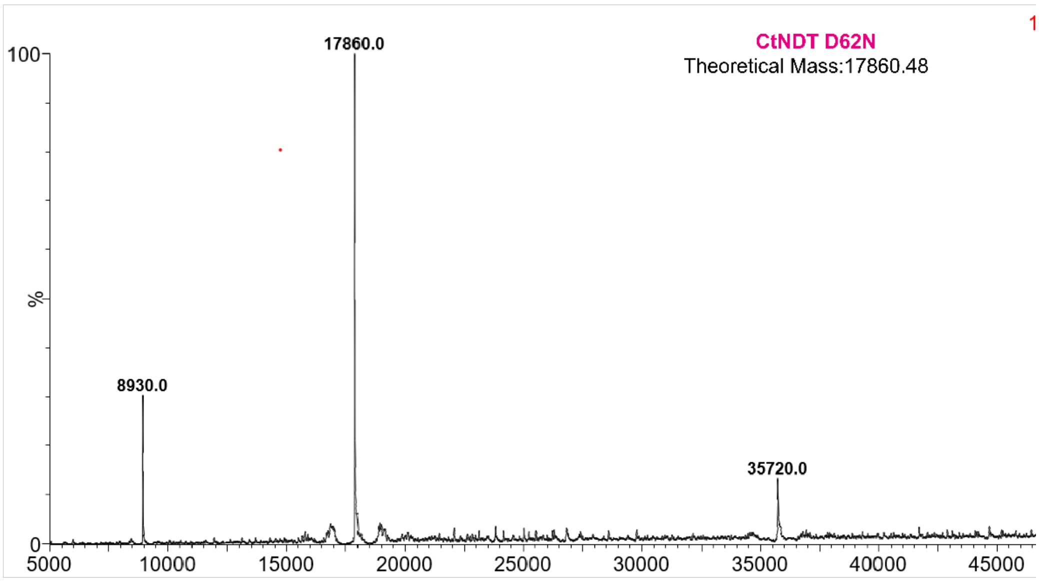

Figure S2d: Intact protein mass spectrum of $CtNDT_{D62N}$. Expected MW= 17860.4, observed 17860.0.

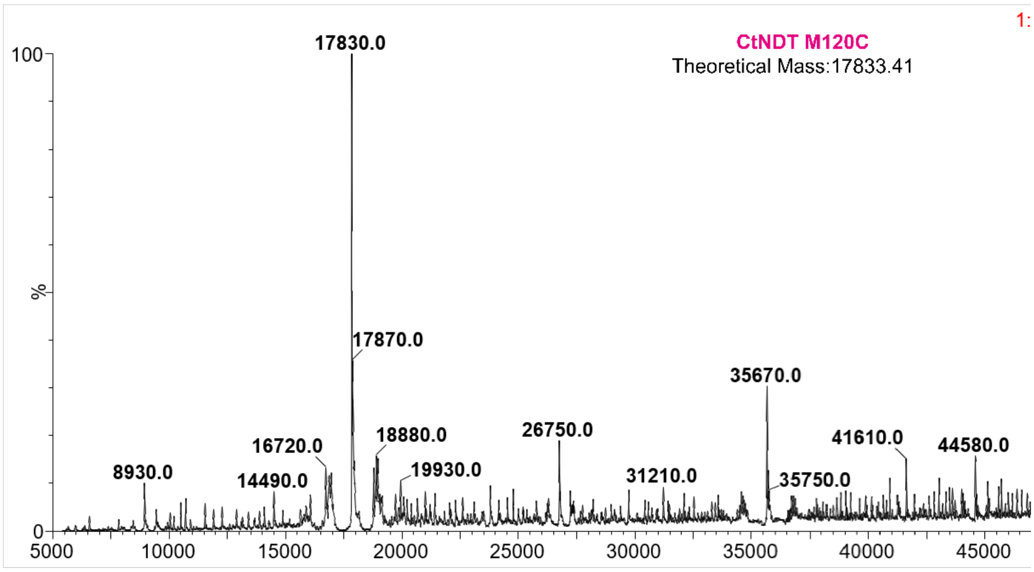

Figure S2e: Intact protein mass spectrum of $CtNDT_{M120C}$. Expected MW= 18783.4, observed 17830.0.

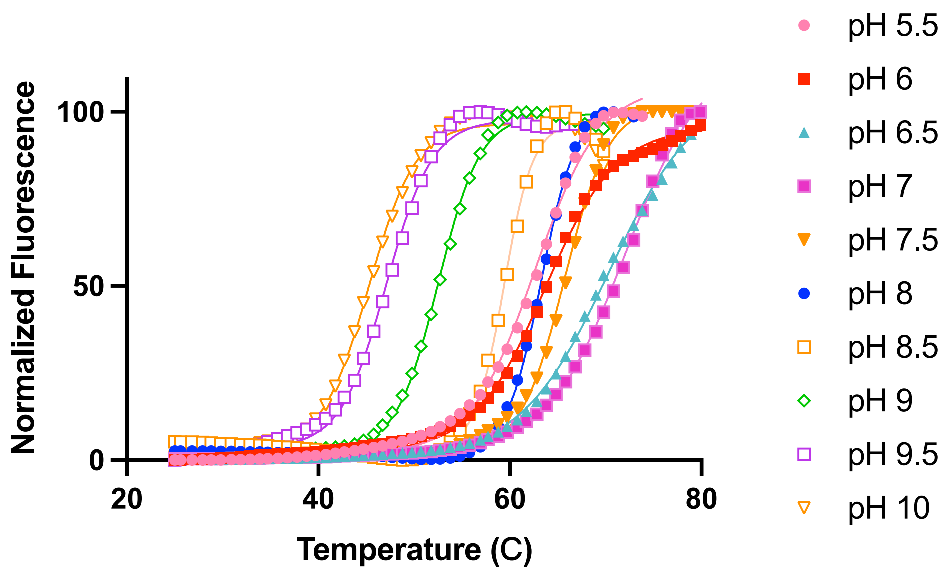

pH	T_M	error
pH 5.5	62.6	0.1
pH 6	63.6	0.1
pH 6.5	70.3	0.5
pH 7	72.0	0.6
pH 7.5	65.7	0.1
pH 8	63.3	0.1
pH 8.5	59.4	0.1
pH 9	52.5	0.1
pH 9.5	47.1	0.2
pH 10	45.3	0.1

Figure S3: Melting temperature determination using differential scanning fluorimetry. Data were analysed by fitting to a Boltzmann equation in Prism 9.0 to obtain melting temperatures in different conditions (solid lines), shown in the table. All data were acquired in triplicate and shown here as the mean value (symbols) for clarity.

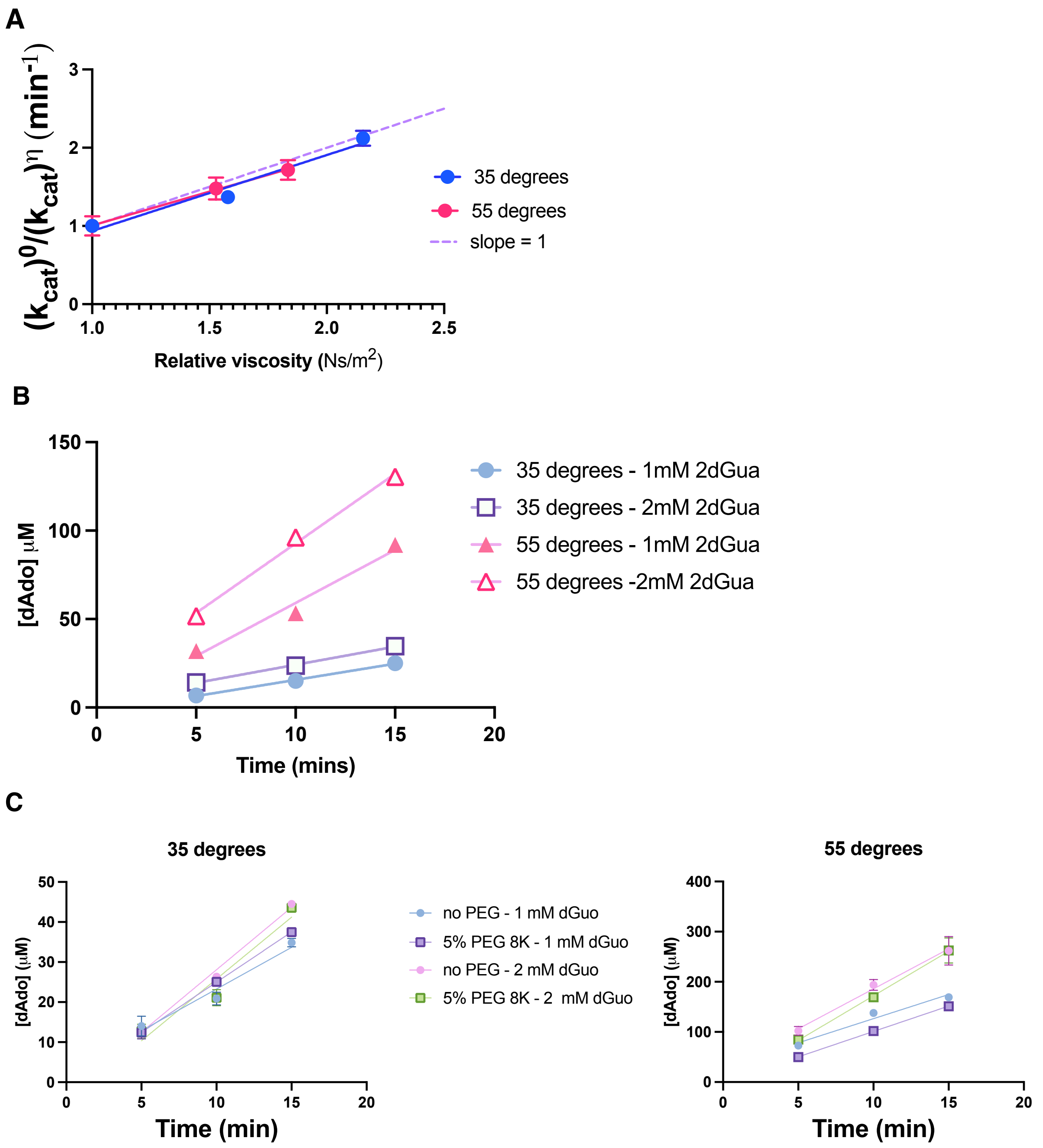

Viscosity effect	35 °C	55 °C
	1.0 ± 0.2	0.9 ± 0.1

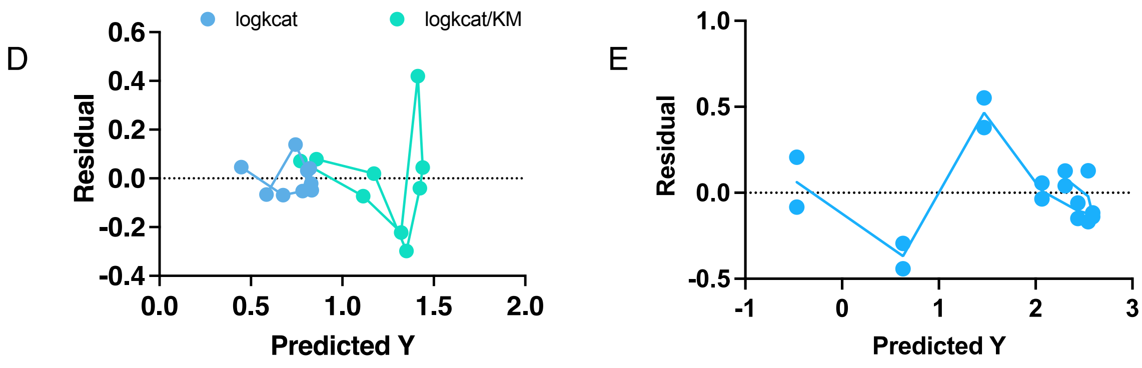

Figure S4: Data supporting Figure 2. Solvent viscosity effect on kcat using glycerol as a microviscogen: A) replot of relative rate in function of relative viscosity, using 20 nM CtNDT, 1 mM 2′dGuo and 10 mM adenine. Experiments were performed in duplicate and data show as mean and standard error of the mean. A hypothetical curve with slope = 1 is shown (purple dotted line) as a reference for a reaction fully limited by diffusional steps. B) control experiment comparing reaction rates with 1mM and 2mM 2′dGuo, demonstrating no change in slopes within experimental error, and therefore that the rate observed on (A) corresponds to k_{cat}. C) control experiment with PEG 8K (no effect), which would be acting as a macroviscogen. D) Residual plots for fits on Figure 2a and E) Figure 2b.

Figure S5: Representative HPLC chromatograms of time courses and Michaelis-Menten kinetics on substrates utilized by CtNDT WT and its mutants.

Compound	Retention time	Compound	Retention time
2´-deoxyadenosine (2´-dAdo)	8.2 min	Adenine (Ade)	2.4 min
2´-deoxyguanosine (2´-dGuo)	3.8 min	Guanine (Gua)	1.4 min
2´-deoxyinosine (2´-dIno)	2.5 min	Hypoxanthine (Hyp)	1.4 min
2´-dUridine (2´-dUrd)	2.2 min	Uracil (Ura)	0.7 min
2´-dCytidine (2´-dCyd)	1.7 min	Cytidine (Cyt)	0.9 min
2´-dThymidine (2´-dThd)	5.9 min	Thymine (Thy)	2.2 min
		Adenosine (Ado)	8.1 min
		Guanosine (Guo)	2.5 min
		Inosine (Ino)	2.8 min

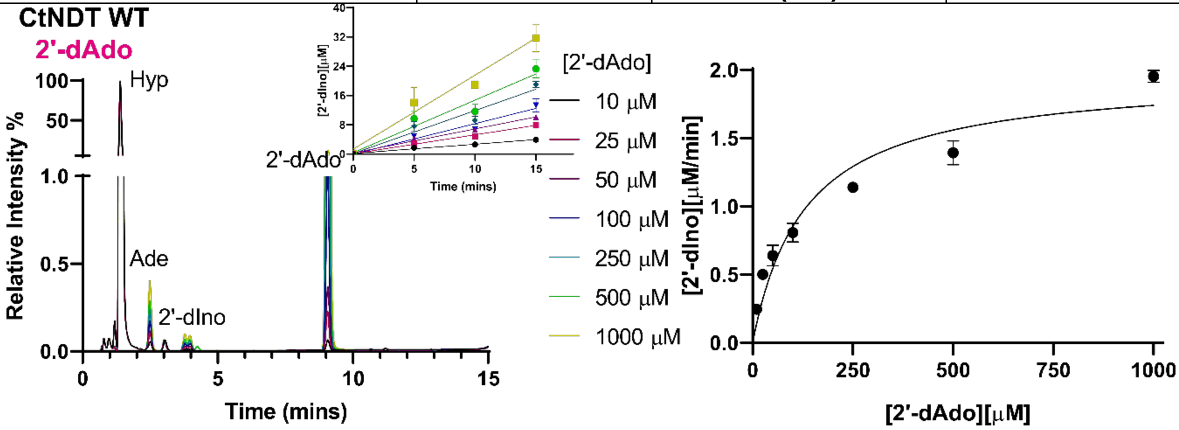

Figure S5a: Initial velocity assays with wild type CtNDT, while varying 2´-dAdo with fixed Hyp. Reactions were performed in duplicate according to the methods reported herein. Left panel depicts raw HPLC data with peaks for compounds detected labelled. Areas were integrated and converted into concentration product formed per unit of time for the Michaelis Menten plot shown on the right.

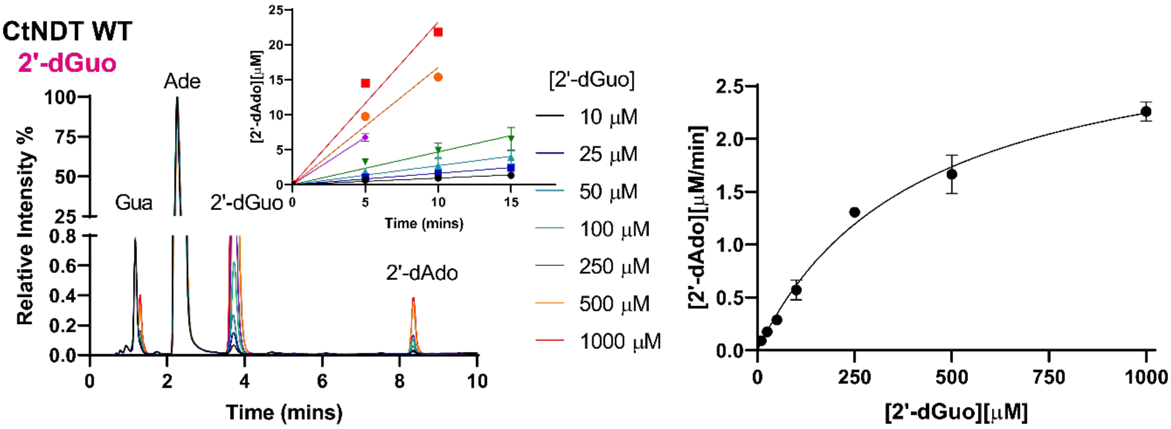

Figure S5b: Initial velocity assays with wild type CtNDT, while varying 2´-dGuo with fixed Ade. Reactions were performed in duplicate according to the methods reported herein. Left panel depicts raw HPLC data with peaks for compounds detected labelled. Areas were integrated and converted into concentration product formed per unit of time for the Michaelis Menten plot shown on the right.

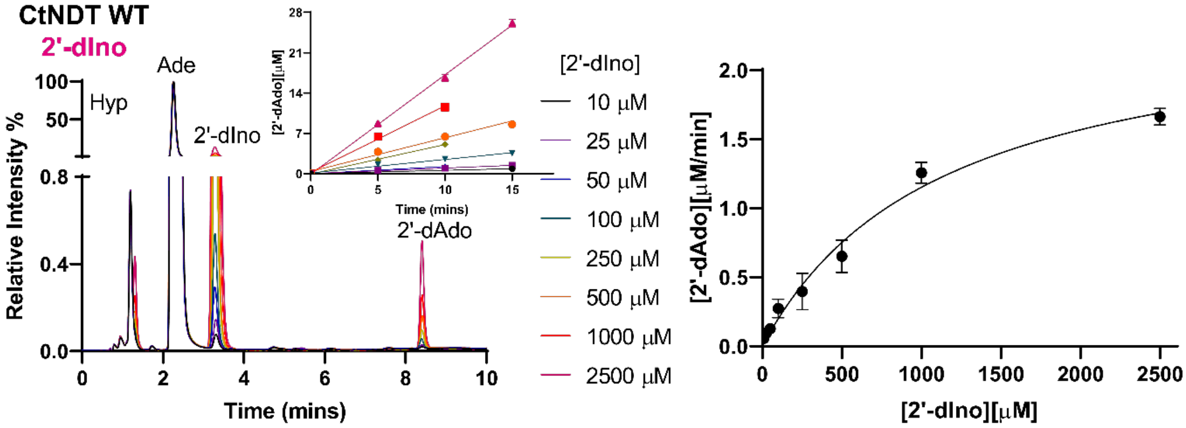

Figure S5c: Initial velocity assays with wild type CtNDT, while varying 2´-dIno with fixed Ade. Reactions were performed in duplicate according to the methods reported herein. Left panel depicts raw HPLC data with peaks for compounds detected labelled. Areas were integrated and converted into concentration product formed per unit of time for the Michaelis Menten plot shown on the right.

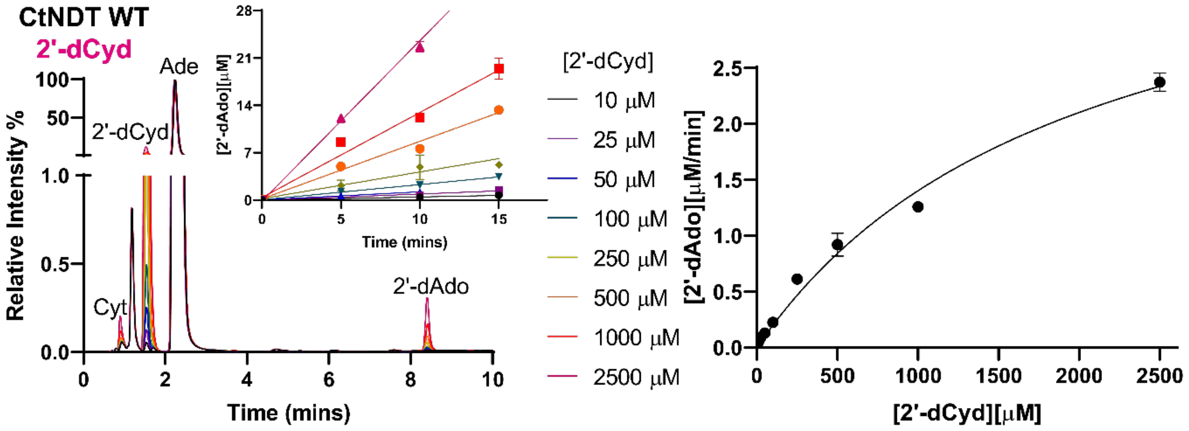

Figure S5d: Initial velocity assays with wild type CtNDT, while varying 2´-dCyd with fixed Ade. Reactions were performed in duplicate according to the methods reported herein. Left panel depicts raw HPLC data with peaks for compounds detected labelled. Areas were integrated and converted into concentration product formed per unit of time for the Michaelis Menten plot shown on the right.

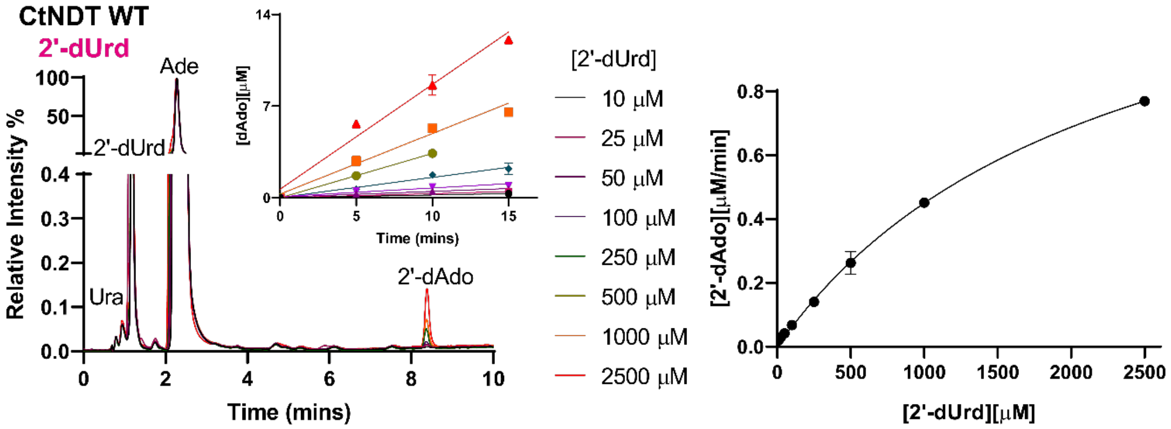

Figure S5e: Initial velocity assays with wild type CtNDT, while varying 2´-dUrd with fixed Ade. Reactions were performed in duplicate according to the methods reported herein. Left panel depicts raw HPLC data with peaks for compounds detected labelled. Areas were

integrated and converted into concentration product formed per unit of time for the Michaelis Menten plot shown on the right.

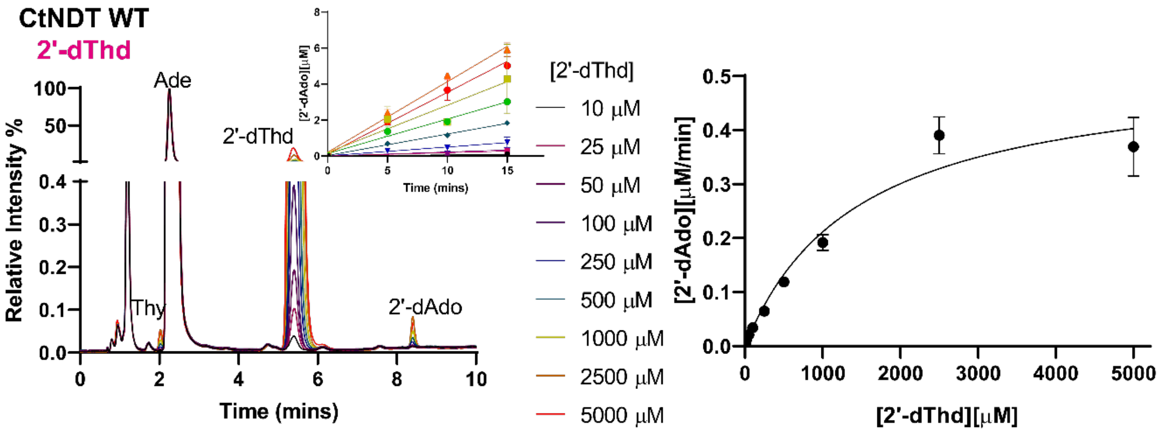

Figure S5f: Initial velocity assays with wild type CtNDT, while varying 2´-dThd with fixed Ade. Reactions were performed in duplicate according to the methods reported herein. Left panel depicts raw HPLC data with peaks for compounds detected labelled. Areas were integrated and converted into concentration product formed per unit of time for the Michaelis Menten plot shown on the right.

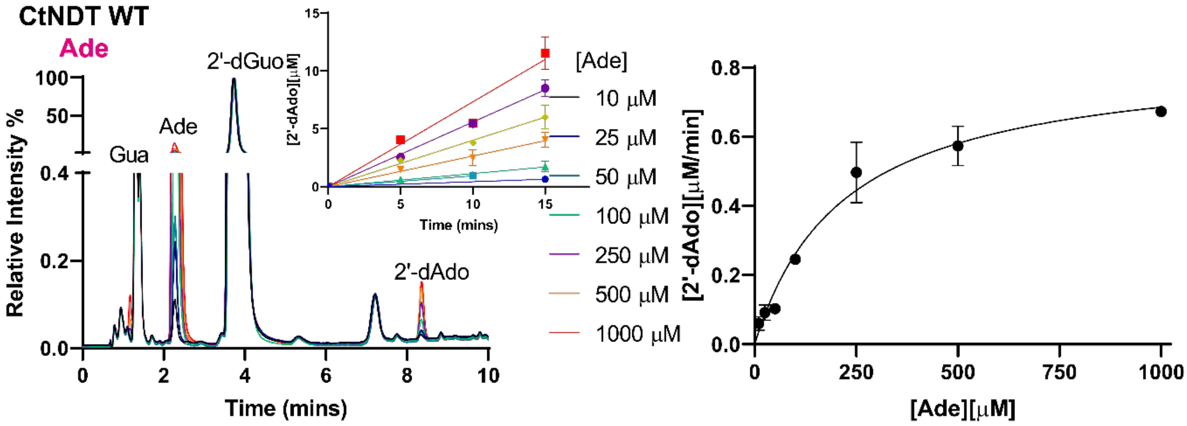

Figure S5g: Initial velocity assays with wild type CtNDT, while varying Ade with fixed 2´-dGuo. Reactions were performed in duplicate according to the methods reported herein. Left panel depicts raw HPLC data with peaks for compounds detected labelled. Areas were integrated and converted into concentration product formed per unit of time for the Michaelis Menten plot shown on the right.

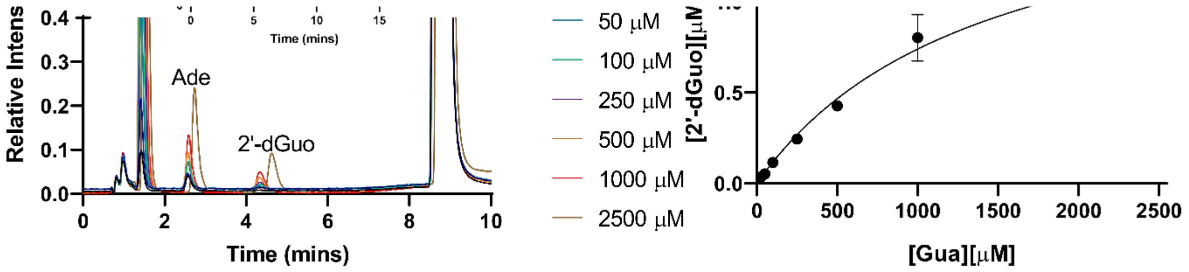

Figure S5h: Initial velocity assays with wild type CtNDT, while varying Gua with fixed 2´-dAdo. Reactions were performed in duplicate according to the methods reported herein. Left panel

depicts raw HPLC data with peaks for compounds detected labelled. Areas were integrated and converted into concentration product formed per unit of time for the Michaelis Menten plot shown on the right.

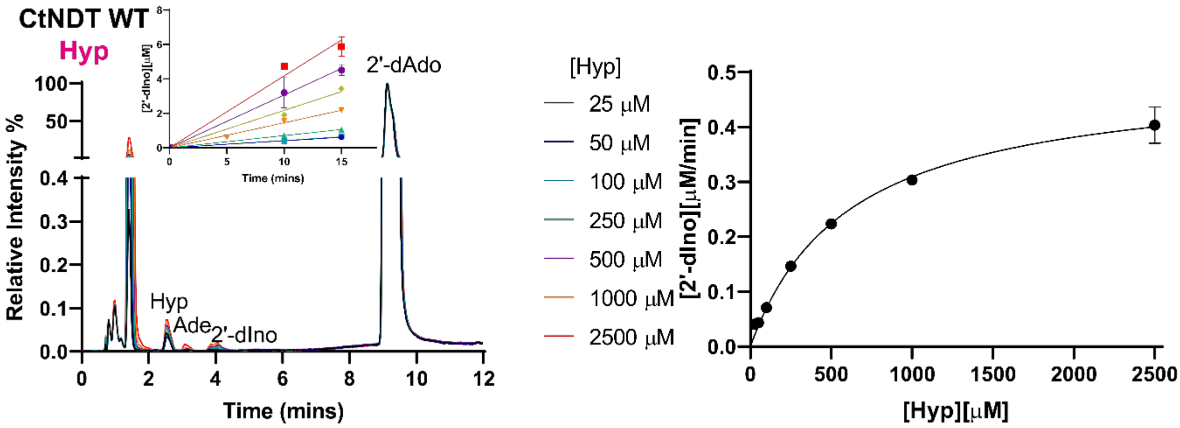

Figure S5i: Initial velocity assays with wild type CtNDT, while varying Hyp with fixed 2´-dAdo. Reactions were performed in duplicate according to the methods reported herein. Left panel depicts raw HPLC data with peaks for compounds detected labelled. Areas were integrated and converted into concentration product formed per unit of time for the Michaelis Menten plot shown on the right.

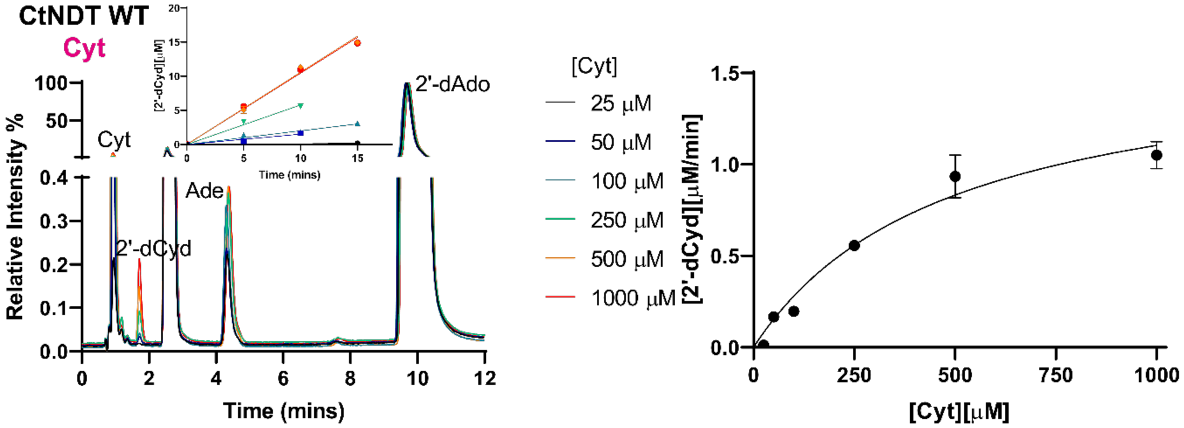

Figure S5j: Initial velocity assays with wild type CtNDT, while varying Cyt with fixed 2´-dAdo. Reactions were performed in duplicate according to the methods reported herein. Left panel depicts raw HPLC data with peaks for compounds detected labelled. Areas were integrated and converted into concentration product formed per unit of time for the Michaelis Menten plot shown on the right.

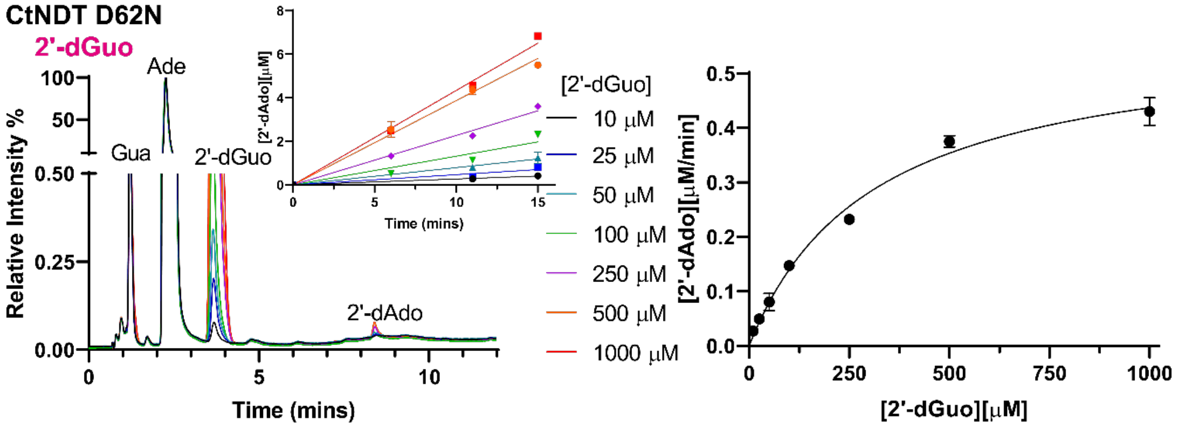

Figure S5k: Initial velocity assays with CtNDT$_{D62N}$ mutant, while varying 2´-dGuo with fixed Ade. Reactions were performed in duplicate according to the methods reported herein. Left panel depicts raw HPLC data with peaks for compounds detected labelled. Areas were integrated and converted into concentration product formed per unit of time for the Michaelis Menten plot shown on the right.

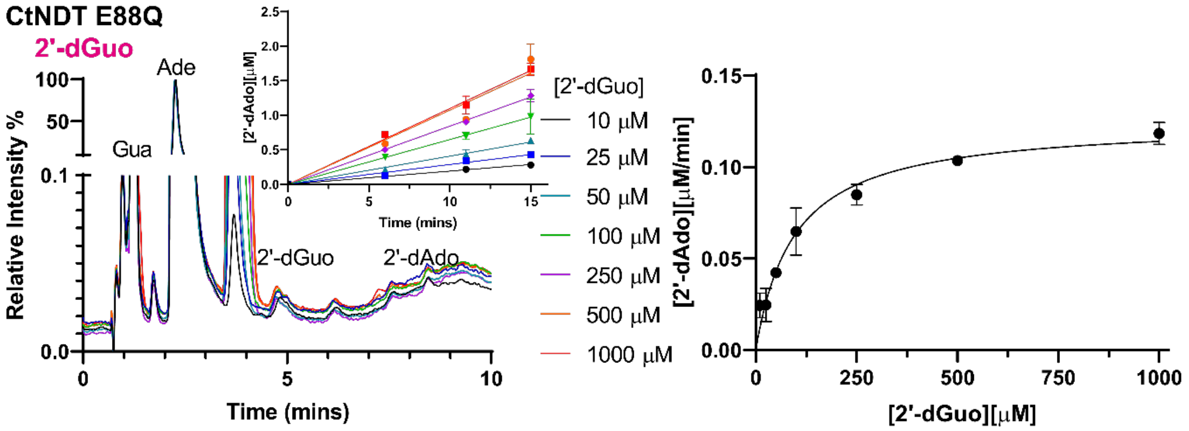

Figure S5l: Initial velocity assays with CtNDT$_{E88Q}$ mutant, while varying 2´-dGuo with fixed Ade. Reactions were performed in duplicate according to the methods reported herein. Left panel depicts raw HPLC data with peaks for compounds detected labelled. Areas were integrated and converted into concentration product formed per unit of time for the Michaelis Menten plot shown on the right.

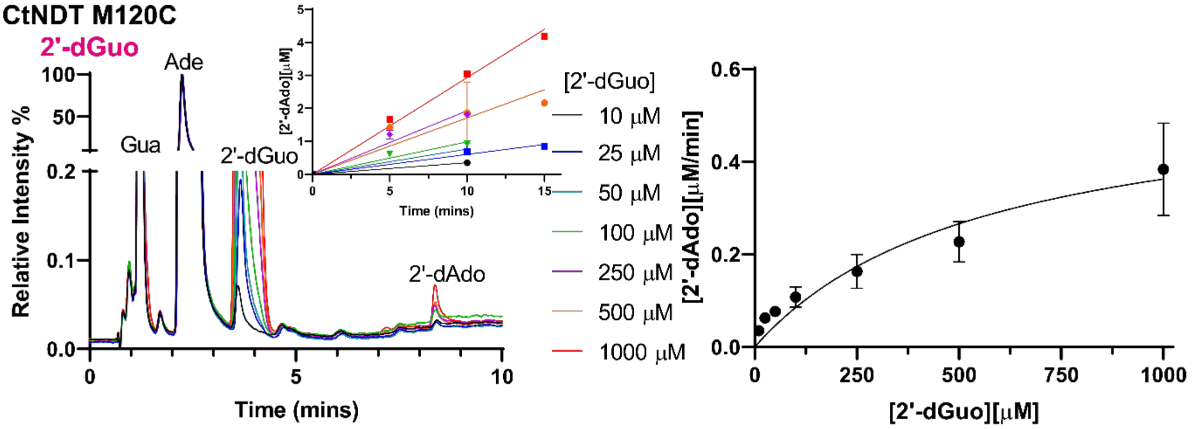

Figure S5m: Initial velocity assays with $CtNDT_{M120C}$ mutant, while varying 2´-dGuo with fixed Ade. Reactions were performed in duplicate according to the methods reported herein. Left panel depicts raw HPLC data with peaks for compounds detected labelled. Areas were integrated and converted into concentration product formed per unit of time for the Michaelis Menten plot shown on the right.

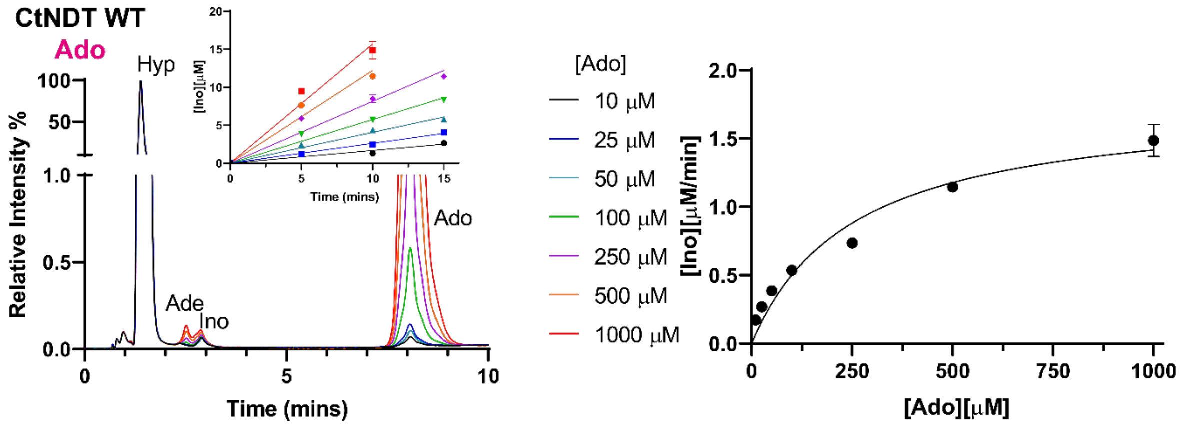

Figure S5n: Initial velocity assays with wild type CtNDT, while varying Ado with fixed Hyp. Reactions were performed in duplicate according to the methods reported herein. Left panel depicts raw HPLC data with peaks for compounds detected labelled. Areas were integrated and converted into concentration product formed per unit of time for the Michaelis Menten plot shown on the right.

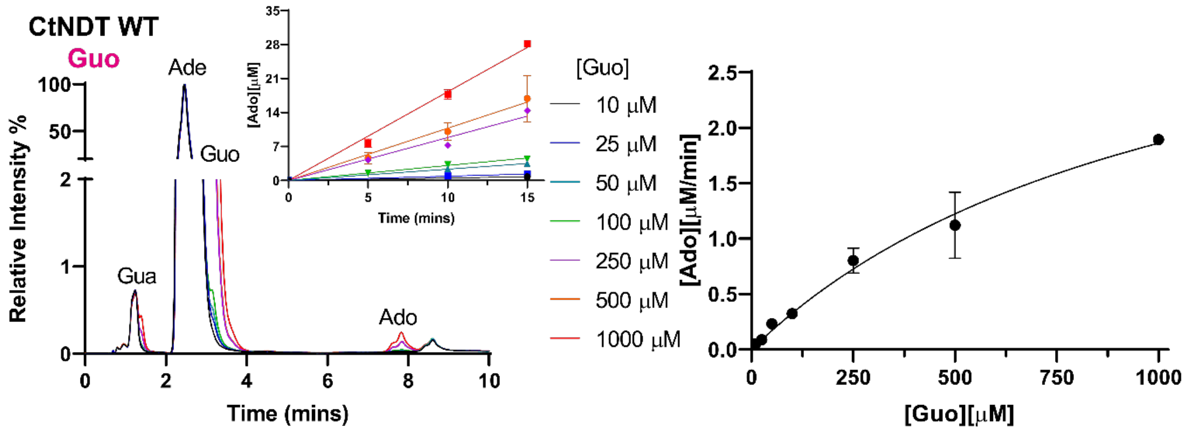

Figure S5o: Initial velocity assays with wild type CtNDT, while varying Guo with fixed Ade. Reactions were performed in duplicate according to the methods reported herein. Left panel depicts raw HPLC data with peaks for compounds detected labelled. Areas were integrated

and converted into concentration product formed per unit of time for the Michaelis Menten plot shown on the right.

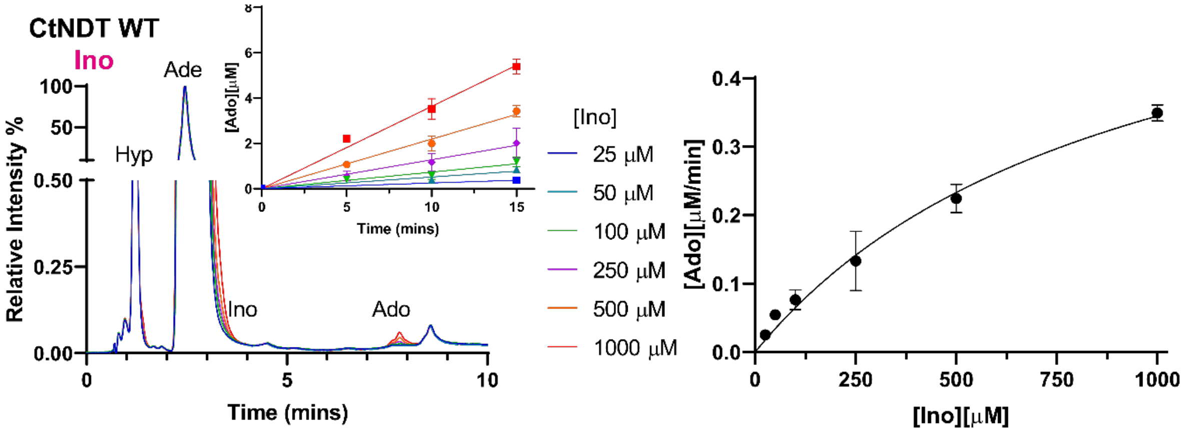

Figure S5p: Initial velocity assays with wild type CtNDT, while varying Ino with fixed Ade. Reactions were performed in duplicate according to the methods reported herein. Left panel depicts raw HPLC data with peaks for compounds detected labelled. Areas were integrated and converted into concentration product formed per unit of time for the Michaelis Menten plot shown on the right.

Stopped flow data analysis

General scheme for reactions monitored:

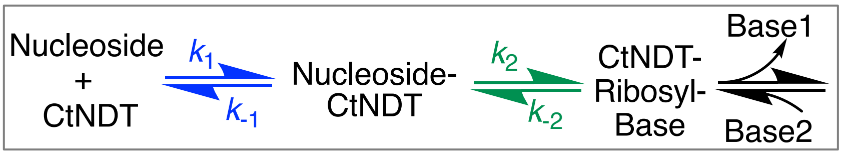

Data were fitted using Kintek Global Explorer. Details for each experiment are as below:

- **2´deoxyadenosine binding to CtNDT$_{E88A}$.**

This experiment was used to determine *the rate constants for* 2´deoxyadenosine binding, k_1 and k_{-1}.

Final concentration of enzyme (after mixing) was 0.75 enzyme and varying ligand as indicated in the figure below:

a)

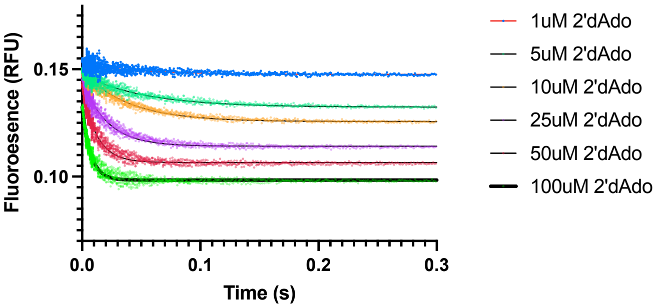

b)

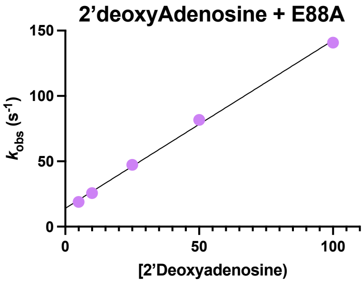

c)

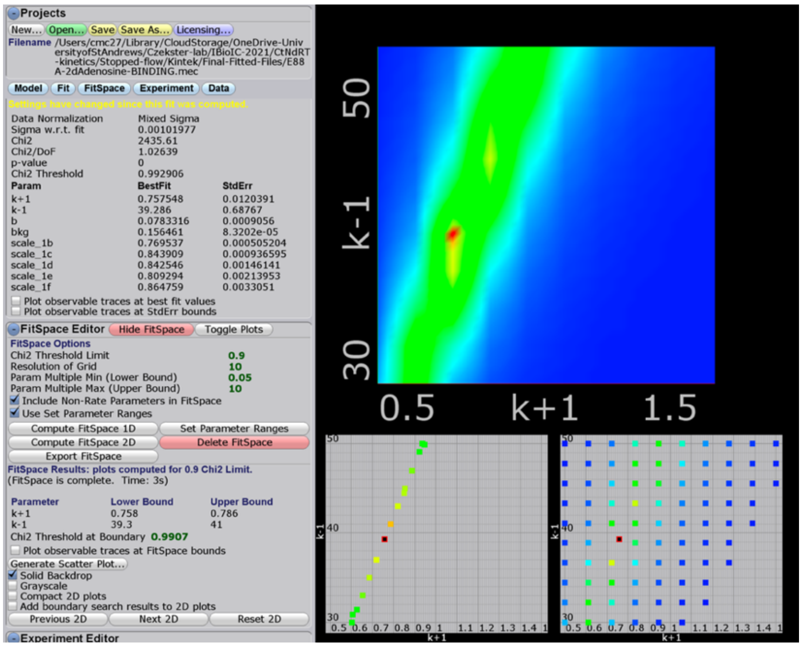

Figure S6: 2´-deoxyadenosine binding to CtNDTE88A.
a) Raw data for binding experiment (dots), line is a fit to a single exponential equation, only used as a starting point for Kintek Global Explorer analysis; b) replot of analytically fitted rate constants in function of ligand concentration. c) Fitspace analysis of the fitted model.

Data were fitted to the model:

E88A + TwodAde = E88A_TwodeoxyAde

Decreased in fluorescence observed upon binding was observed, and the following signal used for fitting:

Observable: scale_1a*((-b*E88A_TwodeoxyAde)+bkg)

- **2´-deoxy adenosine binding to wild type CtNDT.**

Final concentration of enzyme (after mixing) was 0.75 enzyme and varying ligand as indicated below:

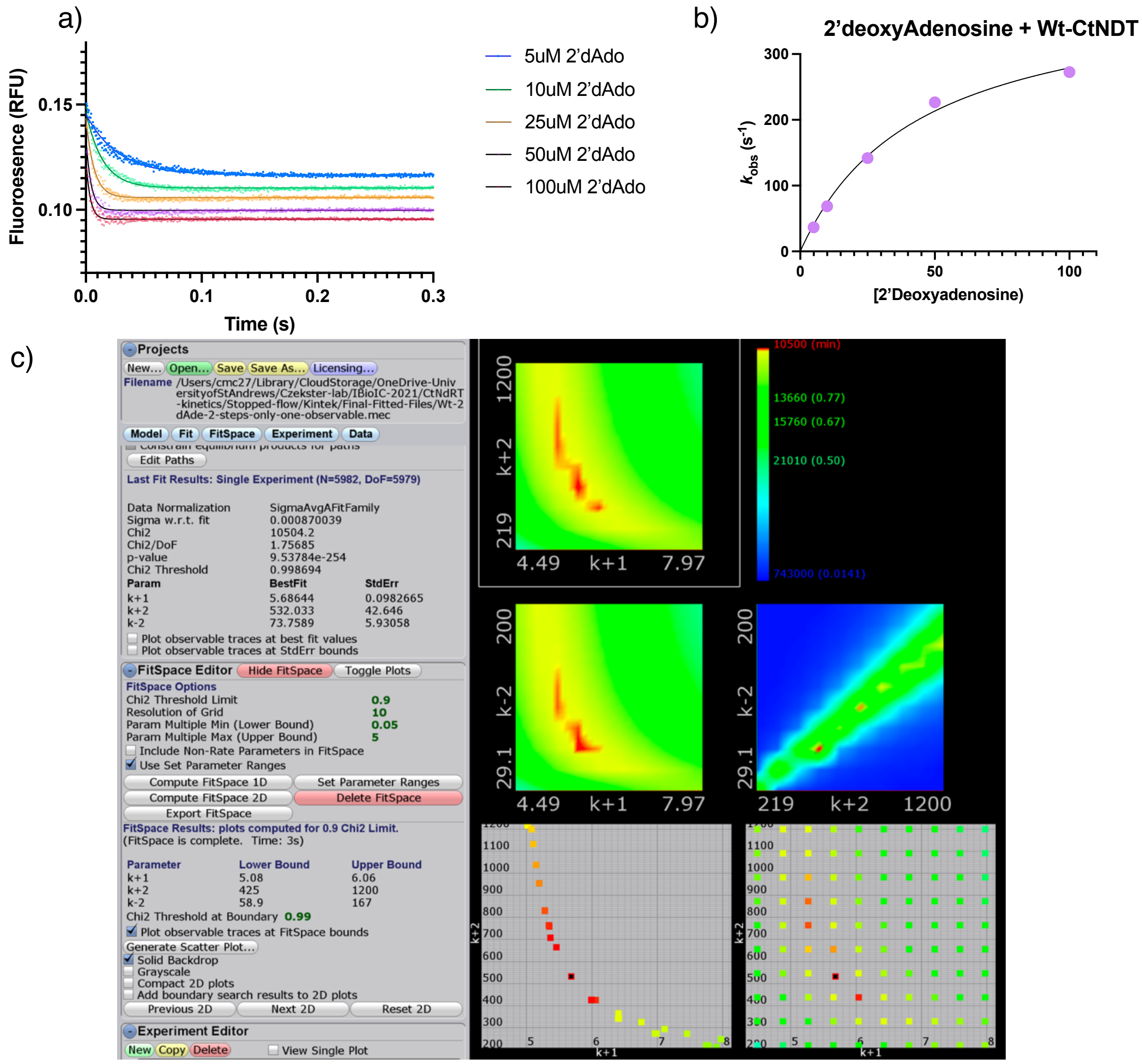

Figure S7: 2´-deoxy adenosine binding to wild type CtNDT.

a) Raw data for binding experiment (dots), line is a fit to a single exponential equation, only used as a starting point for Kintek Global Explorer analysis; b) replot of analytically fitted rate constants in function of ligand concentration. c) Fitspace analysis of the fitted model.

Data were fitted to the model

CtNDT + TwodAde = CtNDT_TwodeoxyAde

CtNDT_TwodeoxyAde = CtNDT_ribosylated

Decreased in fluorescence observed upon binding and ribosylation was observed, and the following signal used for fitting:

a*CtNDT + offset_1a

- **2´-deoxyadenosine binding to CtNDT$_{E88Q}$.**

Final concentration of enzyme (after mixing) was 0.75 mM enzyme and varying ligand as ina) dicated below:

a)

b)

c)

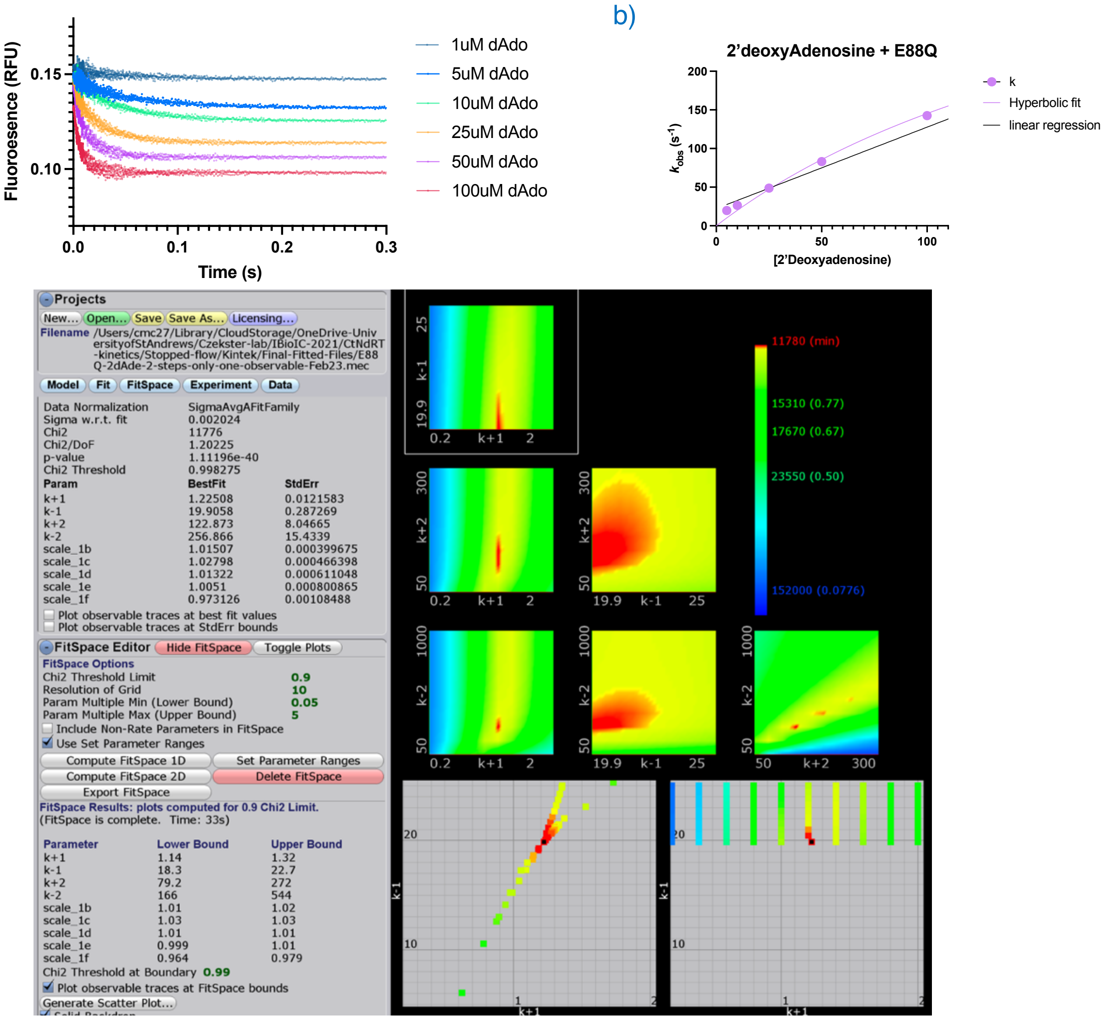

Figure S8: 2´-deoxyadenosine binding to CtNDT$_{E88Q}$.

a) Raw data for binding experiment (dots); b) replot of analytically fitted rate constants in function of ligand concentration. c) Fitspace analysis of the fitted model.

Data were fitted to the model

CtNDT + TwodAde = CtNDT_TwodeoxyAde

CtNDT_TwodeoxyAde = CtNDT_ribosylated

Decreased in fluorescence observed upon binding and ribosylation was observed, and the following signal used for fitting:

scale_1a*(-a*CtNDT_TwodeoxyAde +bkg)

- **2´-deoxyadenosine binding to CtNDT$_{\mathbf{D62N}}$.**

Final concentration of enzyme (after mixing) was 0.75 μM enzyme and varying ligand as indicated below:

a)

c)

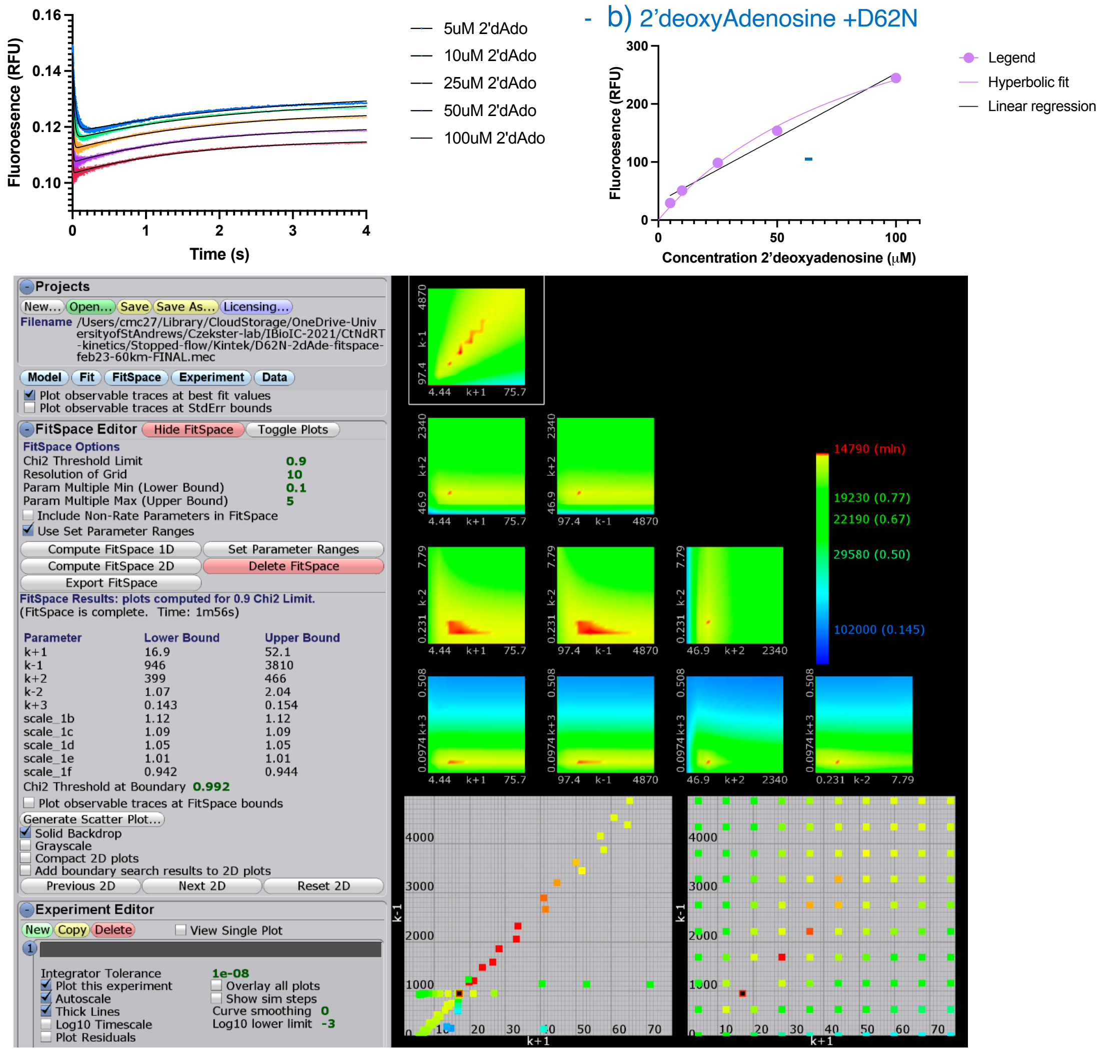

Figure S9: 2´deoxyadenosine binding to CtNDTD62N.

a) Raw data for binding experiment (dots), line is a fit to a double exponential equation, only used as a starting point for Kintek Global Explorer analysis; b) replot

of analytically fitted rate constants in function of ligand concentration. c) Fitspace analysis of the fitted model.

Data were fitted to the model

E + S = ES

ES = EX

EX = F

Decreased in fluorescence observed upon binding and ribosylation was observed followed by a slower increase in fluorescence. Different options for fitting were attempted, but this phase better fitted to a unimolecular process.

The following signal used for fitting:

scale_1a*((-a*EX)+bkg)

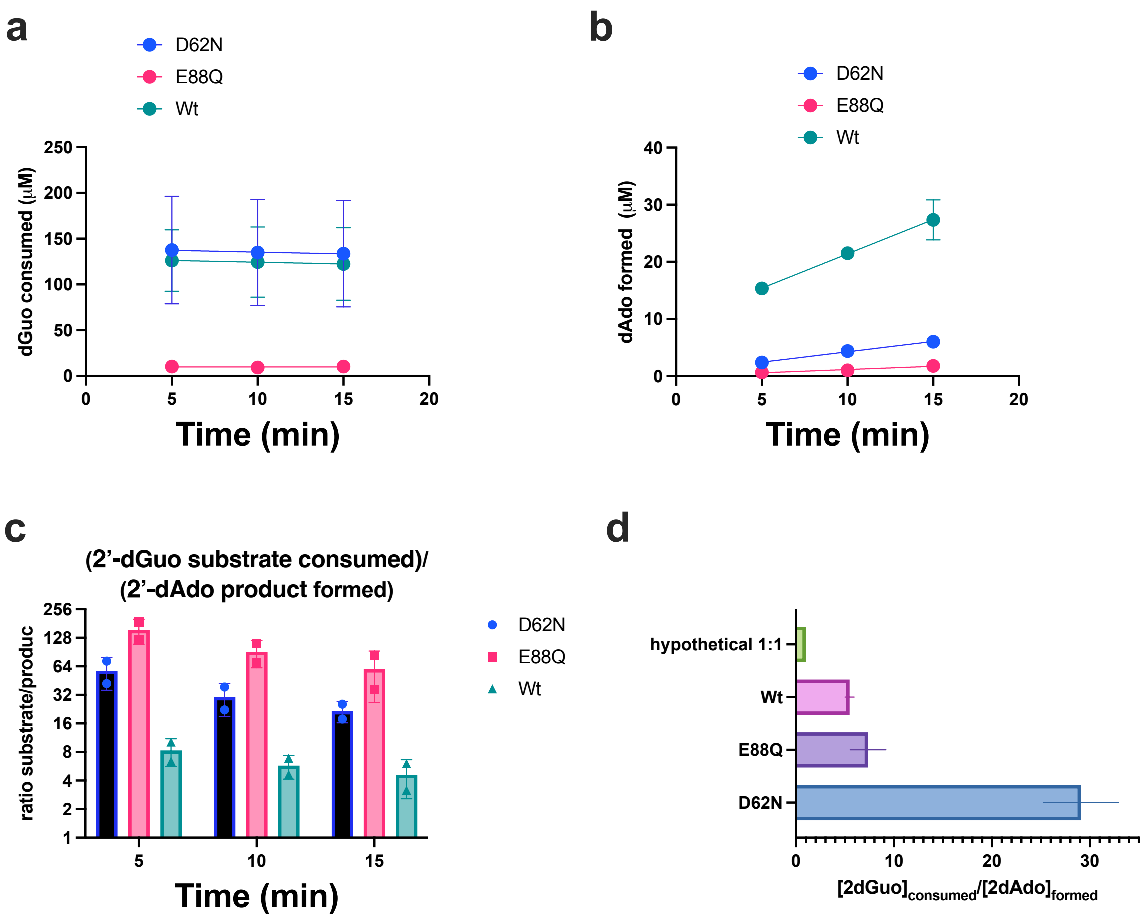

Figure S10: Hydrolysis of nucleoside substrates uncoupled from base transfer: Considerable degree of hydrolysis was observed in all enzyme variants tested. Top) Rates for substrate consumption (left) and 2′-deoxyadenosine product formation (right); Bottom) Ratio 2′-nucleoside formed/2′-nucleoside consumed. Data were collected in duplicate and shown as average plus/minus SD.

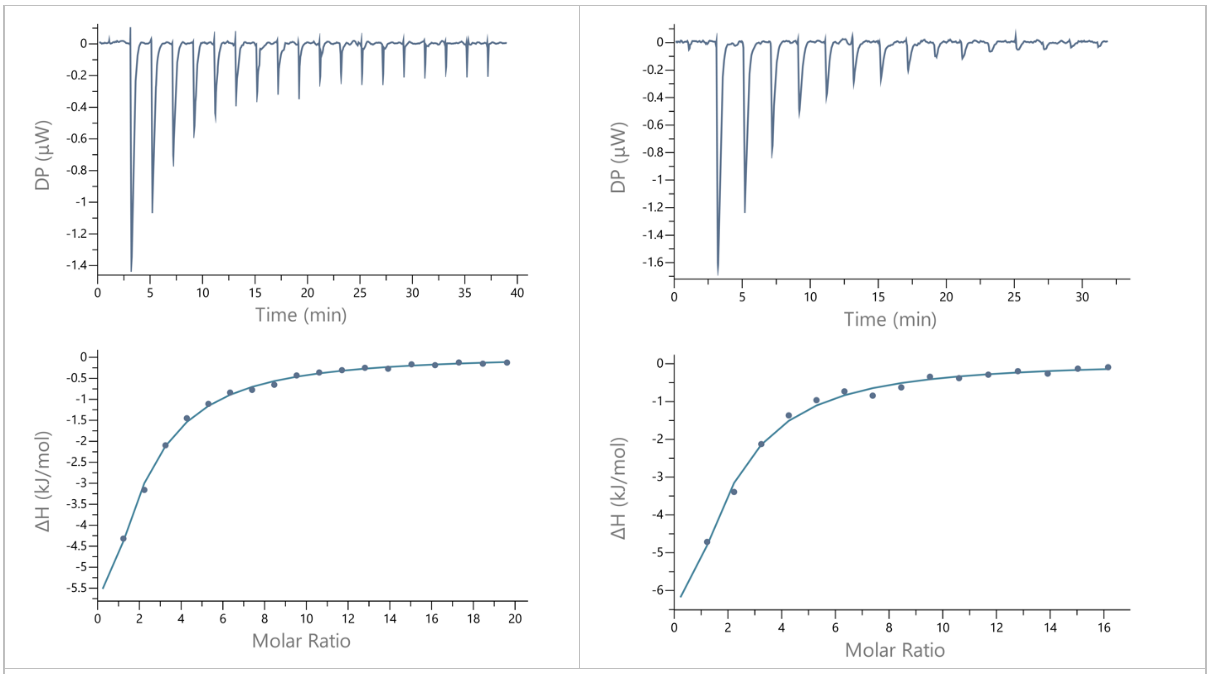

Figure S11 CtNDT (Wt) binding to Immucillin-H: Experiments conducted at 25 degrees, with 30 μM CtNDT and 3000 μM ligand added in 2μl injections.

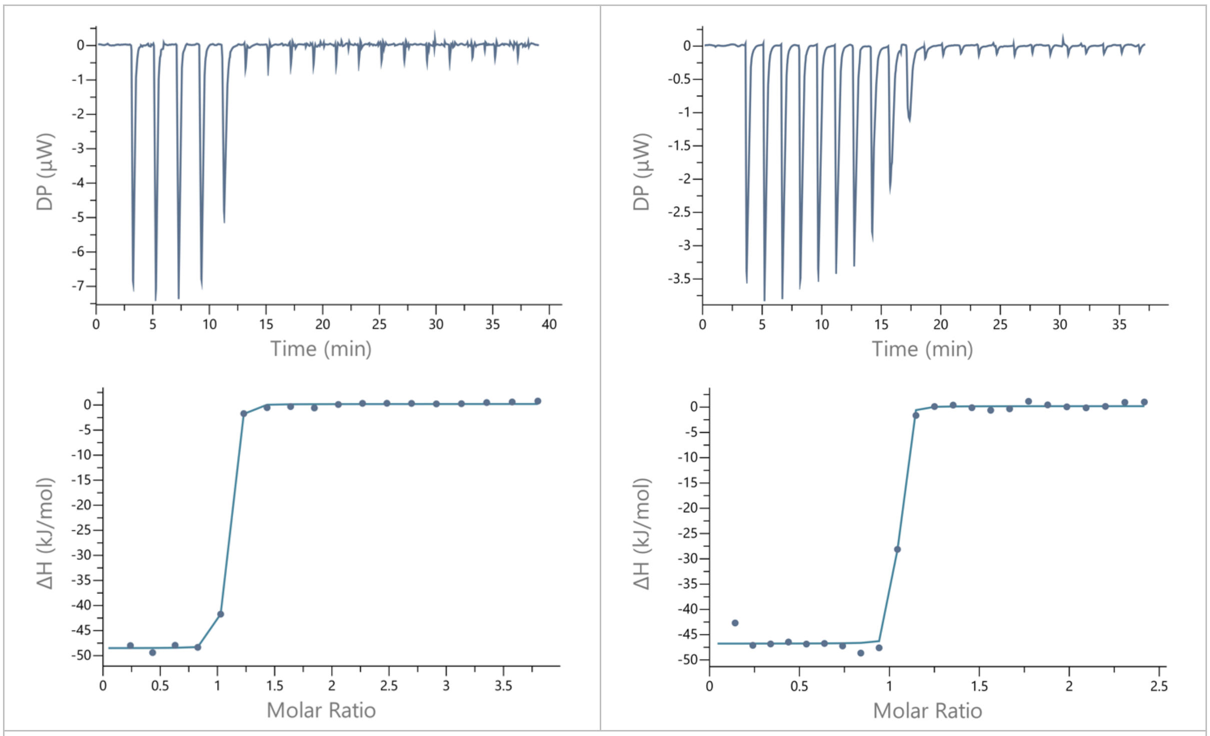

Figure S12 CtNDT (Wt) binding to DAD-Me-Immucillin-H: Experiments conducted at 25 degrees, with 30 μM CtNDT and 1000 μM ligand added in 2 ml injections.

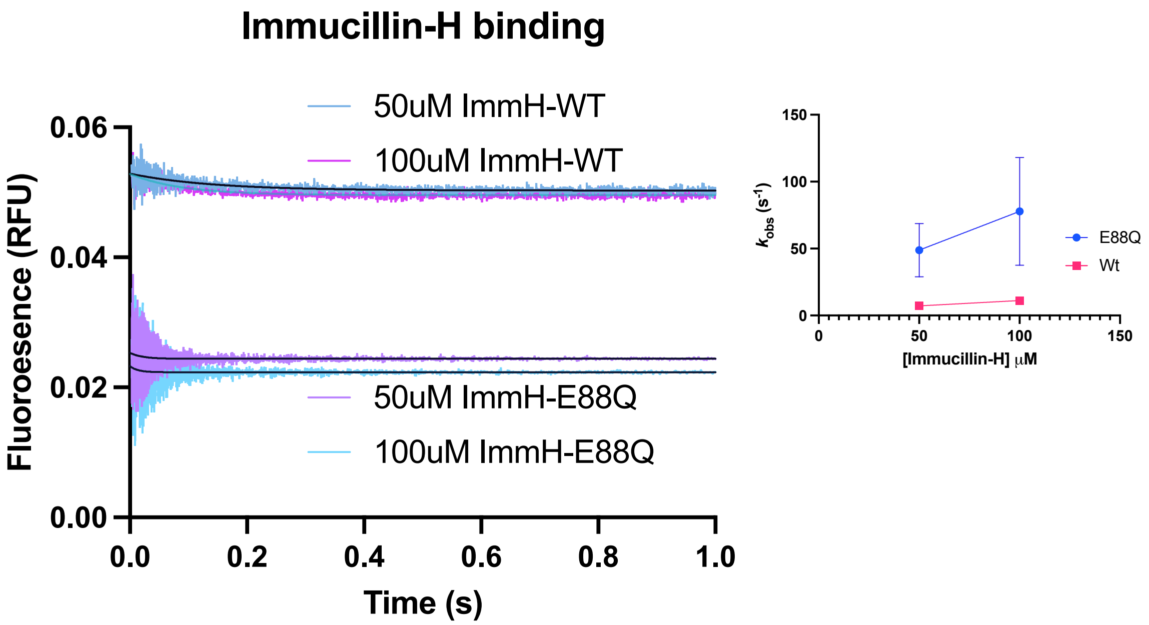

Data fitting on Kintek Global Explorer yielded (model for 1 step binding):

Immucillin H		***boundary***	***K_D***
WT			
k_1	0.06 ± 0.01	0.05 - 0.07	Calculated 76.7
k_{-1}	4.6 ± 0.1	3.7 - 5.7	Experimental 78.2
τ	0.22 s		
E88Q			
k_1	1.8 ± 0.4	0.548 - 176	
k_{-1}	129.3 ± 30.7	28.8 - not defined	Calculated 73.5
τ	0.008 s		

Figure S13: Immucillin-H binding to CtNDT and CtNDT$_{\mathbf{E88Q}}$. Raw data collected with 0.75 μM enzyme and 50 μM and 100 μM ImmH. Inset shows a replot of the analytical fitting to a single exponential equation of each transient. Data for *Ct*NDT$_{E88Q}$ was noisy (with large Fitspace confidence boundaries) and therefore not further interpreted mechanistically.

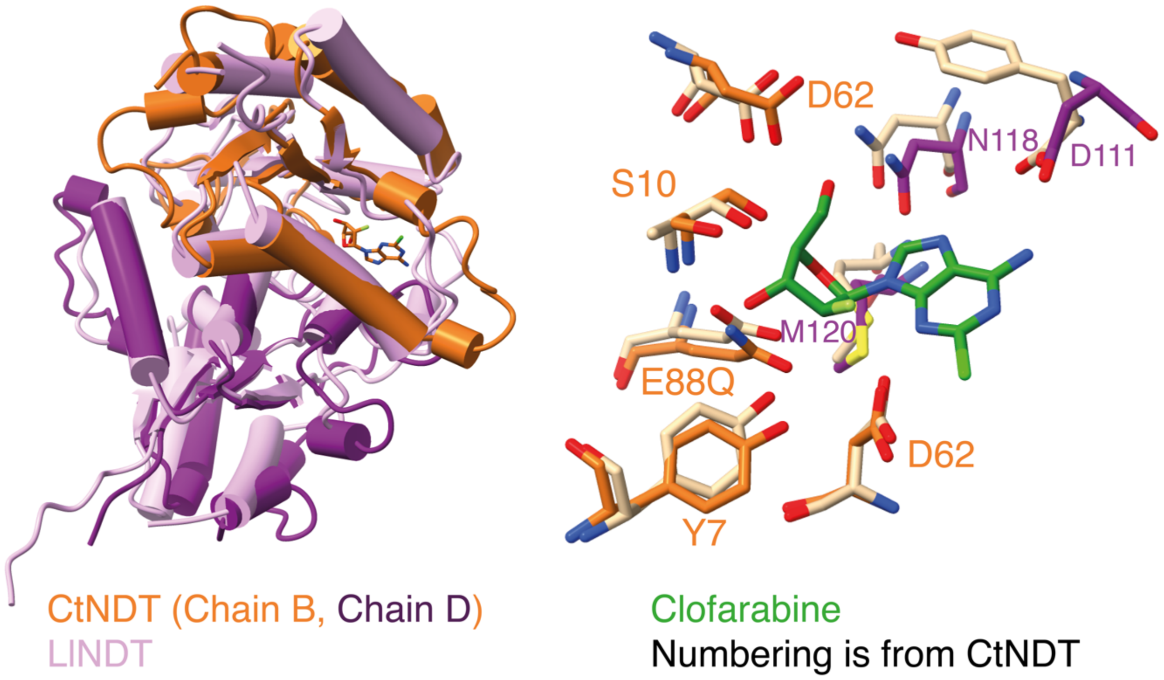

Figure S14: Comparison between substrate binding pocket and active site of CtNDT (orange) and LlNDT (purple, pdb 1F8Y). Key conserved residues are labelled. Overlay was generated using ChimeraX.

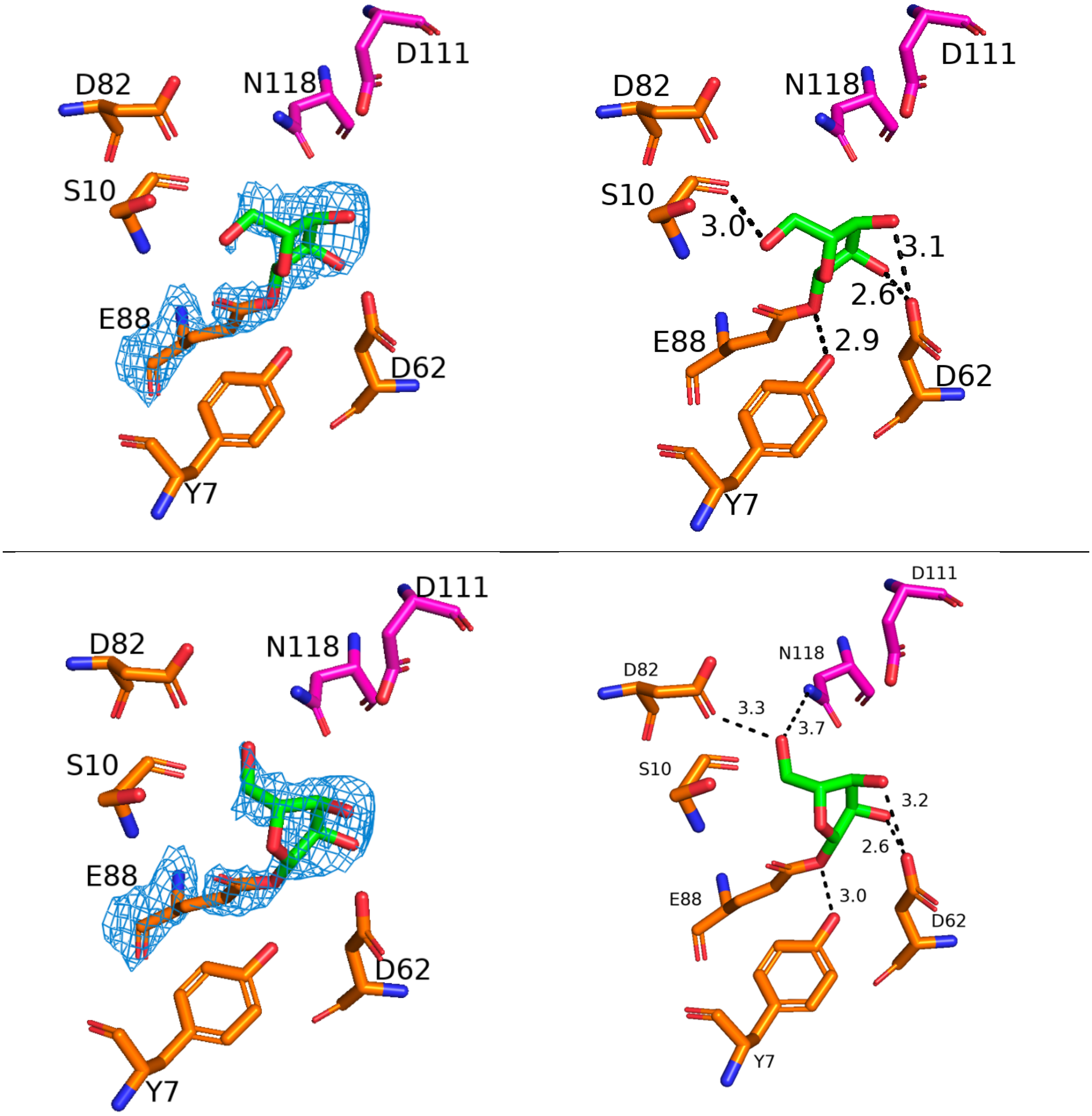

Figure S15: Comparison between alternate conformations of the ribosylated-enzyme intermediate. Top: ribosylated CtNDT, depicting the 5'-OH facing S10. Bottom: ribosylated CtNDT, depicting the 5'-OH facing D82.

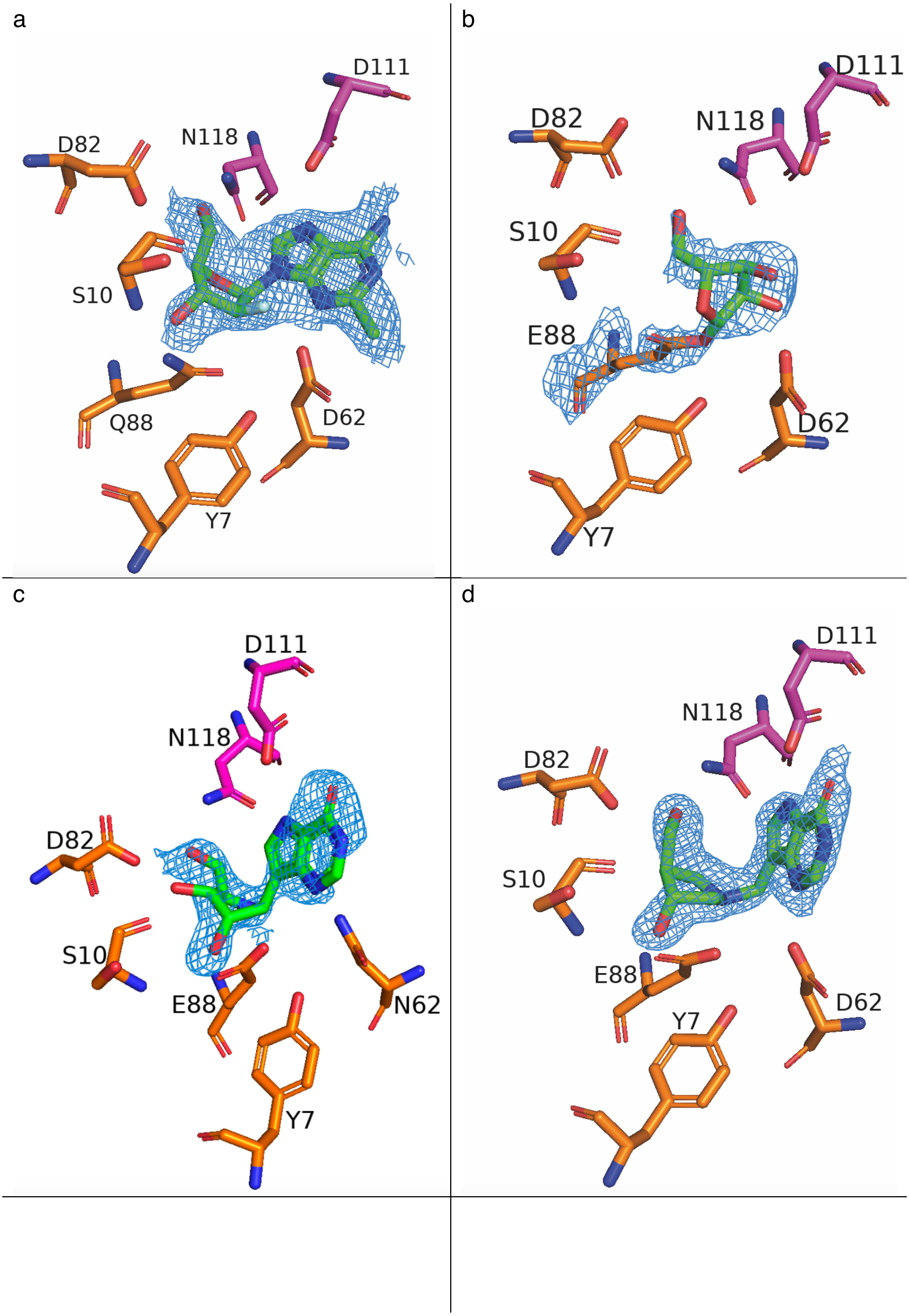
a
D111
D82
N118
S10
Q88
D62
Y7
b
D111
D82
N118
S10
E88
D62
Y7
c
D111
N118
D82
S10
E88
N62
Y7
d
D111
N118
D82
S10
E88
D62
Y7

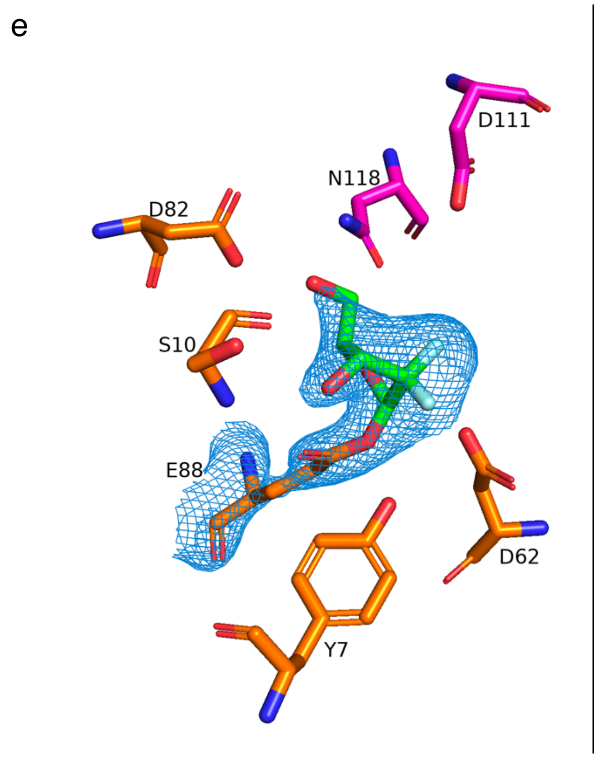

Figure S16: 2Fo-Fc maps at 2s for complex structures reported here. Difference maps were generated using Phenix maximum likelihood maps. a) CtNDTE88Q bound to clofarabine. b) ribosylated CtNDT, depicting the 5'-OH in close proximity to D82 An alternate conformation depicting the 5'-OH facing S10 is depicted on figure S14. c) CtNDTD62N bound to Immucillin-H. d) CtNDT bound to DADmeImmucillin-H. e) 2-difluoro-2-deoxy-ribosylated CtNDT after co crystallization with Gemcitabine.

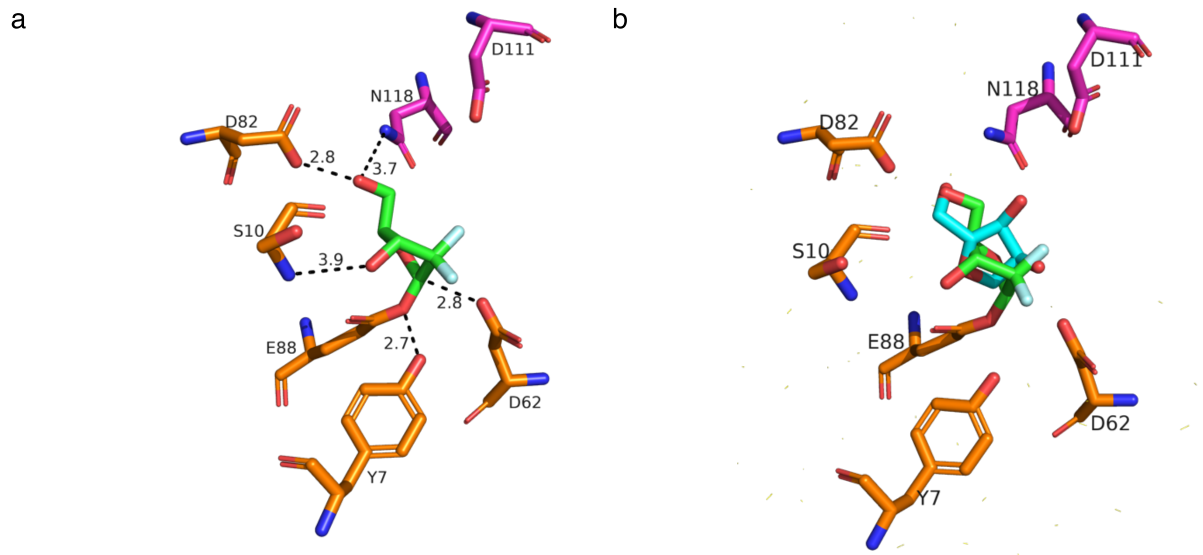

Figure S17: Structure of 2-difluoro-2’-deoxy-ribosylated CtNDT. a) Details on the structure and distances to key residues; b) comparison between 2-difluoro-2’-deoxy-ribosylated (green) and ribosylated (blue) structure, showing the ribosyl 2’ and 3’ OH groups sampling distinct conformations.

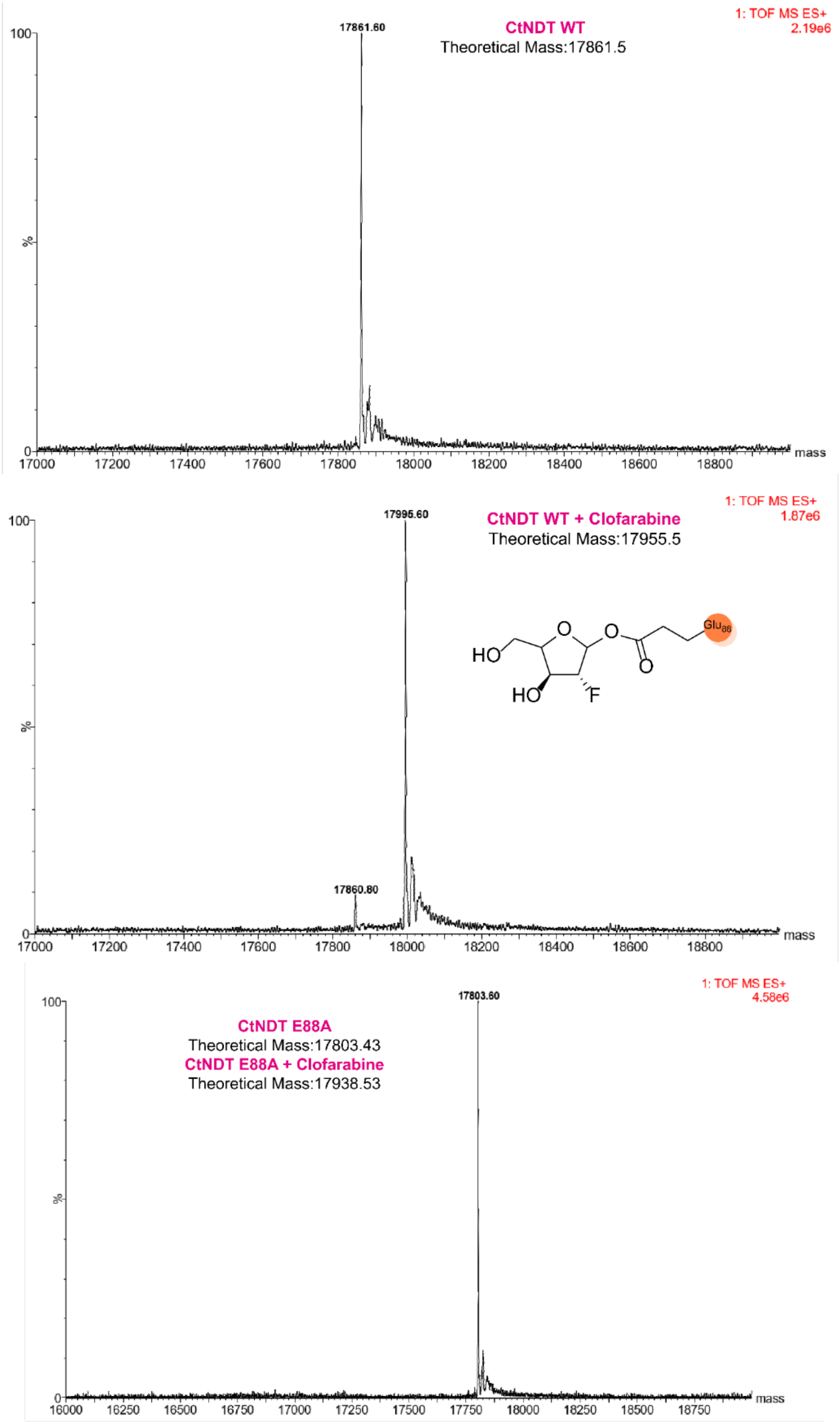

Figure S18: Intact protein mass spectra of CtNDT and its mutants in the absence and presence of Clofarabine. In this experiment, the same protein batch as in Fig. S4 - CtNDT WT, expected MW 17861.5 Da was used. a) mass spectrum of wild type CtNDT which underwent same incubation time as other conditions under comparison without the addition of ligands. b) Protein mass spectrum of wild type CtNDT incubated with 1mM clofarabine. c) Protein mass spectrum of CtNDTE88A incubated with 1mM clofarabine.

Table S1 Primers for cloning and mutagenesis

Name	5´-3´ Primer Sequence
CtNDT Backbone Forward	TTTCCAGGGGATGAAACGTAAGATTATTTACCTTG
CtNDT Backbone Reverse	GAAGCTTTCACATTCCTGTAAGCCATTTATATAATG
pJ411 Forward	TACAGGAATGTGAAAGCTTCCCCCTAGC
pJ411 Reverse	TACGTTTCATCCCCTGGAAATACAAATTTTCAG
E88Q Forward	CGTAATGGTACAATTGGGTATGGCGATTG
E88Q Reverse	CCCTCATCTGGGGGCGTT
E88A Forward	GGTAGCATTGGGTATGGCGATTGCATTAAA
E88A Reverse	CAATGCTACCATTACGCCCTCATCTGG
D62N Forward	GGCACAAGCGAATCTGCAAGATG
D62N Reverse	ACACGGTAGGCCCAGTCA
M120C Forward	GTTAAATCTTTGCTTGTTCGCTGGGCTTCCC
M120C Reverse	GGATAACGCTCGTTGTCAG

Table S2 Steady-state kinetics of CtNDT

**Substrate**	**Acceptor**	**K_M (mM)**	**k_{cat} (s^{-1})**	**k_{cat}/K_M ($mM^{-1}s^{-1}$)**
2´-dAdo	Hyp	0.13 ± 0.03	6. 6 ± 0.5	50.5 ± 12.2
2´-dAdo (25 °C)*	Hyp	0.280	4.6 ± 0.7	15.9 ± 0.8
2´-dGuo	Ade	0.7 ± 0.1	6.9 ± 0.7	10.0 ± 2.3
2´-dIno		1.1 ± 0.2	8.2 ± 0.6	7.3 ± 1.4
2´-dCyd		2.0 ± 0.3	1.7 ± 0.2	0.9 ± 0.2
2´-dUrd		2.2 ± 0.2	0.97 ± 0.05	0.4 ± 0.1
2´-dThd		1.5 ± 0.3	0.17 ± 0.01	0.12 ± 0.03
**Substrate**	**Donor**	**K_M (mM)**	**k_{cat} (s^{-1})**	**k_{cat}/K_M ($mM^{-1}s^{-1}$)**
Ade	2´-dIno	0.2 ± 0.1	13.9 ± 0.8	63.3 ± 12.1
Gua	2´-dAdo	1.4 ± 0.1	29. 7 ± 2.2	21.1 ± 1.6
Hyp		0.6 ± 0.1	8.3 ± 0.1	13.6 ± 1.1
Cyt		0.5 ± 0.1	0.03 ± 0.01	0.06 ± 0.1
Ura		n.d.	n.d.	n.d.
Thy		<LoD	<LoD	<LoD

n.d.: not detected; LoD: limit of detection.

* Determined at 25 °C using Lineweaver-Burke plots for comparison with pre-steady state data.

Table S3 Steady-state kinetics of CtNDT WT with 2´-deoxyribonucloesides and ribonucleosides

WT				
**Donor**	**Acceptor**	K_M **(mM)**	k_{cat} **(s^{-1})**	k_{cat}/K_M **($mM^{-1}s^{-1}$)**
2´-dGuo*	Ade	0.7 ± 0.1	6.9 ± 0.7	10.0 ± 2.3
Ado	Gua	0.25 ± 0.05	0.03 ± 0.02	0.12 ± 0.09
Guo	Ade	1.1 ± 0.3	0.06 ± 0.01	0.06 ± 0.02
Ino		0.9 ± 0.2	0.01 ± 0.01	0.01 ± 0.01

* Reaction carried out at 45°C, while ribonucleoside reactions were at 65°C.

Table S4 Steady-state kinetics of CtNDT and mutants with 2´-dGuo and Adenine

**Enzyme**	$K_M^{2'dGua}$ **(mM)**	$k_{cat}^{2'dGua}$ **(s^{-1})**	$k_{cat}/K_{M-2'dGua}$ **($mM^{-1}s^{-1}$)**
WT	0.69 ± 0.14	6.91	10.01
D62N	0.31 ± 0.04	1.90	6.13
E88Q	0.10 ± 0.02	0.02	0.21
E88A	n.d.	n.d.	n.d.
M120C	0.57 ± 0.27	1.90	3.32

n.d.: not detected

Table S5 Pre-steady state parameters globally fitted using Kintek Global Explorer

	enzyme	Wild type				
		best fitted	error	Fitspace	Chi^2 at boundary	Chi^2 threshold limit
2´dAdenosine binding	k_1 (μ$M^{-1}s^{-1}$)	5.7 ± 0.1		5.19-6.07	0.99	0.9
	k_{-1}-linked to k_1 (s^{-1})	152				
ribosylation	k_2 (s^{-1})	532 ± 43		428-1040		
	k_{-2} (s^{-1})	73.8 ± 5.9		57.1-140		
	**enzyme**	**D62N**				
		**best fitted**	**error**	**Fitspace**	**Chi^2 at boundary**	**Chi^2 threshold limit**
2´dAdenosine binding	k_1 (μ$M^{-1}s^{-1}$)	15.1 ± 0.9		16.9-52.1	0.99	0.9
	k_{-1} (s^{-1})	952.4 ± 83.3		946-3810		
ribosylation	k_2 (s^{-1})	457.8 ± 7.2		399-466		
	k_{-2} (s^{-1})	2.2 ± 0.1		1.07-2.04		
hydrolysis	k_3 (s^{-1})	0.16 ± 0.01		0.143-0.154		
	irreversible					
	**enzyme**	**E88A**				

		best fitted	error	Fitspace	Chi^2 at boundary	Chi^2 threshold limit
2´dAdenosine binding	k_1 ($\mu M^{-1}s^{-1}$)	0.76 ± 0.01		0.758-0.786	0.99	0.9
	k_{-1} (s^{-1})	39.3 ± 0.7		39.3-41		
	**enzyme**	**E88Q**				
		**best fitted**	**error**	**Fitspace**	**Chi^2 at boudary**	**Chi^2 threshold limit**
2´dAdenosine binding	k_1 ($\mu M^{-1}s^{-1}$)	1.22 ± 0.01		1.14-1.32	0.99	0.9
	k_{-1} (s^{-1})	19.9 ± 0.3		18.3-22.7		
ribosylation	k_2 (s^{-1})	122.9 ± 8.0		79.2-272		
	k_{-2} (s^{-1})	256.9 ± 15.4		166-544		

Table S6 Summary of fitted ITC parameters

	ΔH (kcal/mol)		ΔG (kcal/mol)		-TΔS (kcal/mol)		K_D (μM)	
	replicate1	replicate 2	replicate1	replicate 2	replicate1	replicate 2	replicate1	replicate 2
**ImmH**	-5.30	-5.21	-5.54	-5.66	-0.26	-0.46	85.4	70.9
**DADmeImmH**	-11.63	-11.23	-10.75	-11.35	0.90	-0.13	0.0013	0.005
	averaged values ± SD		averaged values ± SD		averaged values ± SD		averaged values ± SD	
**ImmH**	-5.25 ± 0.07		-5.60 ± 0.08		-0.4 ± 0.1		78.2 ± 6.6	
**DADmeImmH**	-11.4 ± 0.3		-11.1 ± 0.4		0.4 ± 0.7		0.009 ± 0.005	

Table S7 Crystallization conditions

PDB accession code	Protein	Protein concentration	**ratio protein/precipitant**	**crystallization condition**	**ligand and concentration for co-crystallization**
8PQS	E88A	9.8 mg/mL	1:1	21.43% PEG 6000; 0.1 M Tris; 0.5 M Zinc Chloride	No ligand
8PQT	WT	9.8 mg/mL	1:1	22.5% v/v PEG Smear Broad; 0.1 M Bis-Tris pH 7.5; 0.05 M Zinc acetate dihydrate; 0.2 M Lithium sulfate	No ligand (Bis-Tris Bound)
8PQQ	E88Q	10 mg/mL	1:1	20% w/v PEG 6000; 0.1 M Tris pH 8; 0.2 M Magnesium chloride hexahydrate	1 mM Clofarabine, co-crystals
8PQP	D62N	11.4 mg/mL	1:1	28% v/v PEG Smear Broad; 0.1 M Sodium Phosphate pH 6.2; 0.2 M Sodium Chloride	1 mM ImmH-Forodesine, co-crystals
8PQR	WT	10 mg/mL	1:1	28% v/v PEG Smear Broad; 0.1 M Tris pH 8.5; 0.15 M Ammonium Acetate; 0.01 M Calcium chloride dihydrate	1 mM DAD-me-ImmH, co-crystals
8QC0	WT	10 mg/mL	1:1	21.43% PEG 6000; 0.1 M Tris; 0.5 M Zinc Chloride	1 mM Adenosine, co-crystals
8RH3	WT	10 mg/mL	1:1	30.63% v/v PEG 6000; 0.1 M Tris pH 8; 0.00030 M Zinc chloride	1 mM Gemcitabine, co-crystals

Table S8 Crystallographic Data Table

	**8QC0** *Ct*NDT Ribosylated	**8RH3** *Ct*NDT 2F2dRibosylated	**8PQR** *Ct*NDT bound to DADmeH	**8PQP** *Ct*NDT$_{D62N}$ bound to ImmH	**8PQQ** *Ct*NDT$_{E88Q}$ bound to Clofarabine	**8PQT** *Ct*NDT bound to Bis-Tris	**8PQS** *Ct*NDT$_{E88A}$ apo
Wavelength	0.9763	0.9763	0.9795	0.9795	0.9762	0.9795	0.9763
Resolution range (Å)	44.27 - 2.02 (2.092 - 2.02)	70.09 - 1.963 (2.033 - 1.963)	51.88 - 1.586 (1.643 - 1.586)	49.8 - 1.709 (1.77 - 1.709)	48.87 - 2.23 (2.31 - 2.23)	52.36 - 1.701 (1.762 - 1.701)	44.4 - 1.95 (2.02 - 1.95)
Space group	P 63	P 63	P 32	P 32 2 1	P 63	P 62 2 2	P 63
Unit cell (a,b,c) (Å)	135.24, 135.24, 87.22	136.092 136.092 87.1724	97.28, 97.28, 65.85	93.57, 93.57, 63.12	136.1, 136.1, 87.44	131.19, 131.19, 86.94	135.63, 135.63, 87.56
α, β, γ (°)	90, 90, 120	90, 90, 120	90, 90, 120	90, 90, 120	90, 90, 120	90, 90, 120	90, 90, 120
Unique reflections	59511 (5912)	69176 (6549)	93642 (8689)	34153 (2819)	45008 (4506)	48780 (4791)	66772 (6667)
Multiplicity	39.2	39	10.1	16.7	21.03	50.9	39.2
Completeness (%)	99.89 (99.27)	99.98 (98.89)	99.16 (91.80)	97.95 (81.59)	99.96 (99.91)	99.94 (99.85)	99.97 (99.90)
Mean I/sigma(I)	15.6	12.3	8.7	21.8	11.2	19.6	13.7
Wilson B-factor	47.97	52.2	27.96	40.04	48.28	25.03	38.72
R-merge	0.192	0.164	0.121	0.049	0.224	0.145	0.28
R-meas	0.194	0.166	0.127	0.05	0.23	0.146	0.283
R-pim	0.031	0.027	0.039	0.011	0.05	0.02	0.045
CC1/2	0.999	1	0.998	0.999	0.995	1	0.998
Reflections used in refinement	59456 (5869)	59878 (1447)	93627 (8687)	34134 (2818)	44998 (4502)	48755 (4785)	66760 (6660)
Reflections used for R-free	2958 (294)	2946 (67)	4538 (393)	1665 (163)	2131 (234)	2485 (258)	3239 (321)
R-work	0.2178 (0.4475)	0.2081 (0.4212)	0.1715 (0.4375)	0.1889 (0.4211)	0.1830 (0.3215)	0.1980 (0.2862)	0.1991 (0.3368)
R-free	0.2197 (0.4081)	0.2552 (0.3885)	0.1964 (0.4649)	0.2146 (0.4380)	0.2232 (0.3549)	0.2325 (0.2959)	0.2243 (0.3649)

Number of non-hydrogen atoms	5202	5394	5534	2597	5283	2780	5331
macromolecules	4931	4936	4988	2485	4981	2453	4940
ligands	40	40	76	19	82	66	0
solvent	231	418	470	93	220	299	391
Protein residues	609	607	614	308	615	298	611
RMS(bonds)	0.012	0.079	0.007	0.007	0.009	0.01	0.007
RMS(angles)	1.74	3.85	1.21	1.18	1.27	1.15	0.89
Ramachandran favored (%)	98.16	97.32	98.35	98.03	98.35	99.32	99.17
Ramachandran allowed (%)	1.67	2.18	1.65	1.97	1.65	0.68	0.83
Ramachandran outliers (%)	0.17	0.5	0	0	0	0	0
Rotamer outliers (%)	3.47	0.58	0.76	0	0.76	0.39	0.19
Clashscore	3.57	6.72	2.51	2.03	4.22	2.23	3.38
Average B-factor	53.01	57.73	40.91	61	60.17	19.67	44.82
macromolecules	52.89	57.12	40	60.99	59.64	18.08	44.5
ligands	58.69	57.92	53.88	88.46	101.98	14.89	
solvent	54.5	64.94	48.48	55.57	56.58	33.13	48.91
Number of TLS groups			29	8	24		

*Values in parentheses are for the high-resolution shell
**R-value test set size = 5%
